# Adenylyl cyclase 9: Fundamental change of regulation in vertebrates and gene sub-functionalization after teleost-specific whole-genome duplication

**DOI:** 10.1016/j.isci.2026.116562

**Published:** 2026-07-07

**Authors:** Ferenc A. Antoni, Julie Mazzolini, Heather McClafferty, Zhiaho Chen, Laura Szalai, Cristina Xia, Sahad Iqbal, Martin Denvir, András Balla, Dirk Sieger, Michael J. Shipston, Paul Skehel

**Affiliations:** 1 Centre for Discovery Brain Sciences, Institute of Neuroscience and Cardiovascular Research, University of Edinburgh, Edinburgh EH9 9XD, Scotland, UK; 2 Department of Physiology, Semmelweis University, 1094 Budapest, Hungary; 3 Cardiovascular Research Centre, Institute of Neuroscience and Cardiovascular Research, University of Edinburgh, Edinburgh EH16 4TJ, Scotland, UK

**Keywords:** adenylyl cyclase, auto-inhibition, G protein coupled receptors, whole genome duplication, zebrafish, medaka

## Abstract

In mammals, transmembrane adenylyl cyclase 9 (AC9) is resistant to regulation by cell surface receptors coupled to heterotrimeric G proteins. A major facet of this resistance is auto-inhibition—in the presence of activated Gsa, AC9 is inhibited by its C-terminal domain. Here, we examined the evolution of this seemingly paradoxical control mechanism. The hallmarks of auto-inhibition are apparent in all vertebrates; none are found in invertebrates. Teleost-specific whole genome duplication (TGD) resulted in *adcy9* ohnologs, one of which lacked the hallmarks of auto-inhibition, which was confirmed in functional assays. The tissue distributions of the *adcy9* ohnologs in teleost species pointed to their sub-functionalization. Auto-inhibited *adcy9* was largely restricted to the brain, indicating a significant role in brain function. We document a fundamental change in the regulation of the enzymatic activity of AC9 and the potency of TGD to meet an adaptational challenge through functionally diversified ohnologs.

## Highlights

• A new mode of autoregulation of adenylyl cyclase 9 appeared in vertebrates
• Whole-genome duplication produced sub-functionalized *adcy9* ohnologues in teleost fish
• Expression of the autoinhibited *adcy9a* ohnologue is largely restricted to the brain
• In contrast, autoinhibition appears to be an evolutionary burden in the heart

## Graphical abstract

**Figure:**
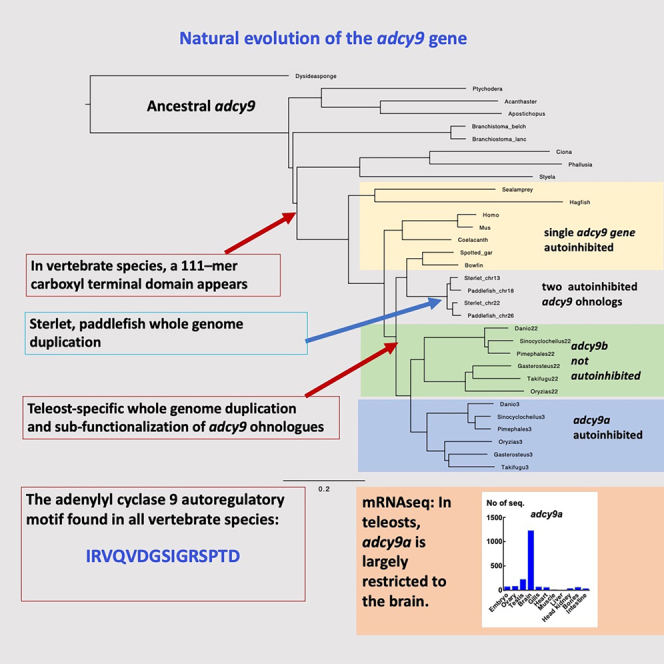

## Teaser

Genetics; Molecular biology; Evolutionary biology; Cell biology

## Introduction

**Figure 1:**
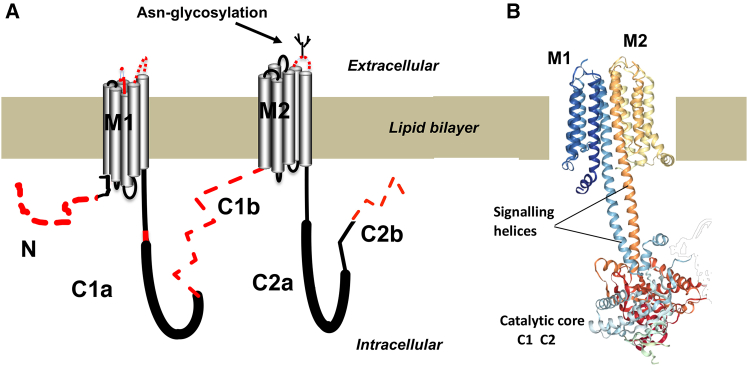
Schematic representations of adenylyl cyclase 9 (AC9) (A) A single polypeptide with two six-member transmembrane (M1 and M2) arrays. Dashed red lines indicate where the cryoEM failed to clarify the structure. Thick lines indicate the catalytic core that is similar in all paralogs of AC9. N—amino-terminal domain, C1b and C2b are isoform-specific cytoplasmic loops. (B) The 3D structure of AC9 obtained by cryoEM. Note there are three structurally distinct regions: the transmembrane arrays, the signaling helices, and the cytoplasmic catalytic domain formed by C1 and C2.

Signaling through adenosine 3’:5′monophosphate (cAMP) is close to ubiquitous in biological systems. In vertebrates, ten genes encode the enzyme of cAMP biosynthesis, adenylyl cyclase (AC). Nine of these give rise to transmembrane proteins (AC1 to AC9) while one gene product is a soluble enzyme.^1^^,^^2^^,^^3^ All eukaryotic transmembrane ACs (tmACs) conform to the same blueprint of a single polypeptide chain of quasi-symmetrical design, in which two sets of six closely spaced transmembrane domains are preceded, linked, and terminated by long cytoplasmic and relatively short extracellular loops (Figure 1A). High-resolution cryo-electronmicroscopic maps of AC5, AC8, and AC9 all show that tmACs consist of three structurally distinct, functionally connected domains (Figure 1B).^4^ The transmembrane arrays are closely packed and interfaced in the cellular lipid bilayer. Transmembrane helices 6 and 12 face each other and continue into the cytoplasm as alpha-helices (signaling helix 6 and 12, respectively), forming a short parallel coiled-coil before the start of the C1a_C2a catalytic core. The isoform-specific N cytoplasmic loop, the C1b and most of the C2b domain could not be resolved by cryoEM, most plausibly because these are intrinsically disordered domains (Figure S1). Importantly, a short segment of the C2b domain could be detected, yielding the striking finding that in the presence of activated Gsα, the active site of the enzyme is occupied by a previously identified short autoregulatory motif (ARM)^5^ comprised of 15 amino acid residues.^6^^,^^7^ Removal of the ARM markedly enhanced the stimulation of AC9 by Gsα in a cell-free system^6^ as well as its response to the activation of receptors coupled to Gs in intact cells.^5^^,^^8^

An ancestral AC9 (*adcy-1* gene) is already present in *C. elegans*.^9^^,^^10^ This enzyme has virtually no C2b domain and is readily stimulated by Gs-coupled receptors as well as forskolin.^9^^,^^11^^,^^12^ Thus, the marked autoinhibition of physiological stimulation in mammalian AC9 orthologs appears to be a veritable biological paradox worthy of further analysis with particular reference to its origin during the course of natural evolution.

## Results

### ARM is specific to the vertebrate subphylum

A *blastp* search with residues 1243–1353 of human AC9 (the ARM motif is found at residues 1261–1273) returned 1579 hits. The alignment of these sequences showed a close to 100% conservation of the ARM (Data S1). The main consistent change found was at position 1264 (to A or I), and was restricted to ray-fin fish. Upon the exclusion of vertebrates, no hits other than synthetic constructs were found, either using the DNA sequence with the *megablast* protocol or the protein sequence with settings of *tblastn* to find less similar sequences.

The phylogenetic tree of selected AC9 protein sequences is shown in Figure S2. The node of the split between invertebrates and vertebrates is clearly apparent.

**Figure 2:**
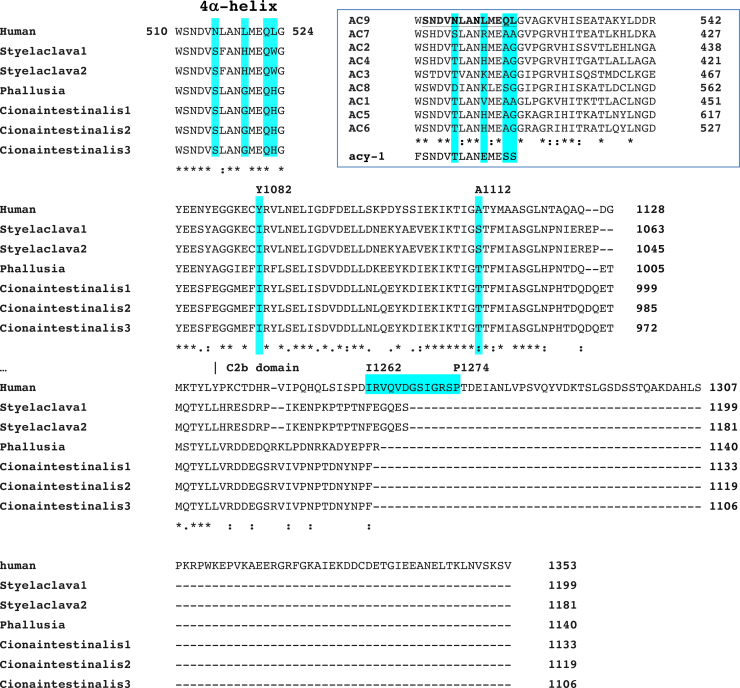
Alignment of relevant sections of the primary sequence of human adenylyl cyclase 9 with its predicted orthologs from *tunicata,* the invertebrate species phylogenetically closest to vertebrates (see also Figure S3 for justification) Numbering refers to human AC9. Note: (1) The lack of a sizable C2b domain in *tunicata;* (2) The presence of three residues in the 4-α helix that are unique to the human enzyme. In fact, these are 100% conserved vertebrate orthologs of AC9; (3) The forskolin responsive configuration in *tunicata* at the homologs of human AC9 Y1082 and A1112. **INSERT:** Shows the alignment of human AC paralogs in the area of the 4-α helix (in bold) in the C1a domain. Note residues unique to AC9 highlighted in cyan, which are fully conserved in the orthologs of AC9 of vertebrate species. At the bottom, acy-1 is the homologous segment of the AC9 ortholog of *C. elegans*, which is responsive to forskolin. It has closer sequence similarity with the non-AC9 residues than with AC9.

A comparison of human AC9 with the AC9 orthologs of *tunicata,* the invertebrate subphylum most closely related to vertebrates, is shown in Figure 2. The most conspicuous change is the appearance of the long C2b domain harboring the ARM. Moreover, modifications at positions N515, L519, Q522, L523 (human AC9 numbering) of the 4-alpha helix of the C1a domain, the main element of the forskolin binding-pocket, are evident. Further relevant changes are at Y1082 and A1112, both previously shown to influence the response of mouse AC9 to forskolin.^13^ The changes listed above are 100% conserved in the AC9 orthologs of vertebrates. The AC9 orthologs of cyclostomi, such as the hagfish (*Eptatretus burger)*, the sea-lamprey (*Petromyzon marinu),* and the arctic lamprey (*Lethenteron camtschaticum,* also known as *Lethenteron japonicum)*) already feature the full complement of the changes listed above for human AC9 (Figure S3). Specifically, the long C2b domain is present and includes the highly conserved ARM and its immediate sequence environment. The contact sites for ARM^6^ in the 4α-helix of the C1a catalytic domain have the four AC9-specific residues. In addition, the low forskolin response configuration^13^^,^^14^ at Y1082 and A1112 in human AC9 is also conserved in all three of the cyclostome orthologues. Taken together, the hallmarks of autoregulation in the primary structure of AC9 are already apparent in the oldest species of currently living vertebrates. Moreover, the ARM, as a highly conserved segment of a long, isoform-specific C2b domain of AC9, appears to be specific to and highly conserved in the vertebrate subphylum.

### Teleost fish have *adcy9* ohnologs

Searches with *tblastn* for the entire human AC9 primary sequence revealed that several ray-finned species have two AC9 genes. Ray-fin species such as the bowfin (*Amia calva*) and the spotted gar (*Lepisosteus oculatus*) that appeared prior to teleost-specific genome duplication (TGD), (see^15^^,^^16^ for reviews) have a single AC9 gene (*adcy9a*) encoding a protein with an ARM that closely resembles the human version (Data S1). Currently living teIeosts have undergone TGD (e.g., the zebrafish) and have at least two *adcy9* genes. That these are ohnolog pairs has been established for at least four species, including zebrafish and medaka.^17^ There is invariably an *adcy9a* gene that contains the ARM, and usually has some subtle modifications, most notably V1264A within ARM and W1158G in the C2a catalytic domain, when compared to the mammalian and pre-TGD counterparts of fish. The latter mutation affects a W residue unique to AC9 that participates in the binding of activated Gsα to bovine AC9.^18^ Tryptophan at this position is shared by all mammalian AC9 orthologs. More significantly, the ohnolog *adcy9b* encodes a cyclase protein (AC9b) that has a long C2b domain, but lacks the ARM sequence. In AC9b, the residues specific to mammalian AC9 listed in the previous section are in the configuration found in AC9a—i.e., low forskolin response. *Salmo trutta*, and goldfish (*Carassus auratus*), which have undergone salmonid and cyprid-specific genome duplications after TGD, respectively,^19^ have four *adcy9* genes on different chromosomes, two each of *adcy9a-*like and *adcy9b*-like genes, respectively.

A different set of *adcy9* genes is found in the sterlet (*Acipenser ruthenus*). The two *adcy9* genes on chromosomes 13 and 22, respectively, contain the ARM i.e., are *adcy9a*-like. Similarly, in paddlefish (*Polydon spatula*), there are two *adcy9a*-like genes on chromosomes 18 and 26 (Data S1). The post-TGD modifications of V1264A within ARM and W1158G in the C2a catalytic domain are not present in any of the predicted proteins (Data S1). These findings are consonant with the report showing the ancestor of the sturgeon-paddlefish lineages had undergone whole genome duplication and indicate that the *adcy9* genes have remained unaltered after the separation of the sturgeon and paddlefish lineages.^20^ Taken together, TGD has resulted in the ohnolog genes *adcy9a* and *adcy9b.* The primary sequences of the encoded proteins are distinguished by the presence or absence of the ARM in the long C2b domain. The phylogenetic tree (Figure S2) of AC9 highlights that there are at least two lineages in teleosts. These can be readily discerned by the C2b domain of AC9b: BLAST searches with the respective C2b domain sequences of AC9b of *D. rerio* and *O. latipes* gave different, non-overlapping sets of hits.

### Differential expression of *adcy9a* and *adcy9b* in tissues indicates sub-functionalization

**Figure 3:**
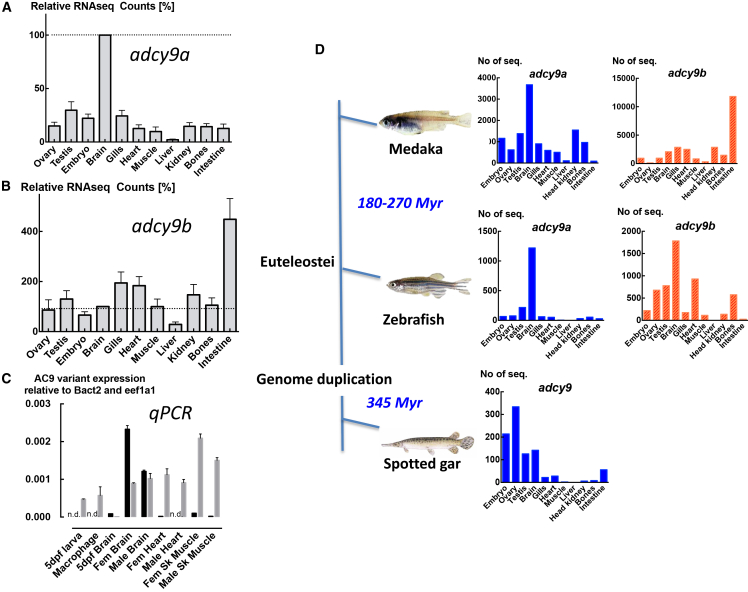
Expression of the *adcy9* genes in the tissues of bony fish (A and B) RNA seq hits from the PhyloFish database. The values are expressed as a percentage of the number of respective hits from the brain. For *adcy9a* this was 2,623 ± 355, for *adcy9b* 845 ± 184 Mean ± SEM. N = 14. (C) RT-qPCR data for the relative abundance of *adcy9a* and *b* mRNAs from zebrafish tissues and 5 d.p.f. whole embryos—filled columns *adcy9a*, empty columns *adcy9b*. Data are means ± SD, *n* = 5 technical replicates per group. (D) The timeline of fish through TGD. The graphs represent the number of RNA seq hits from the PhyloFish database for *adcy9a* and *b*.

The Phylofish database “Depth View” feature was used to assemble a semi-quantitative comparison of the distribution of the two *adcy9* gene transcripts in the tissues of post-TGD teleosts on the basis of the number of mRNAseq hits. Importantly, *adcy9a* mRNA was largely restricted to the brain while *adcy9b* mRNA predominated in all peripheral tissues examined (Figures 3A and 3B). Follow-up analysis by RT-qPCR of selected zebrafish tissues supported this notion: Of the tissues examined, only the brain—adult as well as 5 d.p.f. larva—showed relatively high levels of *adcy9a* mRNA (Figure 3C). Figure 3D shows a timeline and the actual mRNAseq counts for the spotted gar, zebrafish, and medaka. Note the relatively high levels of expression of *adcy9a* in the brain for the teleost species, and the very low levels in the heart.

### Distinct features of cAMP production by the AC9 paralogs of zebrafish

**Figure 4:**
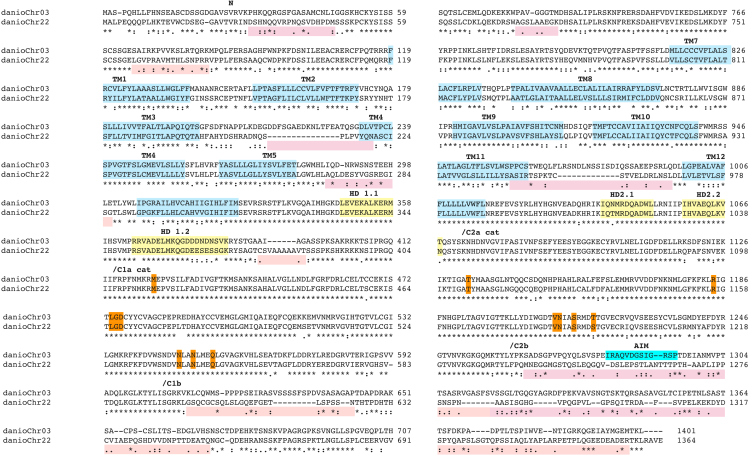
Alignment of the primary structures of the AC9 paralogs of zebrafish N—N terminal cytoplasmic domain, TM - transmembrane helices (light blue), HD1.1, 1.2, 2.1, 2.2 - alpha-helical domains in the signaling helix (yellow). Areas of significant sequence variation are highlighted in pink. The residues in C1a and C2a, highlighted in orange, interact with the ARM (highlighted in cyan) when it occludes the active site of the bovine AC9.

In the case of the zebrafish, *adcy9a* is on chromosome 3, and *adcy9b* is on chromosome 22. The zebrafish *adcy9a* has not been unambiguously identified by exon prediction methods (NCBI or Ensembl servers). However, a full-length transcript (FDR_LOC101477119.1.1) is found in the PhyloFish dataset (http://phylofish.sigenae.org/index.html), and all ten exons could be identified in the chromosome 3 assembly (reverse strand, position 9739058 to 12113782) of the Ensembl server. The cDNAs of both zebrafish AC9 isoforms were cloned from first-strand cDNA prepared from 5 d.p.f. embryos. The aligned primary sequences are shown in Figure 4. It is clear that these isoforms are similar, sharing the “low forskolin response” configuration of vertebrate AC9 orthologs. The catalytic domains are nearly identical. The post-TGD specific modification W1158G is present in both paralogs, and AC9a also features the common post-TGD modification V1264A of the ARM. As for other AC9b orthologs, a double leucine motif in the N-terminal domain L9L10, that is reportedly responsible for receptor-induced internalization of mammalian AC9,^21^ is absent from zebrafish AC9b, but present in AC9a.

**Figure 5:**
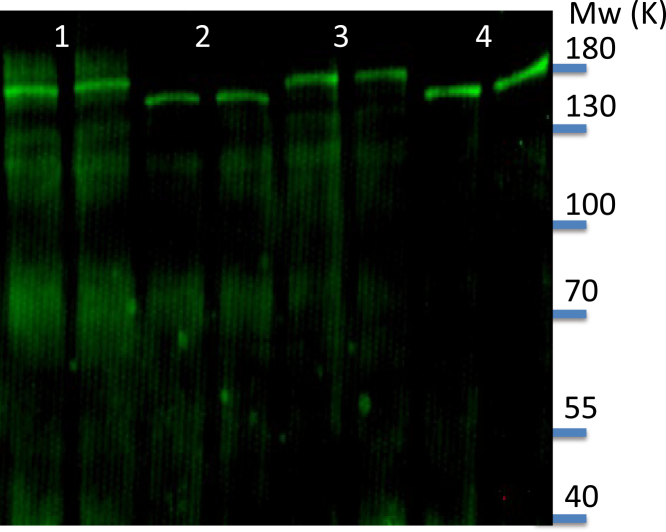
Heterologous expression of the zebrafish GFP-AC9 paralogs in HEK293FT cells western blot of crude membrane extracts reacted with anti-GFP serum. Samples (in duplicate) are: eGFP-AC9a (1), eGFP-AC9a_C2a (2), eGFP-AC9b (3), and eGFP-AC9b_C2a (4) Note the lower apparent molecular weight of the C2b truncated versions AC9a_C2a and AC9b_C2a.

Both AC9 cDNAs were transiently expressed with eGFP fused in frame to the N-terminus in HEK293FT cells. Western blots of membranes prepared from transiently transfected HEK293FT cells showed similar levels of expression for all AC9 proteins (Figure 5).

**Figure 6:**
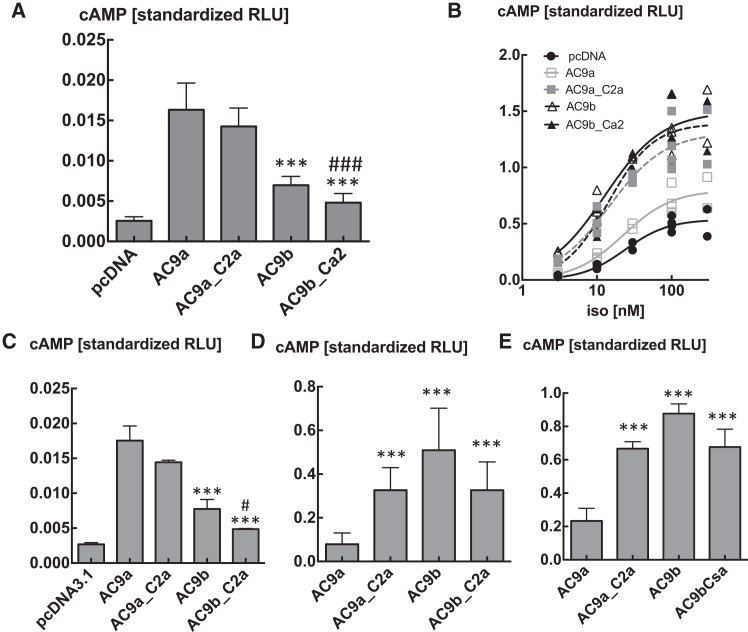
Distinct properties of zebrafish AC9a and AC9b, heterologously expressed in HEK293FT cells AC9a_C2a and AC9b_C2a lack the C2b domain of the respective AC9a and AC9b proteins. (A) Intracellular basal levels of cAMP, means ± SD, *n* = 12/group, representative of three independent experiments with similar results. ∗∗∗*p* < 0.001 different from respective group of AC9b, ++*p* < 0.01, different from AC9b_C2a. One-way ANOVA followed by Tukey’s multiple comparison test. (B) Isoproterenol evoked intracellular cAMP levels; all data points are shown. Representative of three independent experiments with similar results. The grand averages of three independent experiments C) Basal cAMP levels, D) cAMP responses to 10 nM, and E) 100 nM isoproterenol. Means ± SD, *n* = 3/group. ∗∗∗*p* < 0.001 different from AC9 #*p* < 0.05 vs. AC9b. One-way ANOVA followed by Tukey’s multiple comparison test.

Intracellular cAMP levels were monitored by the detection of light emitted by co-transfected glosensor22F. Comparison of AC9a and AC9b showed that the former produced significantly higher basal levels of cAMP-dependent light emission (Figure 6A). Activation of beta(2)-adrenergic receptors endogenously expressed by HEK293FT cells^22^ by isoproterenol (Iso) produced concentration-dependent increases of cAMP. Notably, the Iso-induced cAMP response produced by AC9a was not significantly different from that of the groups expressing the backbone vector pcDNA3.1 (Figure 6B). This is similar to previous findings with human AC9.^5^^,^^8^ In contrast, a robust rise of intracellular cAMP in response to Iso which was well above the host cell response, occurred in the cells expressing AC9b (Figures 6B–6D). These findings strongly indicated autoinhibition by the C2b domain through ARM in AC9a as previously observed for human and bovine AC9.^5^^,^^6^^,^^8^ Accordingly, when the C2b domain was removed from AC9a, the response to Iso was not different from that observed with AC9b. In contrast, removal of the C2b domain slightly diminished the Iso response of AC9b (Figures 6B–6D).

A yet further aspect of the ARM in human AC9 is autostimulation.^8^ This is likely due to the interaction of the first six N-terminal amino acid residues of ARM with the forskolin binding-pocket of AC9.^6^^,^^7^ However, the removal of C2b had only a relatively minor effect on the high basal levels of cAMP produced by AC9a, while the reduction for AC9b was more substantial (basal cAMP levels generated by AC9a_C2a were 80 ± 9% of those generated by AC9a, *n* = 3 independent experiments; for AC9b vs. AC9b_C2a, this value was 45 ± 9%, *n* = 4 independent experiments, data are means ± SD, *p* < 0.01 student’s *t* test). In the case of AC9b, the greater reduction could be due to its relatively low basal activity in HEK293FT cells that is close to the instrument background.

## Discussion

We found that a long C2b domain in *adcy9* is uniformly present and specific to vertebrate species. Gene duplication at TGD resulted in functionally distinct *adcy9* ohnologs that persisted in the teleost genome. The key trait that differentiates the sub-functionalized AC9 genes is autoinhibition of receptor-stimulated cAMP production by the C2b domain. The respective expression patterns of the two AC9 mRNAs in post-TGD species indicate that autoinhibition is fundamentally important for brain function.

The amino acid compositions of the C2b domains of the human as well as the two zebrafish AC9 isoforms clearly suggest intrinsic disorder (Figure S1). This conforms to the cryo-EM studies of the bovine and human orthologs that failed to assign any structural features to the C2b domain of the protein.^6^^,^^7^ Intrinsically disordered C-termini in proteins are frequently associated with autoinhibition.^23^^,^^24^ Indeed, a highly conserved and functionally relevant ARM is invariably found in the C2b domain of vertebrate AC9a orthologs and appears to exert a profound autoinhibitory effect on receptor-stimulated cAMP production.^5^^,^^6^^,^^8^ There are various potential mechanisms of genome modification by which the C-terminal extension of *adcy9*, a gene that is already discernible in *C. elegans*,^3^ may have occurred.^25^ Alternative exon splicing, recruitment of non-coding DNA, seems unlikely as no significant similarity in the existing databases could be found outside the *adcy9* genes of vertebrates. Transposon-mediated transfer^26^ or viral sequence domestication^27^ seem more plausible but require further investigation.

Importantly, the long C2b tail of *adcy9* is present in cyclostomes that are the most ancient vertebrates alive today and already have a regionally differentiated brain.^28^ Indeed, similarly to rodent and human brain^29^^,^^30^^,^^31^ *adcy9* mRNA is widely expressed in lamprey brain see https://lampreybrain.kaessmannlab.org/.^32^ When compared to the expression in the brain, only low levels of *adcy9* mRNA are found in the peripheral tissues of post-TGD teleosts, including zebrafish. Intriguingly, the coding sequence of ohnolog adcy9b has no ARM, and the expressed protein showed no autoinhibitory activity with respect to receptor-stimulated cAMP production. Based on mRNA expression results in the PhyloFish database, the *adcy9b* gene has largely replaced *adcy9a* in all tissues except the brain. Proteomic analysis reported the presence of AC9a in the synaptosomal fraction of adult zebrafish brain.^33^ Taken together with the functional analysis detailed in the present study, the data point to the sub functionalization of the *adcy9* ohnologs in post-TGD teleosts.

Overall, little is known about the biological role of the *adcy9* gene. In rodents, the deletion of *adcy9* causes marked (≈90%) pre-weaning lethality of the offspring homozygous for the deletion.^34^^,^^35^ The adult survivors show relatively minor defects of heart function and normal levels of anxiety.^34^^,^^36^ In zebrafish, morpholino-based knockdown of *adcy9b* produced 50% mortality of embryos at birth and severe heart failure at 3 d.p.f.^37^ Provided the morpholino effect was selective,^38^ the results support the findings derived from the PhyloFish database as well as from real-time PCR in the present study, that *adcy9b* expression predominates in the heart. In this context, it is of interest to note that a lower molecular weight AC9 that lacks the C2b domain is the dominant AC9 protein in the rodent heart and is also readily detectable in human heart tissue samples.^5^^,^^39^ Similar to zebrafish AC9b, the truncated form of human AC9 has low basal activity and responds robustly to activation by Gs-coupled receptors.^5^^,^^8^ This indicates that the autoinhibition of AC9 is not advantageous for heart function in zebrafish or mammals. As autoinhibition increases the Km for ATP by 40-fold,^6^ it seems possible that cAMP production by AC9 may be impaired at low levels of ATP^40^ that occur during the heart cycle. In turn, this would be detrimental for the pumping action of the heart.^41^ A further indication that autoinhibition in the single mammalian *adcy9* gene is an evolutionary burden in some tissues is that the ARM is subject to dynamic phosphorylation at Ser1269 as well as Ser1273.^42^^,^^43^ Indeed, alanine substitution of Ser1273 attenuated the autoinhibitory effect in HEK293 cells.^5^

A marked auto stimulatory activity of C2b is readily demonstrated in human AC9.^8^ In contrast, removal of the C2b domain from zebrafish AC9a had only a modest effect on basal cAMP levels. A possible explanation of this is that the zebrafish enzyme was tested in a human cell line, and the level of auto stimulatory activity may depend on a species-specific cell context. Furthermore, zebrafish AC9a has two notable amino acid changes when compared to its mammalian as well as its pre-TGD orthologs. First, V1266A of the highly conserved ARM is likely to weaken the interaction of ARM with the forskolin binding-pocket that is a positive allosteric modulator of AC9.^6^^,^^18^ Second, W1160G is located within a short AC9-specific motif of the catalytic C2a domain that interacts with Gsα in the bovine enzyme.^6^^,^^18^ Thus, it seems possible that these two mutations in zebrafish AC9a, which are shared by the vast majority of the post-TGD *adcy9* orthologs currently in the databases, diminished the auto stimulatory activity of C2b. The AC9b of zebrafish consistently produced lower basal cAMP levels than AC9a. As these were at best 3-fold above the basal cAMP levels generated by the endogenous ACs of the host cell, the 50% reduction of basal levels observed upon removing the C2b domain translates into a marked, 75–80% reduction of basal cAMP production when taking into account the output of the host cells. It is tempting to suggest that this is as yet a further aspect of sub functionalization of the *adcy9* genes i.e., the segregation of auto stimulation to *adcy9b* and autoinhibition to *adcy9a*.

In summary, TGD resulted in sub-functionalized *adcy9* ohnologs in teleosts to overcome an adaptive challenge met by post-translational modifications of the AC9 protein in mammals.^44^ On average, over 80% of ohnolog pairs revert to a single gene after TGD; thus, the persistence of the *adcy9* ohnologs for over 200 million years indicates a significant adaptive advantage.^15^^,^^16^^,^^45^ The high expression of *adcy9a* in the brain and its restricted expression in peripheral tissues argue that the autoinhibitory feature is essential for some aspect of brain function or development. However, autoinhibition appears to be an evolutionary burden in peripheral tissues, particularly the heart. Hence, our results also illustrate the power of whole genome duplication to eliminate evolutionary burden.

### Limitations of the study

The regulatory properties of the AC9 ohnologs were examined in a relatively primitive assay system. Monitoring of intracellular cAMP *in vivo*, with high spatial and temporal resolution, could shed light on further differences between the ohnologs.

The origins and the temporal sequence of appearance of the main mutations of AC9 that underlie its unique regulatory properties in vertebrates are unknown. It is also unknown whether or not any of the parameters analyzed depend on sex.

## Resource availability

### Lead contact

Requests for further information and resources should be directed to and will be fulfilled by the lead contact, Ferenc Antoni, ferenc.antoni@ed.ac.uk; franzantoni@gmail.com.

### Materials availability

AC9a and AC9b expression vectors are available on request from the corresponding authors.

### Data and code availability

• The results of all database searches are shown in the supplement.
• Excel files of the biosensor recordings reporting cAMP levels are attached as Table S2.
• The western blot image shown is uncropped except for the position of the size markers.
• The coding sequence of zebrafish *adcy9a* has been registered as adenylyl_cyclase_9a GenBank: PX511566.
• This paper does not report original code.
• Any additional information required to reanalyze the data reported in this paper is available from the lead contact upon request.

## Acknowledgments

We thank Dr. Mark O. Collins, University of Sheffield, for the reanalysis of proteomic data.^33^ This work was funded by the University of Edinburgh and MRC, UK grant nos. MR/V012290/1 and MR/R010668 to M.J.S.

## Author contributions

F.A.A.: concept, design, data acquisition and analysis, and wrote the paper; J.M.: design, data acquisition and analysis, and wrote the paper; H.M.: design, data acquisition and analysis, and wrote the paper; Z.C.: design and data acquisition and analysis; L.Sz. design and data acquisition and analysis; C.X.: design and data acquisition and analysis; S.I.: design and data acquisition and analysis; M.D.: concept, design, data analysis and wrote the paper; A.B.: concept, design, data analysis, and wrote the paper; D.S.: concept, design, data analysis, and wrote the paper; M.J.S.: concept, data analysis, and wrote the paper; P.S.: design, data acquisition and analysis, and wrote the paper.

## Declaration of interests

The authors declare no competing interests.

## STAR★Methods

### Key resources table

**Table:** 

REAGENT or RESOURCE	SOURCE	IDENTIFIER
**Antibodies**		

Anti GFP	Takara Bio	Cat#632592
Alexa 680 anti-rabbit	Invitrogen	Cat#A32734
Anti-4C4 (mouse monoclonal)	Sieger Laboratory, University of Edinburgh	

**Biological samples**		

cDNA from 5 dpf zebrafish microglia	Sieger Laboratory, University of Edinburgh	
cDNA from 8 dpf zebrafish neuronal cells	Sieger Laboratory, University of Edinburgh	
cDNA from 5 dpf zebrafish larvae	Sieger Laboratory, University of Edinburgh	

**Chemicals, peptides, and recombinant proteins**		

IBMX	Merck	Cat#I5879
isoproterenol	Merck	Cat#I5627
luciferin	Promega	Cat#E1603

**Critical commercial assays**		

Qiagen RNeasy Plus Micro kit	Qiagen	Cat#74134
Ovation RNA-Seq System V2 kit	NuGen	Cat#7102
ReliaPrep™ RNA Tissue Miniprep System	Promega	Cat#Z6111
SuperScript™ IV First-Strand Synthesis System	ThermoFisher	Cat#18091050
Power SYBR™ Green PCR Master Mix	Applied Biosystems	Cat#4367659
InFusion cloning kit	Takara Bio	Cat#638946
PrimePCR™ SYBR® Green Assay actb2, Zebrafish,	BIORAD	Cat#12001961
PrimePCR™ SYBR® Green Assay: eef1a1|1, Zebrafish	BIORAD	Cat#10x′041596

**Deposited data**		

adenylyl_cyclase_9a	GenBank	PX511566

**Experimental models: Cell lines**		

HEK293 FT cells	Invitrogen	Cat#R70007

**Experimental models: Organisms/strains**		

Et(zic4:GAL4TA4,UAS:mCherry)hmz	Distel et al.^46^	RRID:ZFIN: ZDB-ETCONSTRCT-110214-1
Tg(NBT:dsRed)	Peri and Nüsslein-Volhard^47^	ZDB-PUB-180515-7

**Oligonucleotides**		

Chr3Fwd 5′ATGGCTTCTCCTCAGCATCTAC3′	Invitrogen	
Chr3Rev 5′TCAAAGTTTTGTCATTTCACCCA3′	Invitrogen	
Chr22Fwd 5′ATGGCATTGCCAGAGCAACAGC3′	Invitrogen	
Chr22Rev 5′TCACTCCACTGCCCTGAGCTTTG3′	Invitrogen	
Chr3 sense: TGTGAGTTCAGGACTTAC	Sigma_Aldrich	
Chr3 antisense: TTAGATGTACCATTGCTTAC	Sigma_Aldrich	
Chr22 sense: TACACAACCTTCCTCAAT	Sigma_Aldrich	
Chr22 antisense: TCTGCTTCATCTTCTTCT	Sigma_Aldrich	

**Recombinant DNA**		

Glosensor22F	Promega	Cat#E2301
pEGFP_C2	TakaraBio	discontinued

**Software and algorithms**		

BLAST	www.ncbi.nlm.nih.gov	
Clustal Omega	www.ebi.ac.uk	
Cobalt	www.ncbi.nlm.nih.gov	

**Other**		

Phylofish database	www.sigenae.org	
Ensemble	www.ensembl.org	
GenBank	www.ncbi.nlm.nih.gov	

### Experimental model and study participant details

#### Zebrafish care and husbandry, ethical approval

All experiments were carried out in accordance with the accepted standards of humane animal care under the regulation of the UK Animal (Scientific Procedures) Act 1986 and EU Directive 2010/63/EU; all experiments were approved by the University of Edinburgh Animal Welfare and Ethical Review Board.

Adult wild-type fish were housed according to standard operating procedures (https://zfin.org/zf_info/zfbook/zfbk.html,^48^) and maintained with a 14h light:10h darkness photoperiod cycle at an ambient water temperature of 28.5°C. The established line used was WIK. Adult zebrafish were housed in 10L tanks at a density of 2–3 fish per liter (maximum of 30 fish per tank).

All larvae used in these experimental studies were under the age of 5 days post fertilisation; embryos were collected from random matings and then correctly staged for development, according to.^49^ The larvae were kept at 28°C on a 14 h light/10 h dark photoperiod. Embryos were obtained by natural spawning from adult *tg(mpeg1:GFP*)^50^ and wild-type (WIK) zebrafish strains. Embryos were raised at 28.5°C in embryo medium (E3) and treated with 200 μM 1-phenyl 2-thiourea (PTU) (Sigma) from the end of the first day of development for the duration of the experiment to prevent pigmentation.

#### Macrophage isolation and FACS analysis

Macrophages were isolated by FACS from heads of mpeg1:GFP 5 days post fertilization (d.p.f.) larvae as previously described.^51^ FACS allowed cell separation from debris in function of their size (FSC-A) and granularity (SSC-A). Single cells were then separated from doublets or cell agglomerates (FSC Singlet; SSC Singlet). From the single-cell population, a gate was drawn to separate live cells (DAPI−) from dead cells (DAPI+). Unstained cells from wild-type (WIK) zebrafish strain were used as controls to draw gates corresponding to macrophage (GFP^+^) populations segregated from the live cell population gates. FACS data were analyzed using FlowJo Software (Treestar, Ashland, OR).

#### Human embryonic kidney 293 FT cells (HEK293FT)

The cells were originally supplied by Invitrogen (Cat#R70007). The cells were maintained and propagated (including frozen stocks) in Dulbecco’s MEM containing 4.5 g/L D-glucose, 1 mM sodium pyruvate, 4 mM Glutamax and 10% (v/v) fetal bovine serum in house, as previously reported.^5^^,^^29^ Experiments were carried out on cells up to 10 passages from thawing. Mycoplasma levels were not monitored.

### Method details

#### Database searches

The databases and software packages provided by NCBI (https://www.ncbi.nlm.nih.gov), European Bioinformatics Institute (https://www.ebi.ac.uk), ENSEMBL (https://www.ensembl.org/index.html), PhyloFish (http://phylofish.sigenae.org/index.html) and Zebrafish information network (https://zfin.org/) were used to collate genomic data and carry out sequence alignments. With respect to sequence alignments, all of the sequences used were designated as adenylyl cyclase 9-like by the database curators on the basis of BLAST max scores and had error rates (E) of 0 ≤ E < 10^−50^. Protein sequence alignment and phylogenetic tree construction was carried out with COBALT (https://www.ncbi.nlm.nih.gov/tools/cobalt/cobalt.cgi).

#### RNA extraction and cDNA amplification

Total RNA extraction from FACS derived macrophages was performed using the Qiagen RNeasy Plus Micro kit according to the manufacturer’s guidance (Qiagen). For qPCR, RNA sample quality and concentration were assessed using the LabChip GX Touch Nucleic Acid Analyzer and RNA Pico Sensitivity Assay. All RNA samples with a RIN score >7 were transcribed from the same amount of RNA into cDNA using the Super-Script III First-Strand Synthesis System (Invitrogen).

#### Quantitative PCR for *adcy9* expression in zebrafish

Tissues from adult or 5 d.p.f. zebrafish (Wik) were excised after an overdose of MS-222 (tricaine methanesulfonate - 40 μg mL^−1^, Sigma), and placed in “RNA later” (Ambion, Life technologies) solution. The RNA was extracted with ReliaPrep RNA Miniprep kit (Promega) as per the manufacturer’s protocol and reverse transcribed using SuperScript IV First-Strand Synthesis System (ThermoFisher). Approximately 100-700 ng RNA was primed with a 1:1 mix of oligo-dT and random hexamers and relative gene expression was determined by comparative qPCR using Power SYBR Green PCR Master Mix (Applied Biosystems) on a StepOnePlus Real-Time PCR System. Primers for the zebrafish *adcy9* coding sequences were designed by Oligo Tech Service (Sigma/DORSET/UK). The *adcy9* genes of zebrafish are found on chromosome 3 and 22.

#### Chr3 sense: TGTGAGTTCAGGACTTAC

Chr3 antisense: TTAGATGTACCATTGCTTAC 95.9% efficiency on zebrafish brain over 3 dilution factors. Chr22 sense: TACACAACCTTCCTCAAT, Chr22 antisense: TCTGCTTCATCTTCTTCT 94.2% efficient on total zebrafish cDNA over 3 dilution factors. The house-keeping genes *bactin2* and *eef1* were used as the ref.^52^

#### Partial characterization of zebrafish AC9 isoforms

The full length cDNAs of the zebrafish AC9s could be amplified from cDNA prepared from whole 5 d.p.f. larvae with Phusion polymerase (NEB, Hitchin,UK) using primers

**Table:** 

Chr3Fwd 5′ATGGCTTCTCCTCAGCATCTAC3′
Chr3Rev 5′TCAAAGTTTTGTCATTTCACCCA3′
Chr22Fwd 5′ATGGCATTGCCAGAGCAACAGC3′
Chr22Rev 5′TCACTCCACTGCCCTGAGCTTTG3′

These primers were subsequently extended by the requisite 15 bases and used to amplify the full length AC9 by Phusion PCR for insertion into pEGFP_C2 (TakaraBio) by Gibson assembly^53^ as implemented in the TakaraBio InFusion cloning kit. The carboxyl-terminal C2b domains of both AC9s were removed by PCR with the Phusion polymerase/Infusion procedure resulting in AC9a_C2a and AC9b_C2a. All AC9 cDNA plasmids were verified by restriction digests and Big Dye or nanopore DNA sequencing.

Intracellular levels of cAMP were monitored by light emission from the cAMP biosensor protein Glosensor22F^54^ as described previously.^8^ Briefly, HEK293FT were transfected in suspension (10^6^ cells/ml) with up to 1.33 μg plasmid of interest plus 0.67 μg of Glosensor 22F DNA, with 1.6 μL of Lipofectamine 2000 (Life Technologies) reagent per mL of cell suspension in 6 cm diameter culture dishes (Greiner). Forty-eight h after the start of the transfection the cells were replated at 10^5^ cells/well in poly-L-lysine coated 96-well white tissue-culture plates (Greiner). After 24h the culture medium was changed to a serum free mixture of (1:1) Dulbecco’s MEM and Ham’s F12 medium and the cells were incubated in 5% CO2 atmosphere at 37°C for 1h. Subsequently, the medium was changed to Hank’s balanced salt solution containing 1 mM MgCl_2_, 1.5 mM CaCl_2_, 10 mM HEPES pH7.4, 1 mM luciferin and the plates were transferred to a plate warmer set to 32°C. After 60 min the recording of bioluminescence was started in a Lumistar Omega (BMG Labtech) plate reader at 32°C. Basal levels were recorded for 30 min after which isoproterenol was added by a hand-held multichannel pipette. The response to isoproterenol was monitored for 30 min after which forskolin 5μM/IBMX 0.5 mM was added and the recording terminated 30 min afterward. The cAMP levels elicited by the forskolin 5μM/IBMX 0.5 mM mixture plateaued after 5 min and the average plateau relative light unit (RLU) value was used to standardize the cAMP production in each well to obviate the inherent variability of transient transfections. This standardization is possible because AC9 is not stimulated by 5 **μ**M forskolin even when stimulated by Gs**α,** and there is a strong synergy between Iso and forskolin to stimulate the HEK293 cells’ endogenous adenylyl cyclases.^8^

#### Immunoblots

HEK 293 cells transiently expressing the zebrafish eGFP-AC9 conjugates were processed for immunoblots as previously reported^5^ except that whole cell extracts were analyzed. The blots were reacted with rabbit antibodies against GFP (TakaraBio), followed by anti-rabbit IgG Fab fragments conjugated to Alexa 680 dye (Invitrogen, UK), and subsequently scanned in a LiCor Odyssey fluorescence imager.

### Quantification and statistical analysis

The standardized RLU data were analyzed after log-transformation by one-way ANOVA followed by Tukey’s multiple comparisons test as implemented in Graphpad Prism, v.6.0.

#### Additional resources

BLAST www.ncbi.nlm.nih.gov, Clustal Omega www.ebi.ac.uk, Cobalt www.ncbi.nlm.nih.gov, Phylofish database, www.sigenae.org, Ensemble www.ensembl.org, GenBank www.ncbi.nlm.nih.gov.

## Supplemental information

Document S1. Figures S1–S3

Table S1. List of AC9 sequences used to construct Supplementary Figure 2

Table S2. Raw data of intracellular cAMP monitoring with glosensor 22F

Data S1. Alignments of AC9 protein primary sequences of various species relevant to teleost specific genome duplication
