# Supplementary material for: Adenylyl cyclase 9: Fundamental change of regulation in vertebrates and gene sub-functionalization after teleost-specific whole-genome duplication
Source: iScience. 2026 Jul 7;29(7):116562. doi: 10.1016/j.isci.2026.116562 (PMC13356687; doi:10.1016/j.isci.2026.116562)
Supplement: Document S1. Figures S1–S3 [file mmc1.pdf]

iScience, Volume *29*

Supplemental information

Adenylyl cyclase 9: Fundamental change of regulation in vertebrates and gene sub-functionalization after teleost-specific whole-genome duplication

Ferenc A. Antoni, Julie Mazzolini, Heather McClafferty, Zhiaho Chen, Laura Szalai, Cristina Xia, Sahad Iqbal, Martin Denvir, András Balla, Dirk Sieger, Michael J. Shipston, and Paul Skehel

Supplementary figures

Legends:

Suppl. Fig. 1

Amino acid composition of the C2b domain of human AC9 expressed as relative abundance of the residues when compared with the average abundance of the respective residue in the SwissProtein database

https://www.uniprot.org/uniprotkb/statistics#amino-acid-composition. (Related to Figure1)

A value above zero indicates higher relative abundance than the Swiss Protein average relative abundance. Note that four of the five the most abundant residues in the C2b domain (bracket) are known for their high disorder promoting properties.

Suppl. Fig. 2 Phylogenetic tree of the adenylyl cyclase 9 protein sequences of selected species downloaded from Genbank or Ensemble servers (20 Nov 2025) and generated upon sequence alignment in COBALT. (Related to Figure 3).

The following sequences were used and recoded for clarity in the alignment. Sequences containing the ARM are indexed as 3 and those not containing it as 22, i.e. similar to zebrafish AC9a (chromosome 3) and AC9b (chromosome 22) respectively. The invertebrate species sequences and several vertebrate ones are predicted and annotated from genomic sequence by automated computational analysis at Genbank.

Suppl. Fig. 3 All the currently known hallmarks of autoregulation are apparent in the primary structures of the AC9 orthologues of jawless fish. (Related to Figure 4)

S. lamprey – sea lamprey, j. lamprey – Japanese lamprey

Suppl. Fig. 1

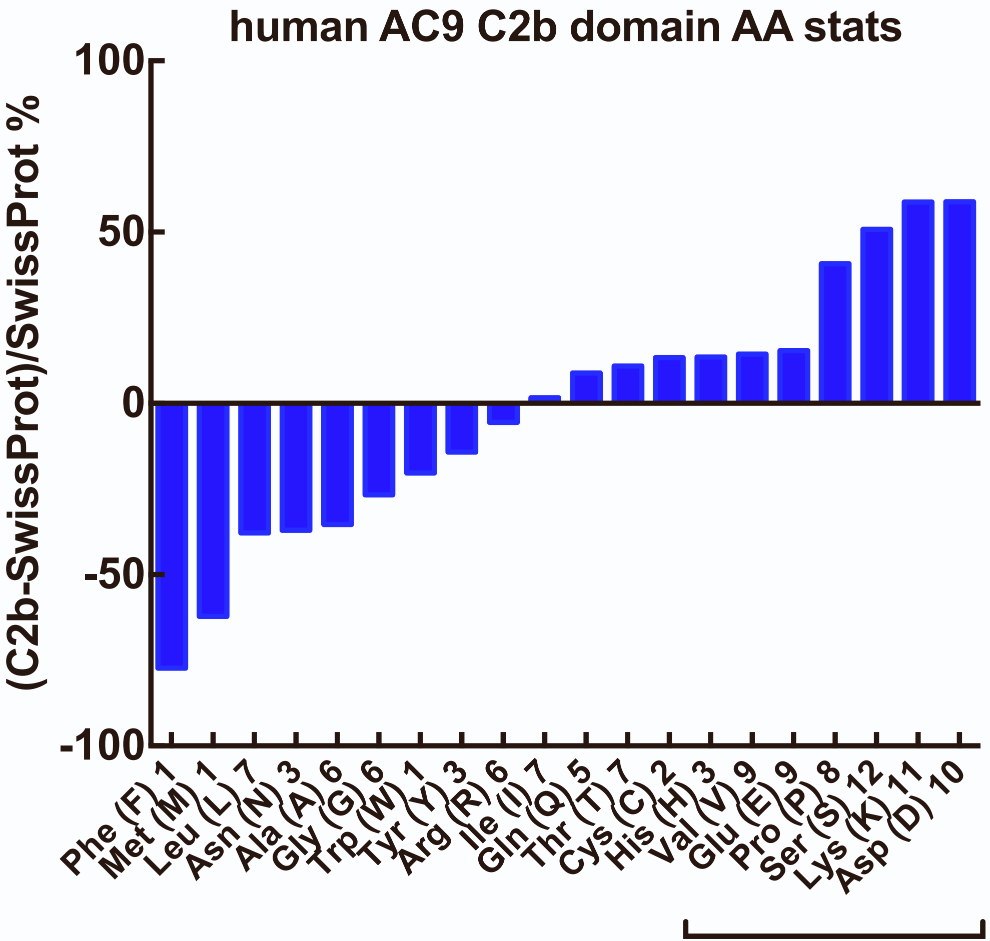

human AC9 C2b domain AA stats
(C2b-SwissProt)/SwissProt %
100
50
0
-50
-100
Phe (F) 1
Met (M) 1
Leu (L) 7
Asn (N) 3
Ala (A) 6
Gly (G) 6
Trp (W) 1
Tyr (Y) 3
Arg (R) 6
Ile (I) 7
Gln (Q) 5
Thr (T) 7
Cys (C) 2
His (H) 3
Val (V) 9
Glu (E) 9
Pro (P) 8
Ser (S) 12
Lys (K) 11
Asp (D) 10

Suppl. Fig. H

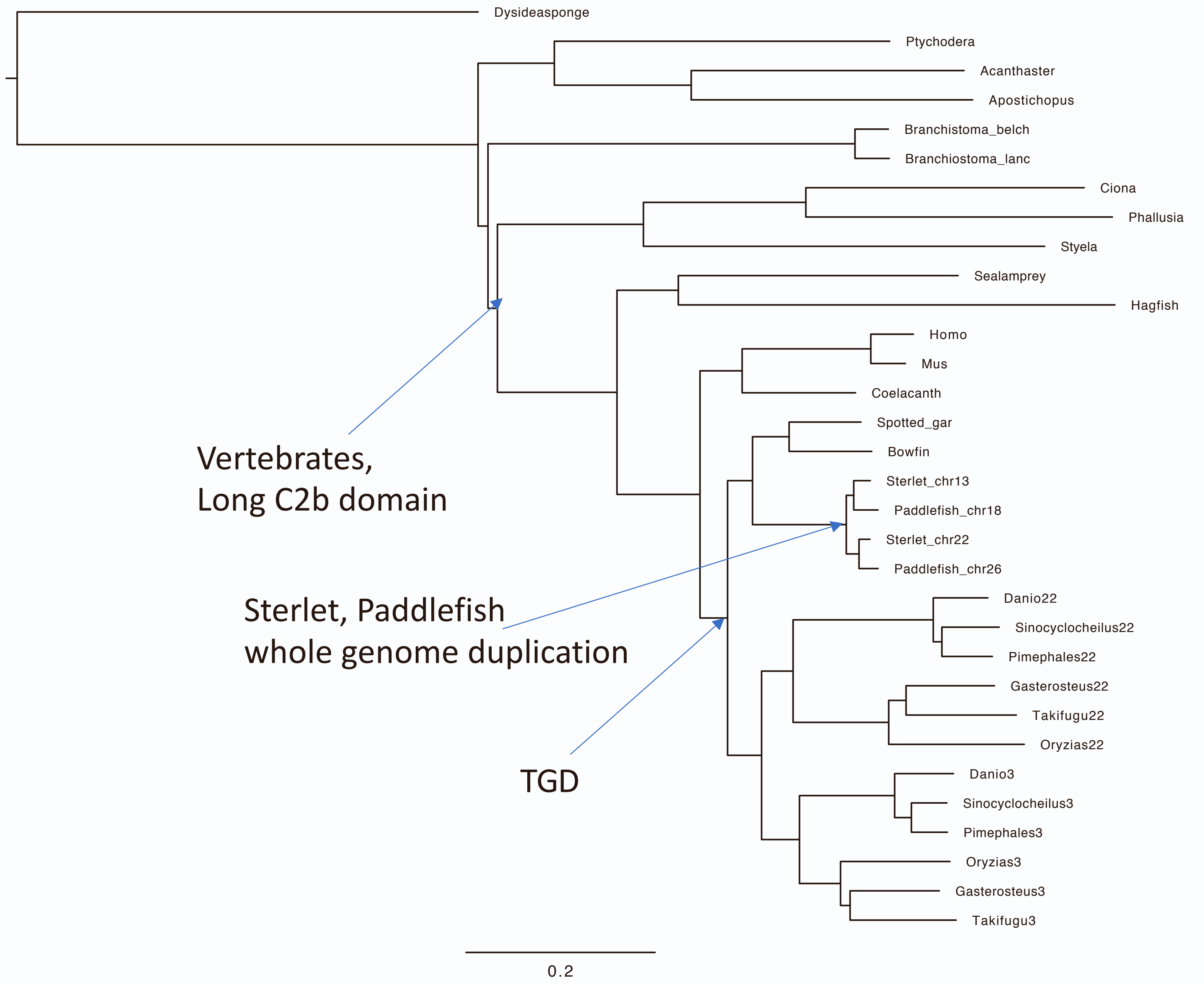

Dysideasponge
Ptychodera
Acanthaster
Apostichopus
Branchistoma_belch
Branchiostoma_lanc
Ciona
Phallusia
Styela
Sealamprey
Hagfish
Homo
Mus
Coelacanth
Spotted_gar
Bowfin
Sterlet_chr13
Paddlefish_chr18
Sterlet_chr22
Paddlefish_chr26
Danio22
Sinocyclocheilus22
Pimephales22
Gasterosteus22
Takifugu22
Oryzias22
Danio3
Sinocyclocheilus3
Pimephales3
Oryzias3
Gasterosteus3
Takifugu3
Vertebrates,
Long C2b domain
Sterlet, Paddlefish
whole genome duplication
TGD
0.2

Suppl.Fig.0

```
...
human           GTVLCGILGMRRFKFDVWSNDVNLANLMEQLGVAGKVHISEATAKYLD-DRYEMEDGKVI    552
hagfish         GTVLCGILGMKRFKFDVLSNDVNLANMMEQLGVAGKVHVSEATANCITDDRYLREDGLLS    462
s.lamprey       GTVLCGILGMKRFKFDVLSNDVNLANMMEQLGVAGKVHVSEATALCLD-ERYTMEDGRLF    557
j.lamprey       GTVLCGILGMKRFKFDVLSNDVNLANMMEQLGVAGKVHVSEATALCLD-ERYTMEDGRLF    631
                **********:****** ********:***********:*****  :  :**  *** :

...
human           GVIFASIVNFSEFYEENYEGGKECYRVLNELIGDFDELLSKPDYSSIEKIKTIGATYMAA    1118
hagfish         GVIFASIVNFREFYEEKFEGGKECYRVLHELMSDLDQLLSHSDFTCIEKIKTIGATYMAA    1057
s.lamprey       GVIFASIVNFSEFYEENYEGGKECYRVLHELMSDLDQLLARDHYGGIEKIKTIGATYMAA    1195
j.lamprey       GVIFASIVNFSEFYEENYEGGKECYRVLHELMSDLDQLLAREHYGGIEKIKTIGATYMAA    1077
                ********** *****::**********:**:.*:*:**:: .:  **************

...

human           DFDYRGTVNVKGKGQMKTYLYPKCTDHRVIP--------QHQLSISPDIRVQVDGSIGRS    1274
hagfish         RFEYRGTVNVKGKGQMKTYLYPCKDDAPS----------QPTLPVTNVYHAQVDGSIGRS    1227
s.lamprey       QFDYRGTVNVKGKGQMKTYLFPPTDGGHAAPQPPPPPLLPPLLPMTPDIHTQVDGSIGRS    1358
j.lamprey       QFDYRGTVNVKGKGQMKTYLFPPTDGGHAAPQPPPPP-LPPLLPMTPDIHTQVDGSIGRS    1239
                 *:*****************:*   .                *  ::   :.*********

human           PTDEIANLVPSVQYVDKTSLGSDSSTQAKD---AHLSPKRPWKEPVKAEERGRFGKAI--    1329
hagfish         PADENGEIASTSTQLASDKLVDN-------SELSGSEPSQNLSDGVTAPDASSTSeaDL-    1279
s.lamprey       PAEEEAHSLLASLSASDACIITFSESPPRGGGGGGDSPRRALA-GVRGQRASSLSSLPHG    1417
j.lamprey       PAEEEAHSLLASLSASDACIITFSESPPRGGGGGGDSPRRALAGGVRGQRASSLSSLPHG    1299
                *::* ..   :     .  :             .  .* :     * .   .  ..
```
