# Supplementary material for: Adenylyl cyclase 9: Fundamental change of regulation in vertebrates and gene sub-functionalization after teleost-specific whole-genome duplication
Source: iScience. 2026 Jul 7;29(7):116562. doi: 10.1016/j.isci.2026.116562 (PMC13356687; doi:10.1016/j.isci.2026.116562)
Supplement: Table S1. List of AC9 sequences used to construct Supplementary Figure 2 [file mmc2.pdf]

Supplementary Table 1
List of *adcy9* entries used to construct Supplementary Figure 2

Acanthaster	*Acanthaster planci* adenylate cyclase type 9-like (LOC110976938), transcript variant X4, mRNA
Apostichopus	Apostichopus japonicus Acanthaster planci adenylate cyclase type 9-like (LOC110976938), transcript variant X4, mRNA
Bowfin	Amia ocellicauda adenylate cyclase 9a (adcy9a), transcript variant X1, mRNA XM_066689961.1
Branchiostoma_belch	Branchiostoma belcheri adenylate cyclase type 9-like (LOC109470759), mRNA
Branchistoma_lanc	Branchiostoma lanceolatum adenylate cyclase type 9-like (LOC136437063), transcript variant X1, mRNA
Ciona	*Ciona intestinalis* adenylate cyclase type 9-like (LOC100176999)
Coelacanth	*Latimeria chalumnae* adenylate cyclase 9 (ADCY9), transcript variant X2, mRNA XM_014485369.2
Danio22	*Danio rerio* Adenylate cyclase 9 ENSDART00000157411.2 adcy9-201
Danio3	*Danio rerio* Adenylyl_cyclase_9a PX511566.
Dysidea	*Dysidea avara* adenylate cyclase type 9-like (LOC136239645), transcript variant X2, mRNA.
Gasterosteus22	*Gasterosteus aculeatus* adenylate cyclase 9 mRNA. XM_040186575
Gasterosteus3	*Gasterosteus aculeatus* adenylate cyclase type9 like (LOC120827674)
Hagfish	Eptatretus burgeri ENSEBUT00000028171.1 Contig FYBX02010769.1: 55,286-152,905 forward strand.
Homo	*Homo sapiens* Homo sapiens adenylate cyclase 9 (ADCY9), mRNA NCBI Reference Sequence: NM_001116.4
Mus	*Mus musculus* mRNA for adenylyl cyclase type 9 GenBank: Z50190.1
Oryzias22	*Oryzias latipes* adenylate cyclase type 9 (LOC101163149)
Oryzias3	*Oryzias latipes* adenylate cyclase 9 (adcy9), transcript variant X1, mRNA XM_011478180.3
Paddlefish_chr18	*Polyodon spathula* adenylate cyclase type 9-like (LOC121330489), transcript variant X1, mRNA
Paddlefish_chr26	*Polyodon spathula* adenylate cyclase type 9-like (LOC121300452), transcript variant X1, mRNA
Phallusia	*Phallusia mammillata* mRNA for adenylate cyclase type 9-like (LR782732.1)
Pimephales22	*Pimephales promelas* adenylate cyclase 9 (adcy9), mRNA XM_039694695.1
Pimephales3	*Pimephales promelas* adenylate cyclase type 9 (LOC120489506), transcript variant X1, mRNA
Ptychodera	*Ptychodera flava* adenylate cyclase type 9-like XP_070552629.1
Sealamprey	Petromyzon marinus adenylate cyclase 9 (ADCY9), transcript variant X1, mRNA XM_032970096.2
Sinocyclocheilus22	Sinocyclocheilus rhinocerous adenylate cyclase type 9-like (LOC107742090), transcript variant X2, mRNA
Sinocyclocheilus3	*Sinocyclocheilus rhinocerous* adenylate cyclase type 9-like (LOC107715643), mRNA
Spotted gar	*Lepisosteus oculatus* adenylate cyclase 9 (adcy9), transcript variant X1, mRNA
Sterlet_chr13	*Acipenser ruthenus* adenylate cyclase type 9 (LOC117418288), transcript variant X1, mRNA

Sterlet_chr22	*Acipenser ruthenus* adenylate cyclase type 9 (LOC117431652), transcript variant X1, mRNA
Styela	*Styela clava* adenylate cyclase type 9-like (LOC120340982), transcript variant X1, mRNA accession
Takifugu22	*Takifugu rubripes* adenylate cyclase type 9-like (LOC101074204)
Takifugu3	*Takifugu rubripes* adenylate cyclase type 9-like (LOC101078598)
