# Supplementary material for: Adenylyl cyclase 9: Fundamental change of regulation in vertebrates and gene sub-functionalization after teleost-specific whole-genome duplication
Source: iScience. 2026 Jul 7;29(7):116562. doi: 10.1016/j.isci.2026.116562 (PMC13356687; doi:10.1016/j.isci.2026.116562)
Supplement: Data S1. Alignments of AC9 protein primary sequences of various species relevant to teleost specific genome duplication [file mmc4.pdf]

Supplementary Data S1 legends

Data 1

Results of *blastp* search of the Genbank Core nucleotide database (20 May,2025) showing significant similarity with the C2b domain (M1237-V1353) of human adenylyl cyclase 9. (Related to Figure 2). The identities are shown with dots. The ARM (I1262-P1274) is highlighted in cyan. No significant homology was found in invertebrate species. Note high level of conservation of the ARM.

Data 2

Alignment of human AC9 with that of the spotted gar and bowfin, teleost species that have appeared before teleost specific genome duplication. (Related to Figures 3 and 4).

N – N terminal cytoplasmic domain, TM - transmembrane helices (light blue), HD1.1 , 1.2 , 2.1, 2.2 - alpha-helical domains in the signalling helix (yellow). Areas of significant sequence variation are highlighted in pink. The residues in C1a and C2a highlighted with orange form the binding pocket for the autoregulatory motif (ARM, highlighted in cyan) in bovine AC9. Note full conservation of the ARM and the marked divergence of the C2b domain after the ARM.

Data 3

Alignment of sterlet (*Acipenser ruthenus*) and paddlefish (*Polydon spathula*) predicted primary adenylyl cyclase 9 sequences. (Related to Figures 3 and 4). The main hallmarks of autoregulation are highlighted in cyan. The numbering of the highlighted residues are those of the human orthologue. The source of the sequences is Supplementary Table 1.

Query	1	MKTYLYPKCTDHRVIPQHQLSISPDIRVQVDGSIGRSPTDEIANLVPSVQYVDKTSLGSDSSTQAKDAHLSPKRPWKEPVKAEERGRFGKAIEKDDCDETGIEEANELTKLNVSKSV	117
XM_016929305.2	4010	............G..	4360
XM_008968014.3	4134	............G..	4484
XM_003815131.3	4239	............G..	4589
XM_016929306.2	4250	............G..	4600
XM_032144660.1	4231	............GL...R.......	4581
XM_032144661.1	4293	............GL...R.......	4643
XM_024233848.1	4094	............G...I..	4444
XM_024233847.1	4151	............G...I..	4501
XM_023225708.2	4176	............G.F..R...........................	4526
XM_023225707.2	4203	............G.F..R...........................	4553
XM_007984107.2	3951	............G.F....................................M.....................................R...........................	4301
XM_007984098.2	3952	............G.F....................................M.....................................R...........................	4302
XM_007984114.2	4241	............G.F....................................M.....................................R...........................	4591
XM_011982563.1	3784	............G.F..E.............................R...........................	4134
XM_015125415.2	3886	............G.F..E.............................R...........V...............	4236
XM_015125414.2	3902	............G.F..E.............................R...........V...............	4252
XM_028841144.1	3934	............G.F..E.............................R...........V...............	4284
XM_015125413.2	3943	............G.F..E.............................R...........V...............	4293
XM_011717619.2	4059	............G.F..E.............................R...........V...............	4409
XM_011717618.2	4084	............G.F..E.............................R...........V...............	4434
XM_011717616.2	4117	............G.F..E.............................R...........V...............	4467
XM_011717620.1	4236	............G.F..E.............................R...........V...............	4586
XM_015125416.2	4523	............G.F..E.............................R...........V...............	4873
XM_005591100.2	4677	............G.F..E.............................R...........V...............	5027
XM_015442592.1	4735	............G.F..E.............................R...........V...............	5085
XM_030798058.1	4139	............GL...S.....................................R.......	4489
XM_030798060.1	4266	............GL...S.....................................R.......	4616
XM_030798059.1	4341	............GL...S.....................................R.......	4691
XM_025371130.1	4458	............G.F..E............R................R...........................	4808
XM_004057100.3	4510	............G...Q.......Y......................	4860
XM_011961313.1	4006	............G.F...P........................R...........................	4356
XM_012030324.1	3900	............G.F..E.............................R...Q.......................	4250
XM_012030325.1	3929	............G.F..E.............................R...Q.......................	4279
XM_012030322.1	3985	............G.F..E.............................R...Q.......................	4335
XM_012030326.1	4241	............G.F..E.............................R...Q.......................	4591
XM_033235914.1	4168	............G.F..R.........P.................	4518
XM_033235915.1	4464	............G.F..R.........P.................	4814
XM_017953150.3	4005	............G.F..E......................K......R...........................	4355
XM_009195879.4	4118	............G.F..E......................K......R...........................	4468
XM_003916462.4	4210	............G.F..E......................K......R...........................	4560
XM_017886371.1	4073	............G.F..R.....V.....................	4423
XM_017886370.1	4124	............G.F..R.....V.....................	4474
XM_010376034.2	4462	............G.F..R.T...V.....................	4812
XM_010376033.2	4519	............G.F..R.T...V.....................	4869
XM_032257970.1	4077	............G......................................A..........S.....................G....R........D..M...............	4427
XM_017504465.2	4107	............G......................................A..........S.....................G....R........D..M...............	4457
XM_017504466.2	4241	............G......................................A..........S.....................G....R........D..M...............	4591
XM_032257971.1	4253	............G......................................A..........S.....................G....R........D..M...............	4603
XM_003928473.3	4519	............G....................................P.A..........S.....................G.............D..M...............	4869
XM_010339128.2	4576	............G....................................P.A..........S.....................G.............D..M...............	4926

```
XM_035266797.1  3851  ............G......................................A.......N.GS................I....G.Q...........D..M...............  4201
XM_035266794.1  3908  ............G......................................A.......N.GS................I....G.Q...........D..M...............  4258
XM_035266798.1  3953  ............G......................................A.......N.GS................I....G.Q...........D..M...............  4303
XM_035266795.1  3962  ............G......................................A.......N.GS................I....G.Q...........D..M...............  4312
XM_035266796.1  4054  ............G......................................A.......N.GS................I....G.Q...........D..M...............  4404
XM_035266799.1  4379  ............G......................................A.......N.GS................I....G.Q...........D..M...............  4729
XM_002755871.5  4543  ............G......................................A.......N.GS................I....G.Q...........D..M...............  4893
XM_017978685.2  4600  ............G......................................A.......N.GS................I....G.Q...........D..M...............  4950
XM_021667211.1  3830  ............G.V....................................AE........GS.....................G.Q...........D..M...............  4180
XM_012441938.2  3885  ............G.V....................................AE........GS.....................G.Q...........D..M...............  4235
XM_012441936.2  3994  ............G.V....................................AE........GS.....................G.Q...........D..M...............  4344
XM_012441937.2  4491  ............G.V....................................AE........GS.....................G.Q...........D..M...............  4841
XM_008071124.2  3821  ...........NG.V....................................S..M.....N.M................I....K.........N..E.M.M...............  4171
XM_008846235.2  4182  .........M.NG.V....................................S........DN.....V...S....R........C...........ED..................  4532
XM_008846225.2  4239  .........M.NG.V....................................S........DN.....V...S....R........C...........ED..................  4589
XM_028760768.1  2095  ...........NG.V....................................S..A.....D...T.E....S....R........F...........ED..V...............  2445
XM_016977522.3  3774  .........M.NG.V....................................T........DN....EV...C....R........F...........ED..................  4124
XM_027439265.2  3831  .........M.NG.V....................................T........DN....EV...C....R........F...........ED..................  4181
XM_027424023.2  4370  .........M.NG.V....................................T........DN....EV...C....R........F...........ED..................  4720
XM_027424022.2  4429  .........M.NG.V....................................T........DN....EV...C....R........F...........ED..................  4779
XM_010644067.2  3820  .........V.NG.V....................................S........DN.........S.......I.V...C...........E.M.M..........I....  4170
XM_010644066.2  3835  .........V.NG.V....................................S........DN.........S.......I.V...C...........E.M.M..........I....  4185
XM_010644065.2  3877  .........V.NG.V....................................S........DN.........S.......I.V...C...........E.M.M..........I....  4227
XM_006984700.3  4050  .........M.NG.V....................................S........DNA....V...C.......A.....F...........ED..................  4400
XM_006984699.3  4107  .........M.NG.V....................................S........DNA....V...C.......A.....F...........ED..................  4457
XM_036195690.1  4479  .........M.NG.V....................................S........DN.....V..PC.............F...........ED..V...............  4829
XM_012647817.1  3811  .........M.NG.V....................................P........N......T...S.......I....KF...Q....N..E.M.M...............  4161
XM_012647818.1  4255  .........M.NG.V....................................P........N......T...S.......I....KF...Q....N..E.M.M...............  4605
XM_028894802.2  3789  .........M.NG.V....................................S........DNA.T..V...C.......A.....F...........ED..................  4139
XM_028894803.1  4487  .........M.NG.V....................................S........DNA.T..V...C.......A.....F...........ED..................  4837
XM_020281624.1  4027  ...........NG.V....................................P........N......TR..S.......I....KF...Q....N..E.M.M...............  4377
XM_020281621.1  4084  ...........NG.V....................................P........N......TR..S.......I....KF...Q....N..E.M.M...............  4434
XM_012743627.2  4139  ...........NG.V....................................P........N......TR..S.......I....KF...Q....N..E.M.M...............  4489
XM_012743628.1  4247  ...........NG.V....................................P........N......TR..S.......I....KF...Q....N..E.M.M...............  4597
XM_020281622.1  4646  ...........NG.V....................................P........N......TR..S.......I....KF...Q....N..E.M.M...............  4996
XM_008523381.1  3749  .........M.NG.V.H.................................NL........NN.........S.......I.....C........S..E.M.M...............  4099
XM_014833099.1  3754  .........M.NG.V.H.................................NL........NN.........S.......I.....C........S..E.M.M...............  4104
XM_001502248.4  3881  .........M.NG.V.H.................................NL........NN.........S.......I.....C........S..E.M.M...............  4231
XM_011376495.2  3775  ...........NG.V...................................NS.......ENN.....C...S.............C........S..E.M.M...............  4125
XM_006913876.3  3824  ...........NG.V...................................NS.......ENN.....C...S.............C........S..E.M.M...............  4174
XM_039885188.1  3882  ...........NG.V...................................NS.......ENN.....C...S.............C........S..E.M.M...............  4232
XM_024553302.1  3747  ...........NG.V...................................NL.......ENN.....T...S.............C........S..EDM.M...............  4097
XM_037029937.1  4324  ...........NG.V.................................I.NL.......ENN.....T...S.............C........S..EDM.M...............  4674
XM_026385641.1  4347  ...........NG.V....................................S........DNA....L...S.......I.....C........E..EDV.A.......R.......  4697
XM_021734874.2  3893  ...........NG.V....................................S........DNA....L...S.......I.....C........E..EDV.A.......R.......  4243
XM_005337738.4  3976  ...........NG.V....................................S........DNA....L...S.......I.....C........E..EDV.A.......R.......  4326
XM_028145659.1  4267  ...........NG.V...................................NL..P....EN......S...S.............C.......RS..ED..M...............  4617
XM_008141648.2  4308  ...........NG.V...................................NL..P....EN......S...S.............C.......RS..ED..M...............  4658
XM_028145658.1  4314  ...........NG.V...................................NL..P....EN......S...S.............C.......RS..ED..M...............  4664
XM_023755256.1  3746  ...........NG.V...................................NL..P....ENN.....P...S.............C........S..E...M...............  4096
XM_014457296.2  3787  ...........NG.V...................................NL..P....ENN.....P...S.............C........S..E...M...............  4137
XM_005081664.4  4356  .........M.NG.V....................................T........DNA.T.EV...C....R........F...........EDA.................  4706
XM_013121709.3  4415  .........M.NG.V....................................T........DNA.T.EV...C....R........F...........EDA.................  4765
XM_028523512.2  4320  ...........NG.V...................................NL.......ENN.....T...S.........G...C........S..EDM.M...............  4670
```

XM_037154685.1	4063	...........NG.V...................................NL.......ENN.....T...S.........T...C........S..EDM.M...............	4413
XR_004952390.1	4323	.........M.NG.V...................................NS.......ENN.....C...S.............C........S..E.M.M...............	4673
XM_016166586.2	4323	.........M.NG.V...................................NS.......ENN.....C...S.............C........S..E.M.M...............	4673
XM_027940528.1	3951	...........NG.V....................................S........DNA....L...S.......I.....CH.......E..EDV.A...............	4301
XM_015490800.1	3753	...........NG.V....................................S........DNA....L...S.......I.....CH.......E..EDV.A...............	4103
XM_004438122.2	3889	.........M.NG.V.................................I.NS........NN.....T...S.............C.......ES..E.M.M...............	4239
XM_004864770.3	4016	.........M.NG.V....................................L........DN....NT...S.......IRV...C...........EDM.M..T..F.........	4366
XM_004864769.3	4033	.........M.NG.V....................................L........DN....NT...S.......IRV...C...........EDM.M..T..F.........	4383
XM_004864768.3	4075	.........M.NG.V....................................L........DN....NT...S.......IRV...C...........EDM.M..T..F.........	4425
XM_021632415.1	3839	.........M.SGAV..................................P.S........D.....EV...S....R........F...R.M......D..V......S........	4189
XM_021632414.1	4105	.........M.SGAV..................................P.S........D.....EV...S....R........F...R.M......D..V......S........	4455
XM_021632416.1	4471	.........M.SGAV..................................P.S........D.....EV...S....R........F...R.M......D..V......S........	4821
XM_036310740.1	3962	...........NG.V...................................NL..P....ENN.....P...S.............C........T..ED..M...............	4312
XM_036310741.1	4258	...........NG.V...................................NL..P....ENN.....P...S.............C........T..ED..M...............	4608
XM_036310738.1	4300	...........NG.V...................................NL..P....ENN.....P...S.............C........T..ED..M...............	4650
XM_036310739.1	4306	...........NG.V...................................NL..P....ENN.....P...S.............C........T..ED..M...............	4656
XM_005873010.2	3709	...........NG.V...................................NL..P....ENN.....P...S.............C........S..ED..V...........C...	4059
XM_016211348.1	3619	...........NGIV...................................NL...C...ENN.....P...S.......I.....C........T..EDM.M...............	3969
XM_016211347.1	3724	...........NGIV...................................NL...C...ENN.....P...S.......I.....C........T..EDM.M...............	4074
XM_029544431.1	4464	.........M.NG.V....................................S..A.....D.A.T.E....S....R........FP........N.ED..V...............	4814
XM_021209372.2	4480	.........M.NG.V....................................S..A.....D.A.T.E....S....R........FP........N.ED..V...............	4830
XM_013517880.1	4127	.........V.NG.V..................................P.S........DN.....M...S........RV...CQ..........EDM.M...............	4477
XM_013517879.1	4184	.........V.NG.V..................................P.S........DN.....M...S........RV...CQ..........EDM.M...............	4534
XR_002125999.1	3832	...........NG.V...................................NS.......ENN.....T.V.S..L..........C........S..E.M.M...............	4182
XR_002126000.1	3832	...........NG.V...................................NS.......ENN.....T.V.S..L..........C........S..E.M.M...............	4182
XM_019660329.1	3771	...........NG.V...................................NS.......ENN.....T.V.S..L..........C........S..E.M.M...............	4121
XM_019660327.1	3824	...........NG.V...................................NS.......ENN.....T.V.S..L..........C........S..E.M.M...............	4174
XM_019660326.1	3832	...........NG.V...................................NS.......ENN.....T.V.S..L..........C........S..E.M.M...............	4182
XM_031361021.1	4416	...........NG.V....................................S..A.....D...T.EV.P.S....R........F........EG.ED..V...............	4766
XM_031361023.1	4489	...........NG.V....................................S..A.....D...T.EV.P.S....R........F........EG.ED..V...............	4839
XM_006757881.2	3712	...........NG.V...................................NL..P...PENN.....P...S.............C........S..ED..M...............	4062
LR877236.1	10051145	.........M.NG.V....................................S.......EDN....EV...S....R........F..........PED..A...............	10051495
LR738629.1	3248049	...........NG.V..................................H.S.......EDN.....V...S.......I.....C....P...E..EDV.A.......R.......	3247699
LR738608.1	38419984	...........NG.V..................................H.S.......EDN.....V...S.......I.....C....P...E..EDL.A.......R.......	38420334
XM_036279846.1	3749	...........NG.V...................................NM.......ENN.....P.M.S.............C......D.S..EDM.M.............R.	4099
XM_029470752.1	4464	.........M.NG.V....................................S..A.....D.....E..P.S.............FP........N.EDI.V...............	4814
XM_021184932.1	4486	.........M.NG.V....................................S..A.....D.....E..P.S.............FP........N.EDI.V...............	4836
XM_041675907.1	4433	.........M.NGLV....................................S........DN....EV...F....R..A.....F......G.EE.KD..................	4783
XM_041675906.1	4491	.........M.NGLV....................................S........DN....EV...F....R..A.....F......G.EE.KD..................	4841
XM_039085849.1	3074	...........NG.V...........................T........L..A.....DGA.T.E.T..S....R........F........E..ED..A...............	3424
NM_001371690.1	3936	...........NG.V...........................T........L..A.....DGA.T.E.T..S....R........F........E..ED..A...............	4286
XM_008767525.3	4473	...........NG.V...........................T........L..A.....DGA.T.E.T..S....R........F........E..ED..A...............	4823
AK136458.1	779	...........NG.V....................L...............S..A.....D.....E.R..S..S.R........FP........S.EDI.V...S..S........	1129
XM_033101188.1	3792	...........NG.V...................................NA.......ENNA....T.V.S..L..........C........S..E.M.M..............M	4142
XM_033101185.1	4306	...........NG.V...................................NA.......ENNA....T.V.S..L..........C........S..E.M.M..............M	4656
XM_033101187.1	4322	...........NG.V...................................NA.......ENNA....T.V.S..L..........C........S..E.M.M..............M	4672
Z50190.1	3751	...........NG.V....................................S..A.....D.....E....S..S.R........FP........S.EDI.V...S..S........	4101
XM_038325775.1	4102	.........M.NGLV....................................S........DN....EV...C....R..A.....F......G..EGED..................	4452
XM_038325774.1	4160	.........M.NGLV....................................S........DN....EV...C....R..A.....F......G..EGED..................	4510
XM_005349243.3	4061	.........M.NGLV....................................S........DN....EV...S....R..A.....F......G.EESED..................	4411
XM_026780347.1	4118	.........M.NGLV....................................S........DN....EV...S....R..A.....F......G.EESED..................	4468
XR_005031789.1	3920	.........M.SG.V...................................NL........NNS....T...SR......S.....C........S..EDM.M...S...........	4270
XM_036920445.1	3920	.........M.SG.V...................................NL........NNS....T...SR......S.....C........S..EDM.M...S...........	4270
XM_028521413.2	2023	.......N...NG.V...................................NL.......ENN.....TR..S.........G...C........S..EDM.M...G...........	2373

XM_004373208.2	3864	.........M.NGIV.R.................................NL..A............V..LS....R...R.......SR....S..E.V.L.D........A.R..	4214
XM_005007464.2	3897	.........V.NG.V..................................P.S........DN.P...T..YS.......I.V...CQ..........EDM.M...............	4247
XM_013141971.1	3954	.........V.NG.V..................................P.S........DN.P...T..YS.......I.V...CQ..........EDM.M...............	4304
XM_013011221.1	1999	.........M.NGIV....................................S..M.....DN.....M...S..L....I.....C.S......E..ED.EM...............	2349
XM_032914298.1	3911	...........NG.V...........................T........L..A.....DGA.T.E.T..S....R........F........E.SED..A...............	4261
XM_032914300.1	3936	...........NG.V...........................T........L..A.....DGA.T.E.T..S....R........F........E.SED..A...............	4286
XM_038669351.1	3843	.........M.SGAV...................................N........ENNVL...T...S.............C........S..E.A.M...S...R..I....	4193
XM_005621614.3	3843	.........M.SGAV...................................N........ENNVL...T...S.............C........S..E.A.M...S...R..I....	4193
XM_025416703.2	3843	.........M.SGAV...................................N........ENNVL...T...S.............C........S..E.A.M...S...R..I....	4193
XM_038669350.1	3924	.........M.SGAV...................................N........ENNVL...T...S.............C........S..E.A.M...S...R..I....	4274
XM_022420138.2	3924	.........M.SGAV...................................N........ENNVL...T...S.............C........S..E.A.M...S...R..I....	4274
XM_025416701.2	3924	.........M.SGAV...................................N........ENNVL...T...S.............C........S..E.A.M...S...R..I....	4274
HG994389.1	37923502	.........M.SGAV...................................N........ENNVL...T...S.............C........S..E.A.M...S...R..I....	37923852
CP050622.1	38288897	.........M.SGAV...................................N........ENNVL...T...S.............C........S..E.A.M...S...R..I....	38289247
CP050586.1	39068297	.........M.SGAV...................................N........ENNVL...T...S.............C........S..E.A.M...S...R..I....	39068647
XM_036410224.1	4323	.......R...SG.V...................................NL..P...PEN.A....P...S........R....C........G..EDA.M...............	4673
XM_036410225.1	4329	.......R...SG.V...................................NL..P...PEN.A....P...S........R....C........G..EDA.M...............	4679
XM_042698254.1	3908	.........M.NGIV....................................S..M.....DN.....M...S..L....I.....C.S......E..ED.EM...............	4258
XM_042698253.1	3965	.........M.NGIV....................................S..M.....DN.....M...S..L....I.....C.S......E..ED.EM...............	4315
XR_005060264.1	3924	.........M.SG.V...........................T.......NL........NNS....T...SR......S.....C........S..EDM.M...S...........	4274
XR_005060266.1	3924	.........M.SG.V...........................T.......NL........NNS....T...SR......S.....C........S..EDM.M...S...........	4274
XM_037014462.1	3924	.........M.SG.V...........................T.......NL........NNS....T...SR......S.....C........S..EDM.M...S...........	4274
XM_037014469.1	4006	.........M.SG.V...........................T.......NL........NNS....T...SR......S.....C........S..EDM.M...S...........	4356
XM_032328639.1	4005	.........M.SGAV...................................N......C.ENNM........S.........P...S........S..E.V.V...S...R.......	4355
XM_032328640.1	4212	.........M.SGAV...................................N......C.ENNM........S.........P...S........S..E.V.V...S...R.......	4562
XM_032328638.1	4358	.........M.SGAV...................................N......C.ENNM........S.........P...S........S..E.V.V...S...R.......	4708
LR738420.1	3196749	.........M.SGAV...................................NA.......ENNM........SN........P...S........S..E.V.V...S...R..I....	3196399
XM_004750535.2	3934	.........M.SGAV...................................NA.......ENNM....T...S.........P...S........S..E.V.M...S...R.......	4284
XM_013055032.1	4182	.........M.SGAV...................................NA.......ENNM....T...S.........P...S........S..E.V.M...S...R.......	4532
XM_013055031.1	4214	.........M.SGAV...................................NA.......ENNM....T...S.........P...S........S..E.V.M...S...R.......	4564
U30602.1	3782	...........NG.V....................................S..A.....D.....E.R..S..S.R........FP........S.EDI.V...S..S........	4132
XM_011245806.2	3882	...........NG.V....................................S..A.....D.....E.R..S..S.R........FP........S.EDI.V...S..S........	4232
NM_001291910.1	4252	...........NG.V....................................S..A.....D.....E.R..S..S.R........FP........S.EDI.V...S..S........	4602
NM_009624.3	4475	...........NG.V....................................S..A.....D.....E.R..S..S.R........FP........S.EDI.V...S..S........	4825
XM_011245805.3	4806	...........NG.V....................................S..A.....D.....E.R..S..S.R........FP........S.EDI.V...S..S........	5156
AC132575.3	137289	...........NG.V....................................S..A.....D.....E.R..S..S.R........FP........S.EDI.V...S..S........	136939
AC087799.43	160441	...........NG.V....................................S..A.....D.....E.R..S..S.R........FP........S.EDI.V...S..S........	160791
AK156940.1	4254	...........NG.V....................................S..A.....D.....E.R..S..S.R........FP........S.EDI.V...S..S........	4604
XM_017338218.1	4285	...........NG.V............................T.......S......CENA-..R...P.S.......I.....C...........EDV.L.........D.....	4632
XM_022516779.1	3730	.........M.SGAV...................................NA.......ENNM........SN......I.P...S........SN.E.V.V...S...R.......	4080
XM_026520438.1	3993	.........M.SGAV...................................NA..M....ENNV....I...S.......F.....C........S..E.A.M...S...R.......	4343
XM_026520440.1	4178	.........M.SGAV...................................NA..M....ENNV....I...S.......F.....C........S..E.A.M...S...R.......	4528
XM_008696832.2	3731	.........M.SGAV...................................NA..M....ENNV....I...S.......F.....C........S..E.A.M...S...R.......	4081
XM_034505845.1	4479	...........NG.V....................................S..A.....D.A.T.ET...S....R..G.....F..........SEDP.A..T............	4829
XM_025859061.1	3876	.........M.SGAV...................................NA..A....EN.V....T...L.......L.....CQ.......S..E.V.V...S...R.......	4226
XM_025859060.1	3918	.........M.SGAV...................................NA..A....EN.V....T...L.......L.....CQ.......S..E.V.V...S...R.......	4268
XM_036090826.1	4145	.........M.SGAV...................................NT.RA....EN.V....T...L.........T...C........S..E.V.M...S...R.......	4495
XM_036090830.1	3706	.........M.SGAV...................................NT.RA....EN.V....T...L.........T...C........S..E.V.M...S...R.......	4056
XM_032397753.1	3963	.........M.SGAV...................................NT.RA....EN.V....T...L.........T...C........S..E.V.M...S...R.......	4313
XM_036090838.1	4105	.........M.SGAV...................................NT.RA....EN.V....T...L.........T...C........S..E.V.M...S...R.......	4455
XM_032397752.1	3984	.........M.SGAV...................................NT.RA....EN.V....T...L.........T...C........S..E.V.M...S...R.......	4334
XM_026003009.1	3880	.........M.SGAV...................................NA.......ENNVP...T...S.......L.....C........S..E.A.M...S...R..I....	4230
XM_041747061.1	3883	.........M.SGAV...................................NA.......ENNVP...T...S.......L.....C........S..E.A.M...S...R..I....	4233
XM_041747062.1	3890	.........M.SGAV...................................NA.......ENNVP...T...S.......L.....C........S..E.A.M...S...R..I....	4240

```
XM_003417743.3  3806      .........I.NGIV...................................NL.RA..S.........V...S....R...R...SC..S..M..S..E.V.L...........G.L  4156
XM_027611017.2  4293      .........M.SGAV...................................NA..A....EN.V....T...L.......L.....C.........S..E.VDV...S...R.......  4643
XM_028117546.1  3877      .........M.SGAV...................................NA..A....EN.V....T...L.......L.....C.........S..E.VDV...S...R.......  4227
XM_028117545.1  3919      .........M.SGAV...................................NA..A....EN.V....T...L.......L.....C.........S..E.VDV...S...R.......  4269
XM_004403221.2  3752      .........M.SGAV...................................NA..A....EN.V....T...L..L....L.....C.........S..E.V.M...S...R.......  4102
XM_034670470.1  3898      .........M.SGAV...................................NA..M..S.ENNV....T...S.......F.....C.........S..E.A.V...S...R.......  4248
XM_035022136.1  4117      .........M.SGA....................................NM.RA....EN.V....T...L..L......T...C.........S..E.V.M...S...R.......  4467
XM_035022135.1  4159      .........M.SGA....................................NM.RA....EN.V....T...L..L......T...C.........S..E.V.M...S...R.......  4509
XM_035022134.1  4193      .........M.SGA....................................NM.RA....EN.V....T...L..L......T...C.........S..E.V.M...S...R.......  4543
XM_032849349.1  4247      .........M.SGAV........................E...S......NA.......ENNM.........SN.........P...S.....V..S..E.V.V...S...R.......  4597
XM_006744470.2  2020      .........M.SGAV...................................NM.RA....EN.V....T...L..L......T...C.........S..E.V.M...S...R.......  2370
XM_021698379.1  3863      .........M.SGAV...................................NM.RA....EN.V....T...L..L......T...C.........S..E.V.M...S...R.......  4213
XM_023711480.1  4422      .........VNNG.V..................................P.S......F.D.M....TYM.S.......SI.V...C..........SEDM.M...............  4772
XM_023711479.1  4479      .........VNNG.V..................................P.S......F.D.M....TYM.S.......SI.V...C..........SEDM.M...............  4829
XM_027859744.1  4203      .........M.SGIV.H................................HNS....Y....N.....V...S.K.R.D.......C.........S..G.G.V...........I....  4553
XM_027859742.1  4206      .........M.SGIV.H................................HNS....Y....N.....V...S.K.R.D.......C.........S..G.G.V...........I....  4556
XM_020156525.1  1189      .........M.NG.V....................................L.......EDN.....V...S........I.T...C............E.AEM................  1539
XM_003761887.4  4219      .........M.SGIV.H................................HSS....Y....N.........A.K.R.D.......C.........S..G.ME...........I....  4569
XM_023497722.2  4222      .........M.SGIV.H................................HSS....Y....N.........A.K.R.D.......C.........S..G.ME...........I....  4572
XM_020915285.1  3881      ...........SGLV.................................I.NP.QV....ENNA.TR...P.A........IR....C.........S..E.A.M.............    4225
XM_020915286.1  3944      ...........SGLV.................................I.NP.QV....ENNA.TR...P.A........IR....C.........S..E.A.M.............    4288
OU343087.1      18081159  ...........SGLV.................................I.NP.QV....ENNA.TR...P.A........IR....C.........S..E.A.M.............    18080815
XM_036825406.1  3885      ...........NGMV............................T.......NP.QV....ENN...R.T.P.S........I.....C..S.....S..E.A.V.............    4229
XM_023513810.1  3840      ...........NG.V....................................P........N......TY..S.......SL....KCQ.VQTL.EN..EDM.M...............  4193
                                                                                     \
                                                                                     |
                                                                                     V
XM_003790833.3  4145      ...........NG.V....................................P........N......TY..S.......SL....KCQ.VQTL.EN..EDM.M...............  4498
                                                                                     \
                                                                                     |
                                                                                     V
XM_023513809.1  4202      ...........NG.V....................................P........N......TY..S.......SL....KCQ.VQTL.EN..EDM.M...............  4555
                                                                                     \
                                                                                     |
                                                                                     V
XM_032461074.1  4479      ...........SGMV...................................NP.QA.....HNL....T..TS.......I.....C.........S.Y..M.L..TS.........RR.  4829
XM_020995690.1  4472      .........M.SGIV.H................................TIX....Y...TN.....V...S.K.R.D.......Y.........S..G.V.A...........I....  4822
XM_010949805.1  3962      ...........SGMV...................................NP.QA.....HNL....T..TS.......I.....C.........S.Y..M.L..TS.........RR.  4312
OU015368.1      58376306  .........M.NG.V....................................S.N......DN....EV...S....R.........FG..........PED..A.........      58376638
XM_031447846.1  3879      ...........SGMV...................................NP.QA.....HNL....T..TS.......I.....C.........S.Y..M.L..TS.........QR.  4229
XM_031447845.1  3882      ...........SGMV...................................NP.QA.....HNL....T..TS.......I.....C.........S.Y..M.L..TS.........QR.  4232
XM_006204318.3  3943      ...........SGMV...................................NP.QA.....HNL....V..TS.......I.....C.........S.Y..M.L..TS.........QR.  4293
XM_028487286.1  918       ...........NGMV............................T.......NP.QV....ENN...R.T.P.S.K.....I.....C..S.....S..E.A.V.............    1262
XM_027117551.1  4298      ...........NGMV............................T.......NP.QV....ENN...R.T.P.S.K.....I.....C..S.....S..E.A.V.............    4642
XM_027117552.1  4338      ...........NGMV............................T.......NP.QV....ENN...R.T.P.S.K.....I.....C..S.....S..E.A.V.............    4682
XM_024759606.1  3806      ...........NGMV............................T.......NP.QV....ENN...R.T.P.S.K.....I.....C..S.....S..E.A.V.............    4150
XM_033839105.1  3860      ...........NGMV............................T.......NP.QV....ENN...R.T.P.S.K.....I.....C..S.....S..E.A.V.............    4204
XM_033839104.1  3861      ...........NGMV............................T.......NP.QV....ENN...R.T.P.S.K.....I.....C..S.....S..E.A.V.............    4205
XM_030848578.1  3860      ...........NGMV............................T.......NP.QV....ENN...R.T.P.S.K.....I.....C..S.....S..E.A.V.............    4204
XM_030848577.1  3861      ...........NGMV............................T.......NP.QV....ENN...R.T.P.S.K.....I.....C..S.....S..E.A.V.............    4205
XM_033428926.1  3860      ...........NGMV............................T.......NP.QV....ENN...R.T.P.S.K.....I.....C..S.....S..E.A.V.............    4204
XM_004270236.3  3861      ...........NGMV............................T.......NP.QV....ENN...R.T.P.S.K.....I.....C..S.....S..E.A.V.............    4205
XM_027117553.1  4428      ...........NGMV............................T.......NP.QV....ENN...R.T.P.S.K.....I.....C..S.....S..E.A.V.............    4772
XM_024759607.1  3890      ...........NGMV............................T.......NP.QV....ENN...R.T.P.S.K.....I.....C..S.....S..E.A.V.............    4234
```

XM_030848580.1	3942	..........NGMV.........................T.......NP.QV....ENN...R.T.P.S.K.....I.....C..S.....S..E.A.V............	4286
XM_032606056.1	4099	..........NGMV.........................T.......NP.QV....ENN...R.T.P.S.K.....I.....C..S.....S..E.A.V............	4443
XM_033839103.1	4469	..........NGMV.........................T.......NP.QV....ENN...R.T.P.S.K.....I.....C..S.....S..E.A.V............	4813
XM_033428925.1	4473	..........NGMV.........................T.......NP.QV....ENN...R.T.P.S.K.....I.....C..S.....S..E.A.V............	4817
XM_030763626.1	4133	..........NGMV.........................T.......NP.QV....ENN...R.T.P.S.K.....I.....C..S.....S..E.A.V............	4477
XM_022594740.2	4326	..........NGMV.........................T.......NP.QV....ENN...R.T.P.S.K.....I.....C..S.....S..E.A.V............	4670
XM_022594741.2	4416	..........NGMV.........................T.......NP.QV....ENN...R.T.P.S.K.....I.....C..S.....S..E.A.V............	4760
XM_022594743.2	4530	..........NGMV.........................T.......NP.QV....ENN...R.T.P.S.K.....I.....C..S.....S..E.A.V............	4874
XM_030763625.1	4618	..........NGMV.........................T.......NP.QV....ENN...R.T.P.S.K.....I.....C..S.....S..E.A.V............	4962
XM_008573823.1	3713	..........NG.V...................................S.....V..NN.......P.S..A....G.....C.I......N..E.L.M...D.....S.	4051
XM_018040729.1	3905	..........SGLV..........................S......NP.QV....ENNA.TR...S.A........R....C........S..E.A.M............E	4249
XM_029220189.1	4119	..........NGMV.........................T.....I.NP.QV....ENN...R.T.P.S.K.....I.....C..S.....S..E.A.V............	4463
XR_450938.1	3843	..........SGMV.........................T.....L.NP.QV....ENN...R.T.P.S.......I.....C..S.....S..E.A.V............	4187
XR_450939.1	3843	..........SGMV.........................T.....L.NP.QV....ENN...R.T.P.S.......I.....C..S.....S..E.A.V............	4187
XM_007181542.1	3843	..........SGMV.........................T.....L.NP.QV....ENN...R.T.P.S.......I.....C..S.....S..E.A.V............	4187
XM_019468420.1	3773	..........SGAV.................................SA...PP..ENNV..R.....S.............C........S..EDADA...S...R.....E..	4123
XM_019468419.1	3865	..........SGAV.................................SA...PP..ENNV..R.....S.............C........S..EDADA...S...R.....E..	4215
XM_007073390.2	3682	..........SGAV.................................SA...PP..ENNV..R.....S.............C........S..EDADA...S...R.....E..	4032
XM_006874904.1	3718	........M.NGIV.........................S.......NL.RA.V...G.P.PR.I....S.........R.....S.L.Q...ES.Y..V.L............G.	4068
XM_039239300.1	4088	..........SGAV............................P.....SA...PP..ENNV........S.............CQ.......S..EDA.A...S...R..I.EG.	4438
XM_027961718.2	3907	..........SGLV..........................S......NP.QV.P..ENNA.TR...S.A........R....C........S..E.A.M............E	4251
XM_040255712.1	3796	..........SGLV..........................S......NL.QV.P..ENNA.TR.S.S.A........R....CQ.......S..E.A.M............E	4140
XM_006054867.2	3882	..........SGLV..........................S......NP.QV.P..ENNA.TR...P.A........R....C........S..E.A.M............E	4226
XM_005901318.1	3712	..........SGLV..........................S......NP.QV.P..ENNA.TR...P.A........R....C........S..E.V.M............E	4056
XM_019987469.1	3882	..........SGLV..........................S......NP.QV.P..ENNA.TR...P.A........R....C........S..E.V.M............E	4226
XM_019987468.1	3885	..........SGLV..........................S......NP.QV.P..ENNA.TR...P.A........R....C........S..E.V.M............E	4229
XM_010840395.1	3918	..........SGLV..........................S......NP.QV.P..ENNA.TR...P.A........R....C........S..E.V.M............E	4262
CP027093.1	3248706	..........SGLV..........................S......NP.QV.P..ENNA.TR...P.A........R....C........S..E.V.M............E	3248362
XM_029947248.1	3746	........A.SGAV..........................S......ST...PP.AENNV..R.....S.......AI....C........S..E.A.AK..S...R.....E..	4096
NM_001205917.1	3712	..........SGLV..........................S......NP.QVPP..ENNA.TR...P.A........R....C........S..E.V.M............E	4056
XM_027528206.1	3867	..........SGLV..........................S......NP.QVPP..ENNA.TR...P.A........R....C........S..E.V.M............E	4211
XM_027528205.1	3870	..........SGLV..........................S......NP.QVPP..ENNA.TR...P.A........R....C........S..E.V.M............E	4214
XM_005224482.4	3916	..........SGLV..........................S......NP.QVPP..ENNA.TR...P.A........R....C........S..E.V.M............E	4260
XM_005224481.4	3919	..........SGLV..........................S......NP.QVPP..ENNA.TR...P.A........R....C........S..E.V.M............E	4263
XM_001363517.4	5323	........M.SGIV.H...............................HNS...TY....NSHS..VQ..S.KSR.D.I.T....C........S..E..............I....	5673
XM_007499075.2	5326	........M.SGIV.H...............................HNS...TY....NSHS..VQ..S.KSR.D.I.T....C........S..E..............I....	5676
XM_040973441.1	3963	..........NG.V...................................L.......P.-PV....T.P.S.......S.T...CQLSQ.M..E..EDVAM...D...........	4310
XM_040997511.1	3963	..........NG.V.................................CL.......P.-PV....T.P.S.......S.T...CQLSQ.M..E..EDVAM...............	4310
XM_004652218.1	3755	..........NG.V...............................P.P.........DNAL..E.........RAAA...D.C.....S..S.GADA.PD........T..T.	4102
XM_029058667.1	4218	........N.NGIV.H.................................NS.....H..ENN.E.R.I...S..LRRD.G.....C.....T..P..G.R.T..........I....	4568
XM_029058666.1	4221	........N.NGIV.H.................................NS.....H..ENN.E.R.I...S..LRRD.G.....C.....T..P..G.R.T..........I....	4571
XM_029058665.1	4311	........N.NGIV.H.................................NS.....H..ENN.E.R.I...S..LRRD.G.....C.....T..P..G.R.T..........I....	4661
XM_029058664.1	4314	........N.NGIV.H.................................NS.....H..ENN.E.R.I...S..LRRD.G.....C.....T..P..G.R.T..........I....	4664
XM_004586704.1	3721	..........NG.V...................................L......VP.-PV....T...S.......S.T...CHLSQ.M..E.YEDVAM...D..........	4068
XM_006140748.3	3761	........AGNG.V..........................S..LAG..S..V....A..........ASE.A.R..AST....C.L....A.RNG.E.P.VD...D...........	4111
XM_027773156.1	3818	........AGNG.V..........................S..LAG..S..V....A..........ASE.A.R..AST....C.L....A.RNG.E.P.VD...D...........	4168
XM_023246667.1	3856	..........SGAV.................................SA...PP..ENNV..R.G...S.............C........S..E.A.A...S...R.....E..	4206
XM_032591551.1	3837	..........SGAV.................................SA...PP..ENNV..R.G...S.............C........S..E.A.A...S...R.....E..	4187
AP023167.1	38164101	..........SGAV.................................SA...PP..ENNV..R.G...S.............C........S..E.A.A...S...R.....E..	38164451
XM_012517947.2	3745	.......R...NG.V..............................G.SL.RAPP...G.A..R.VPP.S..L.....A.....C.A...L..S..EDA.M....D...........	4095
XM_023583430.1	4225	.......R...NG.V..............................G.SL.RAPP...G.A..R.VPP.S..L.....A.....C.A...L..S..EDA.M....D...........	4575
XM_025916850.1	3706	..........SGAV.................................SA...PP..ENNV..R.G...S.........T....C........S..E.A.A...S...R.....E..	4056
XM_004604370.1	3709	.....F.....NGGV................................S.E..P..P.D.A....TQPTS..L.....AR....A.LSR.L.SS...DV.L.......R.....PR.	4059
XM_027043368.1	3860	..........SGAV.................................SA...PP..ENNM..R.D...S.........T....C........S..E.A.A...S...R.....E..	4210

XM_038763009.1	3880	.........N.NGIV.H.................................NS....H..ENN.E.R.L.IPS.KLRRD.G....KC.....T..P..G.R.T..........I....	4230
XM_038763008.1	3976	.........N.NGIV.H.................................NS....H..ENN.E.R.L.IPS.KLRRD.G....KC.....T..P..G.R.T..........I....	4326
NM_204630.2	3703	.........M.NGIV.H.................................NS...AH.T.N.-ET...LP.S.KLQ...T.....C.....V..T..E.A.T..V............	4050
XM_015294128.3	3734	.........M.NGIV.H.................................NS...AH.T.N.-ET...LP.S.KLQ...T.....C.....V..T..E.A.T..V............	4081
XM_007453480.1	3752	...........NGMV...........................T.......NP.QV....ENN...R.T.P.S.K.....I.....C..S.....S..E.A.E.............	4096
XM_037813799.1	4228	.........M.NG.V...........................T.....M.NL..........S....SLP....L....SR....CPLS..S..S..E...L...S...	4554
XM_019540007.1	3978	.........M.NGIV.H.................................NS..I.RS..N.SEP..MLP.S..LQ.DS....D.C........NN.ED..T..........I....	4328
XM_030958382.1	4021	.........M.NGIV.H.................................NS..IAH.T...-E...ILP.A.KLQ.DAA.....C.....V.....E...T..V.......I....	4368
XM_030958381.1	4125	.........M.NGIV.H.................................NS..IAH.T...-E...ILP.A.KLQ.DAA.....C.....V.....E...T..V.......I....	4472
XM_005425270.2	3481	.........M.NGIV.H.................................NS..IAH.T...-E...ILP.A.KLQ.DAA.....C.....V.....E...T..V.......I....	3828
XM_036392098.1	4032	.........M.NGIV.H.................................NS..IAH.T...-E...ILP.A.KLQ.DAA.....C.....V.....E...T..V.......I....	4379
XM_036392099.1	4139	.........M.NGIV.H.................................NS..IAH.T...-E...ILP.A.KLQ.DAA.....C.....V.....E...T..V.......I....	4486
XM_019507552.1	3915	.........M.NGIV.H.................................NS..V.HS..N.SEP..MLP.S..LQ.DS....D.C.....V..NN.ED..T..........I....	4265
XM_040459179.1	3749	...........SGAV...................................SA...PP..ENNV..R.G...S...R....T....C........S..E.A.A...S...R....E..	4099
XM_040459181.1	3764	...........SGAV...................................SA...PP..ENNV..R.G...S...R....T....C........S..E.A.A...S...R....E..	4114
XM_006034256.3	1528	.........M.NGIV.H.................................NS..I.HS..N.SEP..MLP.S..LQ.DS....D.C.....V..NN.E.A.T..........I....	1878
XM_006266871.3	4064	.........M.NGIV.H.................................NS..I.HS..N.SEP..MLP.S..LQ.DS....D.C.....V..NN.E.A.T..........I....	4414
XM_014597457.2	4067	.........M.NGIV.H.................................NS..I.HS..N.SEP..MLP.S..LQ.DS....D.C.....V..NN.E.A.T..........I....	4417
AJ401469.1	3676	.........M.NGIV.H.................................NS...AHAT.N.-ET...LP.S.KLQ...T.....C.....V..T..E.A.T..V............	4023
XM_037507700.1	3749	.......R...SGLV............................S......SS.RA......GAP...PQPPA....RD.GR....C...R.VG.A.HED..L.D.....R.D...G.	4099
XM_009585784.1	839	.........M.NGIV.H.................................NS..IAH.T.N.-ET..ILP.T.KLQ.DA.R....C.....A..T..E.A.T..V.......I....	1186
XM_021086712.1	3887	.......R.S..GLV...................................TS.QA.PA..-N.P.R..PP.A....R..G.....C........S..E.S.A.DTD.....S.CR	4228
XM_021086709.1	3969	.......R.S..GLV...................................TS.QA.PA..-N.P.R..PP.A....R..G.....C........S..E.S.A.DTD.....S.CR	4310
XM_021086711.1	4353	.......R.S..GLV...................................TS.QA.PA..-N.P.R..PP.A....R..G.....C........S..E.S.A.DTD.....S.CR	4694
XM_021086710.1	4408	.......R.S..GLV...................................TS.QA.PA..-N.P.R..PP.A....R..G.....C........S..E.S.A.DTD.....S.CR	4749
XR_004369268.2	10456	.........M.NGIV.H.................................NS..IAH.T.N.-E...ILP.A.KLQ.DAA.....C.....T.....E...T..V.......I....	10109
XM_030285269.3	4065	.........M.NGIV.H.................................NS..IAH.T.N.-E...ILP.A.KLQ.DAA.....C.....T.....E...T..V.......I....	4412
XM_002194859.6	4171	.........M.NGIV.H.................................NS..IAH.T.N.-E...ILP.A.KLQ.DAA.....C.....T.....E...T..V.......I....	4518
XM_014252636.1	3726	.........M.NGIV.H.................................NS..IAH.T.N.-E...ILP.A.KLQ.DAA.....C.....V.....E.A.T..V.......I....	4073
XM_005522820.2	3831	.........M.NGIV.H.................................NS..IAH.T.N.-E...ILP.A.KLQ.DAA.....C.....V.....E.A.T..V.......I....	4178
XM_007521986.2	3852	...........NG.V.H.................................-------TP.PH.....YPP.R.......S.....C.....T.RS.WEDV.P...S...........	4181
XM_015642712.3	3784	.........M.NGIV.H.................................NS..IAH.T.N.-E...ILP.A.KLQ.DAA.....C.....V.....E.A.T..V.......I....	4131
XM_015642711.3	3889	.........M.NGIV.H.................................NS..IAH.T.N.-E...ILP.A.KLQ.DAA.....C.....V.....E.A.T..V.......I....	4236
XM_005492114.3	3763	.........M.NGIV.H.................................NS..IAH.T...-E...ILPPA.KLQ.DTA.....C.....V.....E...T..V.......I....	4110
XM_005492113.3	3869	.........M.NGIV.H.................................NS..IAH.T...-E...ILPPA.KLQ.DTA.....C.....V.....E...T..V.......I....	4216
XM_023936486.1	3672	.........M.NGIV.H.................................NS..IAH.T.N.-E...ILP.A.KLQ.DAA.....C.....M.....E.A.T..V.......I....	4019
XM_023936485.1	3776	.........M.NGIV.H.................................NS..IAH.T.N.-E...ILP.A.KLQ.DAA.....C.....M.....E.A.T..V.......I....	4123
XM_041409523.1	4055	.........M.NGIV.H.................................NS..VAH.T.N.-E...TLP.A.KLQ.DAA.....CG....V.....E...T..V.......I....	4402
XM_041409514.1	4069	.........M.NGIV.H.................................NS..VAH.T.N.-E...TLP.A.KLQ.DAA.....CG....V.....E...T..V.......I....	4416
XM_041409503.1	4160	.........M.NGIV.H.................................NS..VAH.T.N.-E...TLP.A.KLQ.DAA.....CG....V.....E...T..V.......I....	4507
XM_041409492.1	4174	.........M.NGIV.H.................................NS..VAH.T.N.-E...TLP.A.KLQ.DAA.....CG....V.....E...T..V.......I....	4521
XM_041462682.1	4066	.........M.NGIV.H.................................NS..IAH.T.N.-E...TLP.A.KLQ.DAA.....CG....V.....E...T..V.......I....	4413
XM_041462681.1	4157	.........M.NGIV.H.................................NS..IAH.T.N.-E...TLP.A.KLQ.DAA.....CG....V.....E...T..V.......I....	4504
XM_041462680.1	4171	.........M.NGIV.H.................................NS..IAH.T.N.-E...TLP.A.KLQ.DAA.....CG....V.....E...T..V.......I....	4518
XM_007939578.2	3697	.........M.NGIV............I......................NI.RA.....N.M....V...S.......SR.G....VSRTP..R..E.AEL...G.....S..R..	4047
XM_038150827.1	4122	.........M.NGIV.H.................................NSE.IAH.T.N.-E...ILP.A.KLQ.DAG.....C.....V.....E...T..V.......I....	4469
XM_038150826.1	4153	.........M.NGIV.H.................................NSE.IAH.T.N.-E...ILP.A.KLQ.DAG.....C.....V.....E...T..V.......I....	4500
XM_038150825.1	4227	.........M.NGIV.H.................................NSE.IAH.T.N.-E...ILP.A.KLQ.DAG.....C.....V.....E...T..V.......I....	4574
XM_018916139.2	3751	.........M.NGIV.H.................................NS..IAH.TE..-E...VLP.A.KLQ.DAA.....CG....V.....E...T..V.......I....	4098
XM_009091985.3	3856	.........M.NGIV.H.................................NS..IAH.TE..-E...VLP.A.KLQ.DAA.....CG....V.....E...T..V.......I....	4203
XM_031595271.1	3806	.........M.NGIV.H.................................NS..IAH.T.N.-ET...LP.S.KLQ..ST.....C.....V..T..E.A.T..V.......I....	4153
XM_006130610.3	3844	.........M.SG.V.H..................................S....Q...NNLEH..MLP.Y.KLQ..A...DD.......VK.-.YE.A.T..V.......I....	4191
XM_006130609.3	3847	.........M.SG.V.H..................................S....Q...NNLEH..MLP.Y.KLQ..A...DD.......VK.-.YE.A.T..V.......I....	4194
HG999696.1	12555382	.........M.NGIV.H.................................NS..IAH.T.N.-ET...LP.S.KLQ..ST.....C.....V..T..E.A.T..V.......I....	12555035

XM_015877394.2	3711	.........M.NGIV.H.................................SS..VAH.T.N.-ET...PP.S.KLQ..ST.....C.....V..T..E.A.T..V.......I....	4058
XM_015877395.2	3835	.........M.NGIV.H.................................SS..VAH.T.N.-ET...PP.S.KLQ..ST.....C.....V..T..E.A.T..V.......I....	4182
XM_013939880.1	3696	.........M.NGIV.H.................................NC..IAH.T.N.-ET..VLP.S.KLQ..SL.....C.....V..T..E.A.T..V.......I....	4043
XM_010148043.1	3448	.........M.NGIV.H.................................SS...AH.T.N.-ET..VLP.A.KLQ.DA......C.....A..T..E.A.A..V............	3795
LK068589.1	812473	.........M.NGIV.H.................................NC..IAH.T.N.-ET..VLP.S.KLQ..SL.....C.....V..T..E.A.T..V.......I....	812820
XM_017741270.1	3453	.........M.NGIV.H.................................NS..IAH.T.N.-E...VLP.A.KLQ.DA......C.....T.....E.A.T..VD......I....	3800
XM_039560006.1	3819	.........M.NGIV.H.................................NS..IAH.T.N.-E...VLP.A.KLQ.DA......C.....T.....E.A.T..VD......I....	4166
XM_010392585.4	3924	.........M.NGIV.H.................................NS..IAH.T.N.-E...VLP.A.KLQ.DA......C.....T.....E.A.T..VD......I....	4271
XM_032125497.1	3819	.........M.NGIV.H.................................NS..IAH.T.N.-E...VLP.A.KLQ.DA......C.....T.....E.A.T..VD......I....	4166
XM_032125496.1	3924	.........M.NGIV.H.................................NS..IAH.T.N.-E...VLP.A.KLQ.DA......C.....T.....E.A.T..VD......I....	4271
XM_033073851.1	3743	.........M.NGIV.H.................................NS..IAH.T...-E...ILPPA.KLE.DAA.....C.....V.....E.A.T..V.......I....	4090
XM_033073850.1	3848	.........M.NGIV.H.................................NS..IAH.T...-E...ILPPA.KLE.DAA.....C.....V.....E.A.T..V.......I....	4195
XR_006163067.1	3848	.........M.NGIV.H.................................NS..IAH.T...-E...ILPPA.KLE.DAA.....C.....V.....E.A.T..V.......I....	4195
XR_004419546.2	3848	.........M.NGIV.H.................................NS..IAH.T...-E...ILPPA.KLE.DAA.....C.....V.....E.A.T..V.......I....	4195
XR_004419547.1	3848	.........M.NGIV.H.................................NS..IAH.T...-E...ILPPA.KLE.DAA.....C.....V.....E.A.T..V.......I....	4195
XM_036738071.1	4365	.........M.SGIV.H................................HNS....Y...GN.....VYP.S.KAR.D.......C........S..E.VEA..........I....	4715
XM_036738069.1	4368	.........M.SGIV.H................................HNS....Y...GN.....VYP.S.KAR.D.......C........S..E.VEA..........I....	4718
XM_010719366.3	1212	.........M.NGIV.H.................................NS..IAH.T.N.-ET...LP.S.KLQ..ST.....C.....V..TN.E.A.T..V.......I....	1559
LR812120.1	13379230	.........M.NG.V.H.................................NS.TIGH.TE..-E...TLP.A.KLQ.DAA.....C.....V.....E.A.T..V.......I....	13378883
XM_014892129.1	3607	.........M.NGIV.H.................................NS..VAH.TE..-E...ILP.A.KLQ.DAA.....C.....VD....E.A.T..I.......I....	3954
XM_014892128.1	3802	.........M.NGIV.H.................................NS..VAH.TE..-E...ILP.A.KLQ.DAA.....C.....VD....E.A.T..I.......I....	4149
XM_042037711.1	3825	.........M.NGIV.H.................................NS..IAH.T.N.-E...VLP.A.KLQ.DV......C.....T.....E.A.T..VD......I....	4172
XM_042037710.1	3930	.........M.NGIV.H.................................NS..IAH.T.N.-E...VLP.A.KLQ.DV......C.....T.....E.A.T..VD......I....	4277
XM_026065282.1	3307	.........M.SGIV.H.................................NC..IAH.T.N.-ET..VLP.S.KLQ..SL.....C.....V..T..E.A.T..V.......I....	3654
XM_026065281.1	3723	.........M.SGIV.H.................................NC..IAH.T.N.-ET..VLP.S.KLQ..SL.....C.....V..T..E.A.T..V.......I....	4070
XM_026065280.1	3726	.........M.SGIV.H.................................NC..IAH.T.N.-ET..VLP.S.KLQ..SL.....C.....V..T..E.A.T..V.......I....	4073
XM_026065279.1	3747	.........M.SGIV.H.................................NC..IAH.T.N.-ET..VLP.S.KLQ..SL.....C.....V..T..E.A.T..V.......I....	4094
XM_026065278.1	3750	.........M.SGIV.H.................................NC..IAH.T.N.-ET..VLP.S.KLQ..SL.....C.....V..T..E.A.T..V.......I....	4097
XM_026065277.1	3831	.........M.SGIV.H.................................NC..IAH.T.N.-ET..VLP.S.KLQ..SL.....C.....V..T..E.A.T..V.......I....	4178
XM_026065276.1	3834	.........M.SGIV.H.................................NC..IAH.T.N.-ET..VLP.S.KLQ..SL.....C.....V..T..E.A.T..V.......I....	4181
XM_010309937.1	1189	.........M.NGIV.H.................................NS..IAH.T.N.-ET..ILP.T.KLQ.DV.R....C.....A..T..E.A.T..V.......I....	1536
XM_009979365.1	3688	...........NGIV.H.................................NS..VAH.T.N.-E...TLP.T.KLQ.DV.R....C.....A..T..E.A.S..V.......I....	4035
XM_035340044.1	3778	.........M.NGIV.H.................................NS..IAH.T.N.-ET..ILS.S.KLQ.DSM.....C.....V..T..E.A.T..V.......I....	4125
XM_035340043.1	3784	.........M.NGIV.H.................................NS..IAH.T.N.-ET..ILS.S.KLQ.DSM.....C.....V..T..E.A.T..V.......I....	4131
XM_035340041.1	3882	.........M.NGIV.H.................................NS..IAH.T.N.-ET..ILS.S.KLQ.DSM.....C.....V..T..E.A.T..V.......I....	4229
XM_010117180.1	1998	.........M.NGIV.H.................................NS.N.AH.T.N.-EP...LP.A.KLQ.DVA.....C.....T..T..E.A.S..........I....	2345
XM_027882778.1	3774	.........M.NGIV.H.................................NS.N.AH.T.N.-EN..IPP.A.KLQQ.AA...........T..P..E.A.T..V.......I....	4121
XM_027882777.1	3879	.........M.NGIV.H.................................NS.N.AH.T.N.-EN..IPP.A.KLQQ.AA...........T..P..E.A.T..V.......I....	4226
XM_009923288.1	1998	.........M.NGIV.H.................................NS..IAH.T.N.-ET..ILP.A.KLQ.DA......C.....A..T..E.M.T..V.......I....	2345
XM_021411506.1	3702	.........M.NGIV.H.................................NS..VAH.T.N.-ET..SLP.S.KLQ..ST.....C.....A..T..E.A.T..V.......I....	4049
XM_021411505.1	3807	.........M.NGIV.H.................................NS..VAH.T.N.-ET..SLP.S.KLQ..ST.....C.....A..T..E.A.T..V.......I....	4154
XM_040078717.1	3736	.........M.NGIV.H.................................NS..IAH.TEN.-E...ILP.A.KLQ.DAA.....C.....V.....E.A.T..VD......I....	4083
XM_040078716.1	3841	.........M.NGIV.H.................................NS..IAH.TEN.-E...ILP.A.KLQ.DAA.....C.....V.....E.A.T..VD......I....	4188
XM_009513803.1	1825	.........M.NGIV.H.................................NS..IAH.T.N.-ETR.I.P.T.KLQ.DA.Q....C.....T..TG.E.A.T..V.......I....	2172
XM_027796197.1	3657	.........M.NGIV.H.................................NS..VAH.T.N.-E.E.ILP.A.KLQ.DA......C....TV..T..E.A.T..V.......I....	4004
XM_005244086.3	3762	.........M.NGIV.H.................................NS..VAH.T.N.-E.E.ILP.A.KLQ.DA......C....TV..T..E.A.T..V.......I....	4109
XM_026106500.1	3664	.........M.NGIV.H.................................NS..IAH.T.N.-ET..ILP.S.KLQ.DSL.....C.....V..T..E.A.T..V.......I....	4011
XM_026106499.1	3769	.........M.NGIV.H.................................NS..IAH.T.N.-ET..ILP.S.KLQ.DSL.....C.....V..T..E.A.T..V.......I....	4116
XM_035549094.1	3693	.........M.NGIV.H.................................NS..IAH.T.N.-ET..ILP.S.KLQ.DSM.....C.....V..T..E.A.T..V.......I....	4040
XM_032197488.1	3711	.........M.NGIV.H.................................NS..IAH.T.N.-ET..ILP.S.KLQ.DSM.....C.....V..T..E.A.T..V.......I....	4058
XM_035549093.1	3811	.........M.NGIV.H.................................NS..IAH.T.N.-ET..ILP.S.KLQ.DSM.....C.....V..T..E.A.T..V.......I....	4158
XM_038186691.1	4230	.........M.NGIV.H.................................NS..IAH.T.N.-ET..ILP.S.KLQ.DSM.....C.....V..T..E.A.T..V.......I....	4577
XM_038186690.1	4335	.........M.NGIV.H.................................NS..IAH.T.N.-ET..ILP.S.KLQ.DSM.....C.....V..T..E.A.T..V.......I....	4682
XM_040574105.1	3749	.........M.NGIV.H.................................NS..IAH.T.N.-ET..ILP.S.KLQ.DSM.....C.....V..T..E.A.T..V.......I....	4096

XM_040574104.1	3852	.........M.NGIV.H.................................NS..IAH.T.N.-ET..ILP.S.KLQ.DSM.....C.....V..T..E.A.T..V.......I....	4199
LS423625.1	11554898	.........M.NGIV.H.................................NS..IAH.T.N.-ET..ILP.S.KLQ.DSM.....C.....V..T..E.A.T..V.......I....	11554551
XM_005436811.2	3487	.........M.NGIV.H.................................NS..VAH.T.N.-E.E.ILP.A.KLQ.DA......C....TV..T..E.A.T..V.......I....	3834
XM_037382598.1	4018	.........M.NGIV.H.................................NS..VAH.T.N.-E.E.ILP.A.KLQ.DA......C....TV..T..E.A.T..V.......I....	4365
XM_037382597.1	4122	.........M.NGIV.H.................................NS..VAH.T.N.-E.E.ILP.A.KLQ.DA......C....TV..T..E.A.T..V.......I....	4469
XR_005931466.1	3897	.........M.NGIV.H.................................NS..IAH.T.N.-ET..ILP.A.KLQ.DA......C.....A..T..E.A.T..V.......I....	4244
XR_005931465.1	3897	.........M.NGIV.H.................................NS..IAH.T.N.-ET..ILP.A.KLQ.DA......C.....A..T..E.A.T..V.......I....	4244
XR_003921595.2	3897	.........M.NGIV.H.................................NS..IAH.T.N.-ET..ILP.A.KLQ.DA......C.....A..T..E.A.T..V.......I....	4244
XM_030002477.2	3786	.........M.NGIV.H.................................NS..IAH.T.N.-ET..ILP.A.KLQ.DA......C.....A..T..E.A.T..V.......I....	4133
XM_030002476.2	3855	.........M.NGIV.H.................................NS..IAH.T.N.-ET..ILP.A.KLQ.DA......C.....A..T..E.A.T..V.......I....	4202
XM_030002475.2	3897	.........M.NGIV.H.................................NS..IAH.T.N.-ET..ILP.A.KLQ.DA......C.....A..T..E.A.T..V.......I....	4244
LR606205.1	17325694	.........M.NGIV.H.................................NS..IAH.T.N.-ET..ILP.A.KLQ.DA......C.....A..T..E.A.T..V.......I....	17325347
XM_010578353.1	3720	.........M.NGIV.H.................................NS..IAH.T.N.-ET..ILP.A.KLQ.DA......C.....A..T..E.M.T..V.......I....	4067
XM_010578352.1	3789	.........M.NGIV.H.................................NS..IAH.T.N.-ET..ILP.A.KLQ.DA......C.....A..T..E.M.T..V.......I....	4136
XM_010578351.1	3831	.........M.NGIV.H.................................NS..IAH.T.N.-ET..ILP.A.KLQ.DA......C.....A..T..E.M.T..V.......I....	4178
XM_032703900.1	3767	.........M.NGIV.H.................................NS.NIAH.T.N.-EN..IPP.A.KLQ.DA.....K......A..S..E.A.T..V.......I....	4114
XM_027654863.1	3772	.........M.NGIV.H.................................NS.NIAH.T.N.-EN..IPP.A.KLQ.DA.....K......A..S..E.A.T..V.......I....	4119
XM_032703899.1	3872	.........M.NGIV.H.................................NS.NIAH.T.N.-EN..IPP.A.KLQ.DA.....K......A..S..E.A.T..V.......I....	4219
XM_027654862.1	3877	.........M.NGIV.H.................................NS.NIAH.T.N.-EN..IPP.A.KLQ.DA.....K......A..S..E.A.T..V.......I....	4224
XM_010286694.1	3706	.........M.NGIV.H.................................NS..IAH.T.N.-ET..ILP.A.KLQ.DA......C.....A..T..E.A.T..V.......I....	4053
XM_027683949.1	3767	.........M.NGIV.H.................................NS.N.AH.T.N.-EN..IPP.A.KLQ.DA......C.....A..S..E.A.T..V.......I....	4114
XM_027683948.1	3872	.........M.NGIV.H.................................NS.N.AH.T.N.-EN..IPP.A.KLQ.DA......C.....A..S..E.A.T..V.......I....	4219
XM_013176791.1	3487	.........M.NGIV.H.................................NS..IAH.T.N.-EN..ILP.S.KLQ.DSM.....C.....V..T..E.A.T..V.......I....	3834
XM_010216767.1	3475	.........M.NGIV.H.................................NS...AH.T.N.-ET..ILP.S.KLQRDAL.V...C.....V..T..E.A.T..V.......I....	3822
XM_009868494.1	3367	.........M.NGIV.H.................................NS...AQ.T...-ET..ILP.A.KLQ.DV.....KC.....A..T..E.A.P..V.......I....	3714
XM_039731991.1	3834	.........M.NGIV.H.................................NS..IAH.T...-E...ILP.A.KLQ.DAA.....C...Q.V.....E.-.T..V.......I....	4178
XM_039731990.1	3939	.........M.NGIV.H.................................NS..IAH.T...-E...ILP.A.KLQ.DAA.....C...Q.V.....E.-.T..V.......I....	4283
XM_030484995.1	4371	.........M.NGIV.H.................................NS..IAH.T.N.-ET..ILP.G.KLQ.DA......C.....A..A..E.A.T..V.......I....	4718
XM_030484994.1	4374	.........M.NGIV.H.................................NS..IAH.T.N.-ET..ILP.G.KLQ.DA......C.....A..A..E.A.T..V.......I....	4721
XM_030484993.1	4434	.........M.NGIV.H.................................NS..IAH.T.N.-ET..ILP.G.KLQ.DA......C.....A..A..E.A.T..V.......I....	4781
XM_030484992.1	4476	.........M.NGIV.H.................................NS..IAH.T.N.-ET..ILP.G.KLQ.DA......C.....A..A..E.A.T..V.......I....	4823
XM_014936336.1	3860	.........M.NGIV.H.................................NS...AH.T.N.-ET..ILP.T.KLQ.DAAR....C.....A..T..E.A.T..V.......I....	4207
XM_014936335.1	3964	.........M.NGIV.H.................................NS...AH.T.N.-ET..ILP.T.KLQ.DAAR....C.....A..T..E.A.T..V.......I....	4311
XM_026858701.1	3695	.........M.NGIV.H.................................NS..IAH.T.N.-ET..ILP.T.KLQ.DV......C.....A..T..E.A.T..V.......I....	4042
XM_026858700.1	3800	.........M.NGIV.H.................................NS..IAH.T.N.-ET..ILP.T.KLQ.DV......C.....A..T..E.A.T..V.......I....	4147
XM_026858699.1	3800	.........M.NGIV.H.................................NS..IAH.T.N.-ET..ILP.T.KLQ.DV......C.....A..T..E.A.T..V.......I....	4147
XM_021540801.2	3795	.........M.NGIV.H.................................NS..IAH.T.I.-E...ILP.A.KLQ.DAA.....C.....A....YE...T..V.......I....	4142
XM_021540800.2	3900	.........M.NGIV.H.................................NS..IAH.T.I.-E...ILP.A.KLQ.DAA.....C.....A....YE...T..V.......I....	4247
XM_009881961.1	3481	.........M.NGIV.H.................................NS..IAH.T.N.-ET..ILP.A.KLQ.DA.R....C.....A..T..E.A.T..V.......I....	3828
XM_010014736.1	1203	.........M.NGIV.H.................................NS..IAH.T.N.-ET..ILP.G.KLQ.DS......C.....-..T..E.V.T..V.......I....	1547
XM_026049010.1	3703	.........M.NGIV.H.................................NS...AH.T.-NLE...ILP.S.KLQ.DVL.....C........T.LE.A.T..V.......I....	4050
XM_016301896.1	3903	.........M.NG.V.H.................................NS.TIAH.TE..-E...TLP.A.KLQ..AA.....CG....V..E..E.A.TD.V.......I....	4250
XM_009324793.1	3388	.........M.NGIV.H.................................NS..IAH.T.N.-E...ILP.T.KLQ.DA.R....CT....A..T..E.A.T..V.......I....	3735
XM_009277493.2	3672	.........M.NGIV.H.................................NS..IAH.T.N.-E...ILP.T.KLQ.DA.R....CT....A..T..E.A.T..V.......I....	4019
XM_009277484.2	3678	.........M.NGIV.H.................................NS..IAH.T.N.-E...ILP.T.KLQ.DA.R....CT....A..T..E.A.T..V.......I....	4025
XM_009277476.2	3783	.........M.NGIV.H.................................NS..IAH.T.N.-E...ILP.T.KLQ.DA.R....CT....A..T..E.A.T..V.......I....	4130
XM_009936291.1	3562	.........M.NGIV.H.................................NS..IAH.T.NP-ET..ILP.T.KLQ.DA.R...W......V..T..E.A.T..V.......I....	3909
XM_005152484.3	3803	.........M.NGIV.H.................................NS..VAH.T.N.-ET..ILP.A.KLQ.DA.T....C.....A..T..E.A.T..I.......I....	4150
XM_032781649.1	3016	.........M.NG.V.H.................................NS....Q...NNLEP..ILP.Y.KLQ..S....D.C.....V-.N..EQAET..V.......I....	3363
XM_032781647.1	4365	.........M.NG.V.H.................................NS....Q...NNLEP..ILP.Y.KLQ..S....D.C.....V-.N..EQAET..V.......I....	4712
XM_032781646.1	4368	.........M.NG.V.H.................................NS....Q...NNLEP..ILP.Y.KLQ..S....D.C.....V-.N..EQAET..V.......I....	4715
XM_040589106.1	4105	.........M.NGIV.H.................................NS..VAH.TGN.-E.E.ILP.A.KLQ.DA......C....TV..T..E.A.T..V.......I....	4452
XM_040589104.1	4130	.........M.NGIV.H.................................NS..VAH.TGN.-E.E.ILP.A.KLQ.DA......C....TV..T..E.A.T..V.......I....	4477
XM_040589103.1	4209	.........M.NGIV.H.................................NS..VAH.TGN.-E.E.ILP.A.KLQ.DA......C....TV..T..E.A.T..V.......I....	4556

XM_010189842.1	1998	.........M.NGIV.H.................................NS..IAH.T...-ET..ILP.A.KLQ.DATR....CK....A..T..E.A.T..V.......I....	2345
XM_010138937.1	3688	.........M.NGIV.H.................................NSE.IAH.T.N.-EI..ILP.A.KLQQDA......C.....V..P..E.V.T..V.......I....	4035
XM_032998300.1	3786	.........M.NGIV.H.................................NS..IAH.T.N.-ET..ILP.A.KLQ.DA......C.....AG.T..E.A.T..V.......I....	4133
XM_032998299.1	3891	.........M.NGIV.H.................................NS..IAH.T.N.-ET..ILP.A.KLQ.DA......C.....AG.T..E.A.T..V.......I....	4238
XM_009900552.1	3646	.........M.NGIV.H.................................NS..IAH.T.N.-DTQ.VLP.A.KLQ.DA......C.....A..P..E.A.T..V.......I....	3993
XM_009570976.1	3523	.........M.NGIV.H.................................NS..IAH.T.N.-ET..ILP.T.KLQ.DA.R....C.....A..T..E.A.T..VD...........	3870
XM_005505623.2	3747	.........M.NGIV.H.................................NSE..AH.T.N.-ET..ILP.T.KLQ.DT.R....C...T.T..T..E.A.T..V.......I....	4094
XM_009479676.1	1998	.........M.NGIV.H.................................NS..IAH.T.N.-ET..ILP.T.KLQ.DA.R....C.....T..TG.E.A.T..V.......I....	2345
XM_006897431.1	3715	...........SGLV...................................-------T....P..R..YA.SQ..RR...R....PL.S....RS.GED..L.DT.......TT.AM	4044
XM_017808803.1	3712	.........M.NGIV.H.................................NS.NIAH.T.N.-EN..IPP.A.KLQ.DA.....KC.....A..S..E.A.T..V.......I....	4059
XM_017808802.1	3817	.........M.NGIV.H.................................NS.NIAH.T.N.-EN..IPP.A.KLQ.DA.....KC.....A..S..E.A.T..V.......I....	4164
XM_009709280.1	1998	.........M.NGIV.H.................................NS..IAH.T.N.-ET..ILP.T.KLQ.DA.....SC.....A.ET..E.A.T..V.......I....	2345
XM_010073859.1	1825	.........M.NGIV.H..........................S......NS..IAH.T.N.-ET..ILP.TQKLQ.DA.R....C.....A..S..E.A.T...D......I....	2172
XM_027750868.2	4079	.........M.NGIV.H.................................NS.NIAH.T.N.-EN..IPP.A.KLQ.DT.....KC.....A..S..E.A.T..V.......I....	4426
XM_027750867.2	4184	.........M.NGIV.H.................................NS.NIAH.T.N.-EN..IPP.A.KLQ.DT.....KC.....A..S..E.A.T..V.......I....	4531
XR_005566548.1	4184	.........M.NGIV.H.................................NS.NIAH.T.N.-EN..IPP.A.KLQ.DT.....KC.....A..S..E.A.T..V.......I....	4531
XR_005566549.1	4184	.........M.NGIV.H.................................NS.NIAH.T.N.-EN..IPP.A.KLQ.DT.....KC.....A..S..E.A.T..V.......I....	4531
XM_013005511.1	3151	.........M.NG.V...........................S.......TL.RA....EM..PP....P.S......SARV...C.LDA...ES.-----L...D.....T..EG.	3486
XR_003988182.1	3697	...........KGIV.H.................................NP.QIAH.TEN.-ET...LP.A.KLQ.DAG.....C..S..V..S..E.V.A..V.......I....	4044
XM_008496745.2	3881	...........KGIV.H.................................NP.QIAH.TEN.-ET...LP.A.KLQ.DAG.....C..S..V..S..E.V.A..V.......I....	4228
XM_009674661.1	3460	.........M.NGIV.H.................................NS..IAH.T.N.-ET..ILP.S.KLQ.DSL.....C.....A..T..E.A.P..V...A...I....	3807
XM_009808682.1	3481	.........M.NGIV.H.................................NS..IAH.T.N.-ET..ILP.A.KLQ.DAAR....C.....A..T..E.A.T..VD......I....	3828
LR594565.1	10571712	.........M.NGIV.H.................................NSE.IAH.T.N.-ET..ILP.T.KLQ.DA.R....C...T.T..T..E.A.T..V.......I....	10572059
XM_029957944.1	3466	.........M.NGIV.H.................................NSENIAH.T.N.-EN..IPP.A.KLQ.DT.....KC.....A..S..E.A.T..V.......I....	3813
XM_035889666.1	3366	.........M.NGIV.H.................................NS..IAH.T.N.-ET..ILPPT.KYQ.DAAR....C.....A..P..E.V.T..V.......I....	3713
XM_009477295.1	3700	.........M.NGIV.H..........................S......NS..IAH.T.N.-ET..ILP.T.KLQ.DA.R....C.....A..TG.E.A.A..V.......I....	4047
XM_010177295.1	1189	.........M.NGIV.H.................................NSE.IAH.T.N.-ET..ILP.A.KLQ.DAART...CG....T..T..E.A.P..V.......I....	1536
XM_009084408.1	3685	.........M.NGIV.H.................................NS..IAH.T.N.-ET..ILP.A.KLQ.DAA....KC.S...T..A..E.A.T..V.......I....	4032
OU015540.1	15899791	.........M.NGIV.H.................................NS..IAH.T.N.-ET..ILP.A.KLQ.DAAR....CG....A..T..E.A.P..VD......I....	15899444
XM_008935144.1	1195	.........M.NGIV.H.................................NS..VAH.PEN.-ET..ILPPA.KLQA.A.R....C...Q.A..A.GE.A.T..V.......I....	1542
XM_020811938.1	3158	...F.......NGIV.H.........................T.......NS...GPV.AQNSEL..GL.CF.K.S...RQSD..H....TA.QK..E.ADL...S......S.R.L	3508
XM_005306179.3	4567	.........M.NG.V.H.................................NS....Q...NNLEP..MLP.Y.KLQ..SL...D.C.....V-.N..E.AET..V.......I....	4914
XM_005306178.3	4570	.........M.NG.V.H.................................NS....Q...NNLEP..MLP.Y.KLQ..SL...D.C.....V-.N..E.AET..V.......I....	4917
XM_034783827.1	4978	.........M.NG.V.H.................................NS....Q...NNLEP..MLP.Y.KLQ..SL...D.C.....V-.N..E.AET..V.......I....	5325
XM_034783826.1	4981	.........M.NG.V.H.................................NS....Q...NNLEP..MLP.Y.KLQ..SL...D.C.....V-.N..E.AET..V.......I....	5328
XM_026659818.2	4474	.........M.NG.V.H.................................NS....Q...NNLVP..MLP.Y.KLQ..S....D.C.....V-.N..E.AET..V.......I....	4821
XM_026659816.2	4477	.........M.NG.V.H.................................NS....Q...NNLVP..MLP.Y.KLQ..S....D.C.....V-.N..E.AET..V.......I....	4824
XM_037910378.1	3059	.........M.NG.V.H.................................NS....Q...NNLEP..MLPYY.KLQ..S....D.C.....V-.N..E.AET..V.......I....	3406
XM_038419131.1	4913	.........M.NG.V.H.................................NS....Q...NNLEP..MLPYY.KLQ..S....D.C.....V-.N..E.AET..V.......I....	5260
XM_038419130.1	4916	.........M.NG.V.H.................................NS....Q...NNLEP..MLPYY.KLQ..S....D.C.....V-.N..E.AET..V.......I....	5263
XM_037910377.1	4971	.........M.NG.V.H.................................NS....Q...NNLEP..MLPYY.KLQ..S....D.C.....V-.N..E.AET..V.......I....	5318
XM_037910376.1	4976	.........M.NG.V.H.................................NS....Q...NNLEP..MLPYY.KLQ..S....D.C.....V-.N..E.AET..V.......I....	5323
XM_039491148.1	4937	.........M.NG.V.H.................................NS....Q...NNLEP..MLP.Y.KLQ.DS....D.CK....V-.N..E.AET..V.......I....	5284
XM_039491147.1	4940	.........M.NG.V.H.................................NS....Q...NNLEP..MLP.Y.KLQ.DS....D.CK....V-.N..E.AET..V.......I....	5287
XM_030579184.1	3020	.........M.SG.V.H............................-..I.NS....Q...NNLEP..MLP.Y.KLQ..S....D.C.....V-.N..E.AET..V.......I....	3364
XM_030579180.1	4373	.........M.SG.V.H............................-..I.NS....Q...NNLEP..MLP.Y.KLQ..S....D.C.....V-.N..E.AET..V.......I....	4717
XM_030579179.1	4376	.........M.SG.V.H............................-..I.NS....Q...NNLEP..MLP.Y.KLQ..S....D.C.....V-.N..E.AET..V.......I....	4720
XM_033166506.1	3973	...F.....M.NGIV.H.........................T.......NL..MPHV..HNLEL.GGLSCF.NLP..T..P.DGR....SV..N..K.A.PG.T.......N.R..	4323
XM_042437333.1	4029	.........M.NG.V.H.........................T.......NS..MAHA.EHNVEI..GLPCF.KLP..L..PDD.H.L..GR..CNGE.P.P..........S.R..	4379
XM_035131579.1	3952	...F.....M.NGIV.H.........................T.......NL..MPHV..HNLEL.GGLSCF.NLP..T..P.DGR....SV..N..K.A.PG.TS..A...N.Q..	4302
XM_015409888.1	3829	.......R...SG.V.H.........................TG.L..G.NS..APHAH.-----HN.EFL..KLP.....LDD.H...---..K..A.A.L..........S.R..	4161

\
|
YF

```
XM_009995478.1  3694      ..........NTG.V.H.K...................................NS.QAAH.----.EP.GS.STKFK--.G....QK.---S..S..T.SE.A.T..V..........L  4017
XM_028706024.1  4235      ...F.....M.NGIV.H....................T.......NL..MPHV..HNLEL.GGLPCF.NLP..T..P.DGR....SV..N..K.A---.TS......N.R..  4576
XM_028706023.1  4330      ...F.....M.NGIV.H....................T.......NL..MPHV..HNLEL.GGLPCF.NLP..T..P.DGR....SV..N..K.A---.TS......N.R..  4671
XM_026704624.1  3499      ...F.....M.SG.V.H....................T.......NL..ML----PD.EL..LLPRF.KFP..LI.LDS.PQ..A.G..G.GE.A.P.........S.R..  3837
XM_026683270.1  3616      ...F.....M.SG.V.H....................T.......NL..ML----PD.EL..LLPRF.KFP..LI.LDS.PQ..A.G..G.GE.A.P.........S.R..  3954
XM_029283350.1  3655      ...F.....M.SG.V.H....................T.......NL..MP----PDLEL..LLPRF.KFP..L..LDS.PQ..A.G..G..E.ARP.........S.R..  3993
XM_007433866.2  3037      ...F.....M.SG.V.Y....................T..A...NL..IP----TDAEL..LPRFK.F.-..L..LDN.LQ..I.G..S..E.A.PD........S.R..  3372
XM_029576521.1  3933      ...F.....M.NGIV.H....................TS......SS..HFHC.ENA-EM.EVL.VSQKLRRD.A.S...S.LS....-.G.G...M.DVD......S.ERL  4277
XM_029576520.1  3936      ...F.....M.NGIV.H....................TS......SS..HFHC.ENA-EM.EVL.VSQKLRRD.A.S...S.LS....-.G.G...M.DVD......S.ERL  4280
LR584400.1      44885117  ...F.....M.NGIV.H....................TS......SS..HFHC.ENA-EM.EVL.VSQKLRRD.A.S...S.LS....-.G.G...M.DVD......S.ERL  44884773
XM_039360673.1  3766      ...F.....M.SGGV.H....................T.......NL..MP----PDLEL..LLPRF.KFP..L..LDR.PQ..A.G..G..E.ARP.........S.R..  4104
XM_030212364.1  3871      ...F.....M.NGIV.H....................TS......SS..S.HC.E-N.EMRE.L.VSQKLHRD.A.K..QS.LC.T..-.GFG.M.M...D......S.EKL  4215
XM_030212363.1  3874      ...F.....M.NGIV.H....................TS......SS..S.HC.E-N.EMRE.L.VSQKLHRD.A.K..QS.LC.T..-.GFG.M.M...D......S.EKL  4218
XM_034441390.1  3478      ...F.....M.SG.V.H....................T.......NL..MP----PDIEL..LLPRF.KFP..L..LDS.PQ..A.G..GV.E.A.PG.V.......S.R..  3816
XM_032230974.1  3673      ...F.....M.SG.V.H....................T.......NL..VP----PD.EL..LLPRF.KFP..L..LDS.PQ.DA.G..GG.E.A.PG.........S.W..  4011
XM_033964018.1  3938      ...F.....M.NGIV.H....................TS......NS..-.FHCSEN.EMREVL.VSQKLHRD.A.K...S.LCR...-.SFG.M.M...D.....KS.EKL  4282
XM_033964017.1  3990      ...F.....M.NGIV.H....................TS......NS..-.FHCSEN.EMREVL.VSQKLHRD.A.K...S.LCR...-.SFG.M.M...D.....KS.EKL  4334
XM_033964016.1  4096      ...F.....M.NGIV.H....................TS......NS..-.FHCSEN.EMREVL.VSQKLHRD.A.K...S.LCR...-.SFG.M.M...D.....KS.EKL  4440
XM_014069689.1  3310      ...F.....MESG.V.H....................T.......NL..VP----PD.EL..LLPRF.KLP..L..LDS.PQ.DA.G..GG.E.A.PG.........S.W..  3648
XM_004691783.2  3745      .......R.S.SGLV.......................S......GS.RA......GVP.R..PPAAR.L.RD.GRSK                                    3993
XM_042437334.1  4181      ..........M.NG.V.H....................T.......NS..MAHA.EHNVEI..GLPCF.KLP..L..PDD.H.L..GR..                        4462
XM_038819434.1  3874      ........S.NSGI..S..H.V...................L......LSIS.RSGRY.E-D.E...SLSARQKVK.....-.AD.KV.-------.V.P---..TD.....QI.    4182
XM_038819433.1  3877      ........S.NSGI..S..H.V...................L......LSIS.RSGRY.E-D.E...SLSARQKVK.....-.AD.KV.-------.V.P---..TD.....QI.    4185
XM_007893612.2  3865      ........S.NSGI..S..HTV...................LG.....LLIS....-H.SGN.E...LLYTHRKVK..Q..-.AGSKVDRYVGEE.GSR.--Q.ID.....QI.    4194
XM_008123237.2  3913      .....F...M.NG.V.H........................LSG.....PNA.RALH.REGP.GLRKG.--..Q..LGKGGD..K.GS.CQEGDN.GPPEEE..........S.R..  4287
                                                                              \               \
                                                                              |               |
                                                                             AVE           EPVKPDG

XM_014485369.1  5148      .....F..YM.NGIM......V...................L.SM...L.SS..S.HC...G-EV..LLS.SQKLMRDLANIPDKRAVN.KVDD---EDYAD.D.D.....K..E.L  5486
XM_014485368.1  5184      .....F..YM.NGIM......V...................L.SM...L.SS..S.HC...G-EV..LLS.SQKLMRDLANIPDKRAVN.KVDD---EDYAD.D.D.....K..E.L  5522
XM_005990661.2  5190      .....F..YM.NGIM......V...................L.SM...L.SS..S.HC...G-EV..LLS.SQKLMRDLANIPDKRAVN.KVDD---EDYAD.D.D.....K..E.L  5528
XM_033040652.1  3863      ........S.NSGI..S..H.V...................L.G......IM..SAQC..N.-ED..LLCALQK------..T--------.QV.-EA.SKM.D..MD.....QI.    4171
                                                                                                                      \
                                                                                                                      |
                                                                                                                     EDP

XM_033040651.1  3863      ........S.NSGI..S..H.V...................L.G......IM..SAQC..N.-ED..LLCALQK------..T--------.QV.-EA.SKM.D..MD.....QI.    4171
                                                                                                                      \
                                                                                                                      |
                                                                                                                     EDP

XM_041207136.1  3848      ........S.NSGI..S..H.V...................L......LPIS.RSGRY.N-N.E...S-..ARQKV.KEQAK.VD.KV.-------.E.P---..TD...N.QI     4153
XM_041207133.1  3851      ........S.NSGI..S..H.V...................L......LPIS.RSGRY.N-N.E...S-..ARQKV.KEQAK.VD.KV.-------.E.P---..TD...N.QI     4156
XM_020513088.1  1130      ........S.NSGI..S..H.V...................L......LPVL.RSGY-YPAN.E.N.SSSACQKVT.VQI.EAD-----DK.GD..P------..MD.....QI.SQL  1447
XM_020513087.1  1133      ........S.NSGI..S..H.V...................L......LPVL.RSGY-YPAN.E.N.SSSACQKVT.VQI.EAD-----DK.GD..P------..MD.....QI.SQL  1450
XM_018561168.1  4267      ..........ENGLV.H.........................SS..T-----------.GKGAAESGGGETAR..E------...D...D.G.                     4497
                                 \
                                 |
                                 G

XM_041277040.1  5211      ...F.F..SV.SGMV......V...................L..M...AHGL.R.AAAAMEKLDV..S....Q..LPAQAL.TTAT.NLH.E.EEGEE.EEE...D.....KI.E.L  5618
                                                                      \      \                \  \  \
                                                                      |      |                |  |  |
                                                                     GG   PPQR               SG  RAAPFAVS
                                                                                                    |
                                                                                                   GNT

XM_041277039.1  5337      ...F.F..SV.SGMV......V...................L..M...AHGL.R.AAAAMEKLDV..S....Q..LPAQAL.TTAT.NLH.E.EEGEE.EEE...D.....KI.E.L  5744
```

```
                                                                           \     \            \ \ \
                                                                           |     |            | | |
                                                                          GG  PPQR           SG RAAPFAVS
                                                                                                  |
                                                                                                GNT
XM_034031186.2  2236      ...F.F..SV.SGMV......V..................L..M...                                                   2379
XM_040356568.1  3740      .....F.....NGLV.H.....................SS..T----------.GKGAAESGSGETAR..E-----.S.D...D.G.          3970
                                       \
                                       |
                                       G
XM_030579183.1  4288                              ..............-..I.NS....Q...NNLEP..MLP.Y.KLQ..S....D.C.....VK        4491
XM_015945597.1  4996      ...F.....S.SGLV..Y...V..E..A...............SM..AITM.PSA.T..SNTS.T.E.DICERC.                    5253
                                                                         \              \
                                                                         |              |
                                                                  ASSGTASS            AGL
XM_015945596.1  5017      ...F.....S.SGLV..Y...V..E..A...............SM..AITM.PSA.T..SNTS.T.E.DICERC.                    5274
                                                                         \              \
                                                                         |              |
                                                                  ASSGTASS            AGL
CP032589.1      2722212   ...F.....S.SGPV..Y...V..E..A...............SM..TTSM.PSA.A..STT.HE.E.DSRER..ST.                 2721946
                                                                            \            \
                                                                            |            |
                                                                     VNASSTNT           GLS
XM_034052840.2  4647      ...F.F..SV.SGMV......V....................E.L..M...                                              4790
XM_015359942.1  5292      ...F.F..SV.SGMV......V.......................GV...                                              5435
XM_015359940.1  5463      ...F.F..SV.SGMV......V.......................GV...                                              5606
LR991642.1      16103443  ...F.....S.SGPV..Y...V..E..A...............SM..ATSMAAS.T                                         16103610
XM_041229029.1  5384      ...F.F..SV.SGLV......V....................E.L..M..T                                              5527
XM_041229028.1  5511      ...F.F..SV.SGLV......V....................E.L..M..T                                              5654
XM_019897423.1  4956      ...F.....G.SGPV..Y...V..E..A...............SM...ASM.PSA.A..SAK.                                5156
                                                                           \
                                                                           |
                                                                        AASG
XM_041579200.1  4598      .....F.....NGLV.H...C...................LSS..T----------.GKGAVESGSGEAGR..E-----.P.....D.G.         4828
                                       \
                                       |
                                       G
XM_040441811.1  4595      ...........NGLV.H.....................SS..                                                        4735
                                       \
                                       |
                                       G
XM_016293776.1  3688      ........NS.SGPV..Y...V..E...................M..A                                                 3831
XM_016495741.1  4417      ........NS.SGPV..Y...V..E...................M..A                                                 4560
XM_028412431.1  5337      ...F.....S.SGPV..Y...V..E..A...............SM..T                                                 5480
LR131980.1      5211103   ...F.....S.SGPV..Y...V..E..A...............SM..T                                                 5211246
BC128643.1      4247      .....F.....NGLV.H.....................SS..TA                                                      4393
                                       \
                                       |
                                       G
XM_031892851.1  4670      .....F.....NGLV.H.....................SS..TA                                                      4816
                                       \
                                       |
                                       G
XM_037688378.1  3826      ...F.....S.SGPV..Y...V..E..A...............SM..T                                                 3969
```

```
XM_028814624.1  4764      .....F..SM.NGMV.H....V..E....................EM..A.                    4910
XM_026239986.1  5393      ........SS.RGPV..Y...V..E..A..................M..A                    5536
XM_042717303.1  4303      ........SS.SGPV..Y...V..E..A..................M..AAN                  4452
XM_042717313.1  5209      ........SS.SGPV..Y...V..E..A..................M..AAN                  5358
XM_042717318.1  5270      ........SS.SGPV..Y...V..E..A..................M..AAN                  5419
XM_042717327.1  5348      ........SS.SGPV..Y...V..E..A..................M..AAN                  5497
XM_042717299.1  5422      ........SS.SGPV..Y...V..E..A..................M..AAN                  5571
XM_016235913.1  2428      ........SS.SGHV..Y...V..E..A..................M..A                    2571
XM_016525687.1  3970      ........SS.SGPV..Y...V..E..A..................M..A                    4113
LR812065.1      10330971  ........SA.SGPV..Y...V..E..A..................M..T                    10330828
XM_042719590.1  5394      ........SS.SGPV..Y...V..E..A..................M..A                    5537
XM_026215784.1  4222      ........SS.SGPV..Y...V..E..A..................M..A                    4365
XM_026215775.1  4317      ........SS.SGPV..Y...V..E..A..................M..A                    4460
LR812521.1      17182103  ........SA.SGPV..Y...V..E..A..................M..T                    17181960
XM_015391219.1  5123      ...F....SS.SGPV..Y...V..E..A..................M..TTMM.PSA.T..SNTS.M..SDIHERH.   5380
                                                                            \            \
                                                                            |            |
                                                                     SKNANSTK          SGL
XM_034189784.1  4220      ...F....SS.SGTV......V..E..A..............L.TM...ST.TMVP.MSTC...NTGLG.   4444
                                                                          \
                                                                          |
                                                                     TTSMTA
LR722981.1      49381622  ...F....SS.SGTV......V..E..A..............L.TM...ST.TMVP.MSTC...NTGLG.   49381846
                                                                          \
                                                                          |
                                                                     TTSMTA
XM_009299168.3  1990      ........SA.SGPV..Y...V..E..A..................M..T                    2133
XM_021472892.1  1990      ........SA.SGPV..Y...V..E..A..................M..T                    2133
LR812517.1      9690060   ........SA.SGPV..Y...V..E..A..................M..T                    9689917
CP068737.1      9761081   ........SA.SGPV..Y...V..E..A..................M..T                    9760938
CR339059.15     77159     ........SA.SGPV..Y...V..E..A..................M..T                    77016
LR812596.1      6578484   ........SA.SGPV..Y...V..E..A..................M..T                    6578627
LR812546.1      5872495   ........SA.SGPV..Y...V..E..A..................M..T                    5872352
LR812571.1      8959097   ........SA.SGPV..Y...V..E..A..................M..T                    8958954
LR812040.1      10638117  ........SA.SGPV..Y...V..E..A..................M..T                    10637974
XM_030789704.1  3766      ...F....SA.SGPV..Y...V..E..A.......................A                  3909
LR697118.1      15598224  ...F....SA.SGPV..Y...V..E..A.......................A                  15598367
XM_014983612.1  5407      ...F....SS.SGPV..Y...V..E..A................SM..TTMM.PSA.I..SNT..M..SDI.   5652
                                                                            \             \
                                                                            |             |
                                                                     SKNGNSTK           GLS
XM_014983611.1  5428      ...F....SS.SGPV..Y...V..E..A................SM..TTMM.PSA.I..SNT..M..SDI.   5673
                                                                            \             \
                                                                            |             |
                                                                     SKNGNSTK           GLS
XM_039775821.1  4488      .....F..SM.NGMV.H....V..E....................EM..A                     4631
XM_018743020.2  5880      ...F.F..S..SGPV..Y...V..E..A.................G.M...                    6023
LR584068.1      31749773  ...F.F..S..SGPV..Y...V..E..A.................G.M...                    31749630
XR_004896137.1  5461      ...F....SS.SGPV..Y...V..E....................SM..T                     5604
XM_035996491.1  5437      ...F....SS.SGPV..Y...V..E....................SM..T                     5580
XM_031312360.2  5461      ...F....SS.SGPV..Y...V..E....................SM..T                     5604
XM_028599250.1  5580      ...F....SS.SGPV..Y...V..E....................SM..T                     5723
XM_034895193.1  5566      ...F....SS.SGPV..Y...V..E....................SM..TTSMIPSA.A..STT.HG.E.DSHERH.ST.TSH.RQ   5850
                                                                              \           \
```

```
                                                                             |           |
                                                                      AASSTNTT         GLS
XM_034895192.1  5590      ...F....SS.SGPV..Y...V..E..................SM..TTSMIPSA.A..STT.HG.E.DSHERH.ST.TSH.RQ                                   5874
                                                                             \           \
                                                                             |           |
                                                                      AASSTNTT         GLS
XM_018680848.1  5667      ...F....SS.SGPV..Y...V..E..A...............SM..TTSM.PSA.A..STT.HE.E.DSRER..SA.                                         5933
                                                                            \            \
                                                                            |            |
                                                                     MTASSTNT          GLS
XM_026164962.1  5638      ...F....SS.SGPV..Y...V..E..A................M..TNTM.PSA.T..P.T.HV.EVDSRER..TA.TSA.                                     5916
                                                                         \              \
                                                                         |              |
                                                                  ATSTTASS            TGL
XM_016451524.1  4070      ........SS.SGPV..Y...V..E..A...............T.M..A                                                                       4213
XM_032587761.1  4717      ...F....SS.SGPV..Y...V..E..A...............SM..TTMM.PSA.I..SNT..M..SDINGR                                               4968
                                                                           \             \
                                                                           |             |
                                                                    SKNGSSTK           GLS
XM_023348708.1  4728      ...F....SS.SGPV..Y...V..E..A...............SM..TTMM.PSA.I..SNT..M..SDINGR                                               4979
                                                                           \             \
                                                                           |             |
                                                                    SKNGSSTK           GLS
XM_032587762.1  5340      ...F....SS.SGPV..Y...V..E..A...............SM..TTMM.PSA.I..SNT..M..SDINGR                                               5591
                                                                           \             \
                                                                           |             |
                                                                    SKNGSSTK           GLS
XM_032587760.1  5361      ...F....SS.SGPV..Y...V..E..A...............SM..TTMM.PSA.I..SNT..M..SDINGR                                               5612
                                                                           \             \
                                                                           |             |
                                                                    SKNGSSTK           GLS
XM_023348709.1  5350      ...F....SS.SGPV..Y...V..E..A...............SM..TTMM.PSA.I..SNT..M..SDINGR                                               5601
                                                                           \             \
                                                                           |             |
                                                                    SKNGSSTK           GLS
XM_005809294.2
5371      ...F....SS.SGPV..Y...V..E..A...............SM..TTMM.PSA.I..SNT..M..SDINGR                                               5622
                                                                           \             \
                                                                           |             |
                                                                    SKNGSSTK           GLS
XM_039824660.1  5335      ...F....SS.SGPV..Y...V..E..................SM..T                                                                        5478
XM_039686383.1  4501      ........S..SGPV..Y...V..E..A................MI                                                                          4638
XM_039686382.1  4504      ........S..SGPV..Y...V..E..A................MI                                                                          4641
XM_011603970.2  5283      ...F....SS.SGPV..Y...V..E..A...............SM..T------...NTS.V.                                                         5453
XM_029836343.1  5287      ...F....SS.SGPV..Y...V..E..A...............SM..T------...NTS.V.                                                         5457
LR584247.1      8096920   ...F....SS.SGPV..Y...V..E..A...............SM..T------...NTS.V.                                                         8096750
XM_032537334.1  4075      ...F....SS.SGPV..Y...V..E..................SM..T                                                                        4218
XM_032537333.1  5584      ...F....SS.SGPV..Y...V..E..................SM..T                                                                        5727
XM_032537332.1  5608      ...F....SS.SGPV..Y...V..E..................SM..T                                                                        5751
XM_028042491.1  4722      ...F....SN.SGPV..Y...V..E..A...............SM..TAMM.PSA.I..SNT..M..SDINGR                                               4973
                                                                           \             \
                                                                           |             |
                                                                    SKNGSSTK           GLS
XM_028042492.1  5347      ...F....SN.SGPV..Y...V..E..A...............SM..TAMM.PSA.I..SNT..M..SDINGR                                               5598
```

```
                                                                              \             \
                                                                              |             |
                                                                       SKNGSSTK           GLS
XM_028042490.1    5368       ...F....SN.SGPV..Y...V..E..A...............SM..TAMM.PSA.I..SNT..M..SDINGR                           5619
                                                                              \             \
                                                                              |             |
                                                                       SKNGSSTK           GLS
LT972185.1        18934556   ...F....SS.SGPV..Y...V..E..A...............SM..TTSI.PSA.V..STT.                                     18934344
                                                                                \
                                                                                |
                                                                         NASNTTST
XM_022752346.1    5469       ...F....SS.SGPV..Y...V..E..A...............SM..TTTM.PSA.A..STT.HE.E.GSRER..SA.                      5735
                                                                              \             \
                                                                              |             |
                                                                       GMTASITN           GLG
XM_008416707.2    5394       ...F....SS.SGPV..Y...V..E..A...............SM..TTMM.PSA.I..SNT..M..SDINVR                           5645
                                                                              \             \
                                                                              |             |
                                                                       SKNGSSIK           GLS
XM_008416706.2    5415       ...F....SS.SGPV..Y...V..E..A...............SM..TTMM.PSA.I..SNT..M..SDINVR                           5666
                                                                              \             \
                                                                              |             |
                                                                       SKNGSSIK           GLS
XM_012860685.3    5448       ...F....SS.SGPV..Y...V..E..A.............L..M..T                                                    5591
XM_016521798.1    4414       ........NS.SGPV..Y...V..E...................M.                                                      4551
LR880652.1        14903057   ...F....SS.SGPV..Y...V..E..A...............SM..TTMM.PSA.I..SNT..M..SDINVR                           14902806
                                                                              \             \
                                                                              |             |
                                                                       SKNGSSIK           GLS
XM_038270066.1    5193       ...F....SS.SGPV..Y...V..E..A................M..T                                                    5336
XM_023423302.1    5435       ...F....SS.SGPV..Y...V..E..A...............SM..TTTM.PSA.A..STT.HE.E.GSRER..SA.                      5701
                                                                              \             \
                                                                              |             |
                                                                       GVTASITN           GLG
XM_007556397.2    3546       ...F....SS.SGPV..Y...V..E..A...............SM..TTMM.PSA.I..SNT..M..SDI                              3788
                                                                              \             \
                                                                              |             |
                                                                       SKNGNSTK           GLS
XM_007556396.2    3567       ...F....SS.SGPV..Y...V..E..A...............SM..TTMM.PSA.I..SNT..M..SDI                              3809
                                                                              \             \
                                                                              |             |
                                                                       SKNGNSTK           GLS
XM_015025907.1    5400       ...F....SS.SGPV..Y...V..E..A...............SM..TTMM.PSA.I..SNT..M..SDI                              5642
                                                                              \             \
                                                                              |             |
                                                                       SKNGNSTK           GLS
XM_015025898.1    5421       ...F....SS.SGPV..Y...V..E..A...............SM..TTMM.PSA.I..SNT..M..SDI                              5663
                                                                              \             \
                                                                              |             |
                                                                       SKNGNSTK           GLS
XM_038700667.1    5408       ...F....SS.SGPV..Y...V..E..A...............SM..T------..IN.S.TNT.                                   5584
XM_020622130.1    5146       ........SS.SGPV..Y...V..E..A...............SM..T                                                    5289
LR738556.1        19252992   ...F....SS.SGPV..Y...V..E..A...............SM..ATSMASN.VVP.A.TGSSNATG..YM.------E.DT...             19252741
                                                                                 \
```

```
                                                                              |
                                                                            ASC
XM_027009150.2  5704      ...F....SG.SGLV..Y..LV..E..A...............M..T                                              5847
XM_034142904.1  3853      ...F....SS.SGPV..Y...V..E..A..............SM..ATSLIKGL...KE...ETSN....SSAQQNT.ITSQ------.Y.K..K...APDCNVG...R.        4284
                                                                             \        \       \ \
                                                                             |        |       | |
                                                              TASSTHTTVPSAGSST     AVRR   RRPSS DGPTSSANNQQLLI
XM_034142905.1  5470      ...F....SS.SGPV..Y...V..E..A..............SM..ATSLIKGL...KE...ETSN....SSAQQNT.ITSQ------.Y.K..K...APDCNVG...R.        5901
                                                                             \        \       \ \
                                                                             |        |       | |
                                                              TASSTHTTVPSAGSST     AVRR   RRPSS DGPTSSANNQQLLI
XM_019258043.2  5587      ...F....SS.SGPV..Y...V..E..A..............SM..T------..INAS.T.                                5757
XM_042504915.1  4079      ...F....SS.SGPV..Y...V..E..A..............SM..TNTM.PSA.V..ATT.HG.E.DSRER..                    4336
                                                                          \               \
                                                                          |               |
                                                                  TTSITTSS             GLS
XM_042504914.1  4166      ...F....SS.SGPV..Y...V..E..A..............SM..TNTM.PSA.V..ATT.HG.E.DSRER..                    4423
                                                                          \               \
                                                                          |               |
                                                                  TTSITTSS             GLS
XM_042504913.1  4190      ...F....SS.SGPV..Y...V..E..A..............SM..TNTM.PSA.V..ATT.HG.E.DSRER..                    4447
                                                                          \               \
                                                                          |               |
                                                                  TTSITTSS             GLS
AP022717.1      17928415  ...F....SS.SGPV..Y...V..E..A..............SM..TNTM.PSA.V..ATT.HG.E.DSRER..                    17928672
                                                                          \               \
                                                                          |               |
                                                                  TTSITTSS             GLS
XM_034594251.1  5229      ...F....SS.SGPV..Y...V..E..................SM..T                                              5372
LR699033.1      24390321  ...F....SS.SGPV..Y...V..E..A..............SM..T------..INAG.T.                                24390151
XM_034594250.1  5437      ...F....SS.SGPV..Y...V..E..................SM..T                                              5580
XM_035180984.1  5410      ...F....SS.SGPV..Y...V..E..................SM..T                                              5553
XM_011478182.3  5072      ...F....SS.SGPV..Y...V..E..A..............SM..T-------------...S.RN.N                         5242
XM_011478180.3  5176      ...F....SS.SGPV..Y...V..E..A..............SM..T-------------...S.RN.N                         5346
HF933214.1      11367288  ...F....SS.SGPV..Y...V..E..A..............SM..T-------------...S.RN.N                         11367458
CP020672.1      11278599  ...F....SS.SGPV..Y...V..E..A..............SM..T-------------...S.RN.N                         11278769
XM_031877759.1  4149      ...F....SS.SGPV..Y...V..E..A..............SM..T                                              4292
XM_010788116.1  1877      ...F....SS.SGPV..Y...V..E..A...........E...SM..ATSLIKGL...KE...ETSN....SSAQQNT.ITSQ------.Y.K..K...APDSHVG...R.        2308
                                                                             \        \       \ \
                                                                             |        |       | |
                                                              TASSTHTTVHSAGSST     AVRR   RRPSS DGPTSSANNQQLLI
XM_041956629.1  5582      ...F....SS.SGPV..Y...V..E..A.............TSM..TTSI.PSA.A..STT.GLSHEKEAER..ST.TSH.            5851
                                                                             \
                                                                             |
                                                                      NASTTTTT
XM_034219796.1  5073      ...F....SS.SGPV..Y...V..E..A...........E...SM..ATSLIKGL...KE...ETSN....SSAQQNT.ITSQ------.Y.K..K...APDSNVG...R.        5504
                                                                             \        \       \ \
                                                                             |        |       | |
                                                              TASSTHTTVHSAGSST     AVRR   RRPSS DGPTSSANNQQLLI
AP022693.1      23513363  ...F....SS.SGPV..Y...V..E..A..............SM..TTGITAS.T                                     23513530
OU342820.1      12657562  ...F....SS.SGPV..Y...V..E..A..............SM..T                                              12657705
XM_026325855.1  5543      ...F....SS.SGPV..Y...V..E..A..............SM..T                                              5686
XM_026325854.1  5567      ...F....SS.SGPV..Y...V..E..A..............SM..T                                              5710
LR535840.1      15603815  ...F....SS.SGPV..Y...V..E..A..............SM..T                                              15603958
```

```
XM_026350891.1  5214      ...F....SS.SGPV..Y...V..E..A...............SM..T    5357
XM_026350881.1  5444      ...F....SS.SGPV..Y...V..E..A...............SM..T    5587
XM_026350872.1  5553      ...F....SS.SGPV..Y...V..E..A...............SM..T    5696
LR132046.1      13633078  ...F....SS.SGPV..Y...V..E..A...............SM..T    13633221
XM_035654802.1  5645      ...F....SS.SGPV..Y...V..E..A...............SM..A    5788
XM_035654801.1  5804      ...F....SS.SGPV..Y...V..E..A...............SM..A    5947
XM_033645489.1  5561      ...F....SS.SGPV..Y...V..E..A...............SM..TTTM.PSA.A.AS.T.HGRE.DSRDR..    5818
                                                                           \             \
                                                                           |             |
                                                                    GITASSTN           GLS
XM_020647688.2  4207      ...F....SS.SGPV..Y...V..E..A...............SM..A    4350
XM_034088758.1  5130      ...F....SS.SGPV..Y...V..E..A...........E...SM..ATSLIKGL...KE...ETSN....SSAKQNT.ITSQ------.Y.K..K...APDSNVG...Q.    5561
                                                                             \        \       \ \
                                                                             |        |       | |
                                                              TASSTHTTVYSAGSST     AVRR   RRPSS DGPTSSANNQQLLI
LR792553.1      22554505  ...F....SS.SGPV..Y...V..E..A...........E...SM..ATSLIKGL...KE...ETSN....SSAKQNT.ITSQ------.Y.K..K...APDSNVG...Q.    22554936
                                                                             \        \       \ \
                                                                             |        |       | |
                                                              TASSTHTTVYSAGSST     AVRR   RRPSS DGPTSSANNQQLLI
XM_041970000.1  4467      ...F....SS.SGPV..Y...V..E..A...............SM..T    4610
XM_024272822.2  5142      ...F....SS.SGPV..Y...V..E..A...............SM..TTSL.LSA.T..SNTNNL.E.DIYGR..SP.    5408
                                                                            \           \
                                                                            |           |
                                                                     TSARSANP         RGL
CP020786.1      10850172  ...F....SS.SGPV..Y...V..E..A...............SM..T    10850315
XM_029437098.1  5536      ...F....SS.SGPV..Y...V..E..A...............SM..T    5679
XM_029437097.1  5617      ...F....SS.SGPV..Y...V..E..A...............SM..T    5760
XM_037792286.1  5437      ...F....SS.SGPV..Y...V..E..A...............SM..A    5580
LR131938.1      9669596   ...F....SS.SGPV..Y...V..E..A...............SM..T    9669739
XM_022195337.1  4878      ...F....SS.SGPV..Y...V..E..A...............SM..A    5021
XM_022195335.1  4902      ...F....SS.SGPV..Y...V..E..A...............SM..A    5045
XM_041817323.1  5311      ...F....SS.SGPV..Y...V..E..A...............SM..A    5454
XM_041817322.1  5406      ...F....SS.SGPV..Y...V..E..A...............SM..A    5549
XM_041062656.1  5223      ...F....SS.SGPV..Y...V..E..A...............SM..A    5366
XM_041062654.1  5343      ...F....SS.SGPV..Y...V..E..A...............SM..A    5486
XM_041062655.1  5348      ...F....SS.SGPV..Y...V..E..A...............SM..A    5491
XM_033971394.1  4024      ...F....SS.SGPV..Y...V..E..A...............SM..N    4167
XM_008283781.1  3850      ...F....SS.SGPV..Y...V..E..A...............SM..TNTM.PSA.T..SAT.HV.E.DSHER..    4107
                                                                         \               \
                                                                         |               |
                                                                  ATSTTASS             GIS
XM_035615608.1  3838      ...F....SS.SGPV..Y...V..E..A...............SM..T    3981
XM_035615607.1  5376      ...F....SS.SGPV..Y...V..E..A...............SM..T    5519
CP026261.1      11391712  ...F....SS.SGPV..Y...V..E..A...............SM..T    11391569
CP020628.1      11577611  ...F....SS.SGPV..Y...V..E..A...............SM..T------------...S.RN.N    11577781
XM_035957669.1  4919      ...F....SS.SGPV..Y...V..E..A...............SM..A    5062
XM_023291217.2  4943      ...F....SS.SGPV..Y...V..E..A...............SM..A    5086
XM_020101827.1  5538      ...F....SS.SGPV..Y...V..E..A...............SM..T    5681
XM_006791438.2  5620      ...F....SS.SGPV..Y...V..E..A...............SM..TNTM.PSA.T..P.T.HV.EVDSRER..TA.TSA.    5898
                                                                         \              \
                                                                         |              |
                                                                  ATSTTASS            TGL
XM_029160036.2  5236      ...F....SS.SGPV..Y...V..E..A...............SM..T    5379
XM_003450058.4  5641      ...F....SS.SGPV..Y...V..E..A...............SM..TNTM.PSA.T..P.T.HV.EVDSRER..TA.TSA.    5919
```

```
                                                                         \              \
                                                                         |              |
                                                                  ATSTTASS            TGL
XM_031741290.2  5691      ...F....SS.SGPV..Y...V..E..A...............SM..TNTM.PSA.T..P.T.HV.EVDSRER..TA.TSA.    5969
                                                                         \              \
                                                                         |              |
                                                                  ATSTTASS            TGL
XM_004559369.3  5613      ...F....SS.SGPV..Y...V..E..A...............SM..TNTM.PSA.T..P.T.HV.EVDSRER..TA.TSA.    5891
                                                                         \              \
                                                                         |              |
                                                                  ATSTTASS            TGL
XM_005744372.1  5638      ...F....SS.SGPV..Y...V..E..A...............SM..TNTM.PSA.T..P.T.HV.EVDSRER..TA.TSA.    5916
                                                                         \              \
                                                                         |              |
                                                                  ATSTTASS            TGL
LR132020.2      5495277   ...F....SS.SGPV..Y...V..E..A...............SM..T                                      5495134
XM_005943986.3  5660      ...F....SS.SGPV..Y...V..E..A...............SM..TNTM.PSA.T..P.T.HV.EVDSRER..TA.TSA.    5938
                                                                         \              \
                                                                         |              |
                                                                  ATSTTASS            TGL
XM_030089255.1  3952      ...F....SS.SGPV..Y...V..E..A...............SM..ASSM.PSA.T..SAA.GLSRVKDADR..             4197
                                                                            \
                                                                            |
                                                                      AANSSST
LR597439.1      12073953  ...F....SS.SGPV..Y...V..E..A...............SM..ASSM.PSA.T..SAA.GLSRVKDADR..             12073708
                                                                            \
                                                                            |
                                                                      AANSSST
XM_034712209.1  4860      ...F....SS.SGPV..Y...V..E..A...............SM..A                                      5003
XM_034712208.1  5420      ...F....SS.SGPV..Y...V..E..A...............SM..A                                      5563
XM_020933717.1  3751      ...F....SS.SGPV..Y...V..E..A...............SM..N                                      3894
XM_014010095.1  5317      ...F.F..SS.SGPV..Y...V..E..A...............SM..TTSMIASCTNTMVP.AS.GSSN.TGLSQT..        5550
XM_040049846.1  5623      ...F....SS.SGPV..Y...V..E..A...............SM..                                       5763
XM_008329868.3  4752      ...F....SS.RGPV..Y...V..E..A................I.HT.                                     4898
XM_008329867.3  4585      ...F....SS.RGPV..Y...V..E..A................I.HT.                                     4731
XM_008329869.3  4713      ...F....SS.RGPV..Y...V..E..A................I.HT.                                     4859
XM_025064269.1  4728      ...F....SS.RGPV..Y...V..E..A................I.HT.                                     4874
OU342727.1      13634627  ...F....SS.SGPV..Y...V..E..A...............SM..                                       13634767
XM_034540195.1  3826      ...F....SS.SGPV..Y...V..E..A...............SM..                                       3966
XM_037475977.1  5564      ...F....SS.SGPV..Y...V..E..A...............SM..T                                      5707
NM_001085833.1  4131      ....HF.....NGLV.H...C.....................SS..                                        4271
                                       \
                                       |
                                       G
XM_030059038.1  3914      ...F....SS.SGPV..Y...V..E..A...............SM..A                                      4057
LR597557.1      17881322  ...F....SS.SGPV..Y...V..E..A...............SM..A                                      17881179
XM_030736458.1  4069      ...F....SS.SGPV..Y...V..E..A...............SM..                                       4209
XM_040116404.1  4076      ...F....SS.SGPV..Y...V..E..A...............SM..                                       4216
XM_028454605.1  5111      ...F....SC.SGPV..Y...V..E..A...............SM..T                                      5254
LR131991.1      13181193  ...F....SC.SGPV..Y...V..E..A...............SM..T                                      13181336
XM_042392722.1  5644      ...F....SS.SGPV..Y...V..E..A...............SMM.T                                      5787
XM_037089353.1  5511      ...F....SG.SGPV..Y...V..E..A...............SM..                                       5651
XM_037089351.1  5624      ...F....SG.SGPV..Y...V..E..A...............SM..                                       5764
LR884482.1      13441505  ...F....SG.SGPV..Y...V..E..A...............SM..                                       13441365
```

```
OU343208.1      15496488  ...F....SS.SGPV..Y...V..E..A...............SMM.T                                           15496345
XM_030407142.1  5583      ...F....SG.SGPV..Y...V..E..A...............SM..                                            5723
LR537143.1      17288045  ...F....SG.SGPV..Y...V..E..A...............SM..                                            17287905
XM_030141392.1  5456      ...F....SSESGPV..Y...V..E..A...............SM..ATNM.ASA.A..STN.                            5668
                                                                            \
                                                                            |
                                                                     VNTSSTNT
LR597465.1      29101385  ...F....SSESGPV..Y...V..E..A...............SM..ATNM.ASA.A..STN.                            29101597
                                                                            \
                                                                            |
                                                                     VNTSSTNT
XM_029508820.1  3862      ...F....SS.SGPV..Y...V..E..A..............SSM..T                                           4005
XM_040190745.1  4420      ...F.F..SG.SGPV..Y...V..E..A...............SM..AA                                          4566
LR584049.1      8069114   ...F....SS.SGPV..Y...V..E..A..............SSM..T                                           8068971
XM_035398742.1  4802      .R.F....SA.SGPV..Y...V..E..............E...SM..                                            4942
XM_035398739.1  5893      .R.F....SA.SGPV..Y...V..E..............E...SM..                                            6033
XM_017435947.3  5424      ...F....SS.SGPV..Y..FV..E..A...............SM..TNTM.PSA.T..SNI..T...EIHDRC.                5681
                                                                         \               \
                                                                         |               |
                                                                  TTSMTTSS             GLS
XM_010889443.4  5921      ...F.F..S..SGTV..Y...V..E................LS.IM..                                           6067
                                        \
                                        |
                                        P
XM_028985601.1  4305      ...F.F.QSA.RTAV.LY...V..E..T..............S.M..V.                                          4451
XM_028985600.1  4326      ...F.F.QSA.RTAV.LY...V..E..T..............S.M..V.                                          4472
XM_028985599.1  4338      ...F.F.QSA.RTAV.LY...V..E..T..............S.M..V.                                          4484
XM_028985598.1  4356      ...F.F.QSA.RTAV.LY...V..E..T..............S.M..V.                                          4502
XM_028985597.1  4368      ...F.F.QSA.RTAV.LY...V..E..T..............S.M..V.                                          4514
LR535819.1      5800280   ...F.F.QSA.RTAV.LY...V..E..T..............S.M..V.                                          5800426
XM_036552240.1  3994      .R.F....SA.SGPV..Y...V..E..A................M.                                             4131
XM_023810583.1  5785      ...F.F..SS.SGPV..Y...V..E..A..............GSV..                                            5925
XM_023810585.1  5866      ...F.F..SS.SGPV..Y...V..E..A..............GSV..                                            6006
XM_038988672.1  3871      ...F.F..SA.SGAV..YL..V..E................LS.M....PSITSP..S.GV..----GL.R.E.                4095
                                        \                                 \
                                        |                                 |
                                        P                              SMAG
XM_029773488.1  4693      ...F.F..SA.SGAV..YL..V..E................LS.M...                                           4839
                                        \
                                        |
                                        P
XM_029773461.1  4937      ...F.F..SA.SGAV..YL..V..E................LS.M...                                           5083
                                        \
                                        |
                                        P
XM_029773480.1  4941      ...F.F..SA.SGAV..YL..V..E................LS.M...                                           5087
                                        \
                                        |
                                        P
XM_029773470.1  5920      ...F.F..SA.SGAV..YL..V..E................LS.M...                                           6066
                                        \
                                        |
                                        P
LR778287.1      42017115  ...F.F..SA.SGAV..YL..V..E................LS.M...SMAGVPSTTSPSL.PRVSTGL.R.E.GMV.             42016879
```

```
                                          \
                                          |
                                          P
XM_030342649.1  5173        ...F....SSESGPV..Y...V..E..A.................SM.TA          5316
LR584410.1      69754430    ...F.F..SA.SGAV..YL..V..E................LS.M...            69754284
                                          \
                                          |
                                          P
LR633944.1      3598072     ...F....SSESGPV..Y...V..E..A.................SM.TA          3597929
XM_031813733.1  5850        ...F.F..SA.SGAV..YL..V..E................LS.M...            5996
                                          \
                                          |
                                          P
XM_041864514.1  5837        ...F.F..SA.SGAV..YL..V..E................LS.M...SMAGVPSTTSPSL.PRVSTGL.R.E.          6061
                                          \
                                          |
                                          P
XM_029646803.1  1299        ...F.F..SA.SGAV..YL..V..E................LS.M...            1445
                                          \
                                          |
                                          P
XM_014202719.1  4930        ...F.F..SA.SGTV..YL..V..E................LS.M...            5076
                                          \
                                          |
                                          P
XM_014202717.1  5161        ...F.F..SA.SGTV..YL..V..E................LS.M...            5307
                                          \
                                          |
                                          P
XM_014202716.1  5174        ...F.F..SA.SGTV..YL..V..E................LS.M...            5320
                                          \
                                          |
                                          P
XM_014202718.1  5178        ...F.F..SA.SGTV..YL..V..E................LS.M...            5324
                                          \
                                          |
                                          P
XM_042318314.1  2430        ...F.F..SA.SGAV..YL..V..E................LS.M...            2576
                                          \
                                          |
                                          P
XM_042318313.1  2453        ...F.F..SA.SGAV..YL..V..E................LS.M...            2599
                                          \
                                          |
                                          P
XM_029728901.1  4904        ...F.F..SE.SGTV..YL..V..E................LS.M...            5050
                                          \
                                          |
                                          P
XM_029728900.1  4934        ...F.F..SE.SGTV..YL..V..E................LS.M...            5080
                                          \
                                          |
                                          P
XM_029728902.1  5928        ...F.F..SE.SGTV..YL..V..E................LS.M...            6074
```

```
                                        \
                                        |
                                        P
LR584408.1      34057537  ...F.F..SE.SGTV..YL..V..E................LS.M...      34057391
                                        \
                                        |
                                        P
XM_036590340.1  3826      ...F....SG.SGPV.EY...V..E..A.L..............T.SA      3969
XM_014194320.1  1933      ...F.F..SA.SGTV..YL..V..E................LS.M...SMAGVP.TTSPSL.SGVSTGL.R.E.      2157
                                        \
                                        |
                                        P
XM_036941748.1  5825      ...F.F..SA.SGAV..YL..V..E................LS.M...      5971
                                        \
                                        |
                                        P
XM_036941749.1  5917      ...F.F..SA.SGAV..YL..V..E................LS.M...      6063
                                        \
                                        |
                                        P
XM_036939096.1  5922      ...F.F..SA.SGAV..YL..V..E................LS.M...      6068
                                        \
                                        |
                                        P
XM_036939094.1  5801      ...F.F..SA.SGAV..YL..V..E................LS.M...      5947
                                        \
                                        |
                                        P
XM_036939095.1  5920      ...F.F..SA.SGAV..YL..V..E................LS.M...      6066
                                        \
                                        |
                                        P
XM_036939093.1  5917      ...F.F..SA.SGAV..YL..V..E................LS.M...      6063
                                        \
                                        |
                                        P
XM_037257125.1  4430      ...F.....DGGGPV..Y...V..E..A..............      4558
                                       \
                                       |
                                       G
XM_037257124.1  4510      ...F.....DGGGPV..Y...V..E..A..............      4638
                                       \
                                       |
                                       G
LR594593.1      702779    ...F.....DGGGPV..Y...V..E..A..............      702651
                                       \
                                       |
                                       G
XM_030782860.1  3900      .R......MNESGHM.K...AV.S.L.PH.........IE.L..KE...      4046
LR697113.1      12194559  .R......MNESGHM.K...AV.S.L.PH.........IE.L..KE...      12194705
                                           HQLSISPDIRVQVDGSIGRSPTDEIANLVP
```

```
                                                N
human            MASPPHQQLLHHHSTEVSCDSSGDSNSVRVKINPK------------------QLSSNSH     42
spotted_gar      MASPRHQPLLLR-HTEVSCDAGGGGVAVKVNSSGGGKPPPPHPPPQHRGPASGSAGGGGG     59
bowfin           MASPQRQKLLLH-HTEVICDSGGDGVSVKINSK-----------PKMHGPASSSSSAGGG     48
                 **** :* ** :  *** **:.*.. :*::: .                    . ....

human            PKHCKYSISSSCSSSGDSGGVPRRVGGGGRLRRQKKLPQLFERASSRWWDPKFDSVNLEE     102
spotted_gar      SKHIKYSISSSCSSGE-SG-VRR--AMVPGLRGQRKVPQLFERSAAQWWDPGFDSPILED     115
bowfin           SKHCKYSISSSCSSGE-SG-VRR--TAVSGLRNQRKVPQLFERSAAQWWDPGFDSPILEE     104
                  ** **********.  ** * *       ** *:*:******::::**** ***  **:

                                         TM1                   TM2
human            ACLERCFPQTQRRFRYALFYIGFACLLWSIYFAVHM-RSRLIVMVAPALCFLLVCVGFFL     161
spotted_gar      ACRERCFPQTQRRFRYVLSYLAAAGLLWGVYFGVNHDRCDPFAFLLPTAGFLAFCLALFL     175
bowfin           ACRERCFPQAQRRFRYVLFYLLAAGLLWGVYFGVNHGRCAPLAFLVPTAGFLVFCSLLFL     164
                 ** ******:******.* *:  * ***.:**.*:  *.  :.:: *:  ** .*  :**

                               TM3
human            FTFTKLYARHYAWTSLALTLLVFALTLAAQFQVLTPV--------SGRGDSSNLTATARP     213
spotted_gar      FTFTRLYARLYPQVSLLLIVVTFAVTLAPQVQTAAFSQSQGREEDPSGNRSNSSGGPPRP     235
bowfin           FTFTRFYARLHTQVSLLLILVTFALTLAPQIQTAAFSDLGIEDVDNGLGNSSIASSGSGS     224
                 ****::*** :  .** * ::.**:*** *.*. :           . . *.   .

                              TM4                     TM5
human            TDTCLSQVGSFSMCIEVLFLLYTVMHLPLYLSLCLGVAYSVLFETFGYHFRDEACFPSPG     273
spotted_gar      GKLCISPVGTFSLCMEVLLLLYSVLHLRLYASVLLGLGYSVLFEALGCVYIAED------     289
bowfin           LTPCISPVGTFSLCMEVLLLLYSVLHLRLYASVLLGLVYSILFETLGCVALAHDG---QA     281
                    *:* **:**:*:***:***:*:** ** *: **: **:***::*     .

                                TM6                             HD1.1
human            AGALHWELLSRGLLHGCIHAIGVHLFVMSQVRSRSTFLKVGQSIMHGKDLEVEKALKERM     333
spotted_gar      HPTLYWLAPGKGLLHLCAHAIGIHLFIMSEVRSRSTFLKVGQAIMHGKDLEVEKALKERM     349
bowfin           SDTLYWLAPGKALLHLCAHAIGIHLFIMSEVRSRSTFLKVGQAIMHGKDLEVEKALKERM     341
                   :*:*   .:.*** * ****:***:**:************:*****************
                              HD1.2                            /C1a catalytic
human            IHSVMPRIIADDLMKQGDEESENSVKRHATS-SPKNRKKKSSIQKAPIAFRPFKMQQIEE     392
spotted_gar      IHSVMPRRVADELMKQGDDESETSVKRYSTS-SPKSKKKKTSIPRGQIIFRPFNMKRMEP     408
bowfin           IHSVMPRRVADELMKQGDDESENSVKRYSTSSSPKNKKKKTSIPRGQIIFRPFNMKRMEP     401
                 ******* :**:******:***.****::** ***.:***:** :. * ****:*:::*

                                     C1a catalytic
human            VSILFADIVGFTKMSANKSAHALVGLLNDLFGRFDRLCEETKCEKISTLGDCYYCVAGCP     452
spotted_gar      VSILFADIVGFTKMSANKSAHALVGLLNDLFGRFDRLCEVTKCEKISTLGDCYYCVAGCP     468
bowfin           VSILFADIVGFTKMSANKSAHALVGLLNDLFGRFDRLCEITECEKISTLGDCYYCVAGCP     461
                 *************************************** *:******************

                                     C1a catalytic
human            EPRADHAYCCIEMGLGMIKAIEQFCQEKKEMVNMRVGVHTGTVLCGILGMRRFKFDVWSN     512
spotted_gar      EPRADHAYCCIEMGLGMIEAIEQFCQEKKEMVNMRVGVHTGTVLCGILGMKRFKFDVWSN     528
bowfin           EPRDDHAYCCIEMGLGMIAAIEQFCQEKKEMVNMRVGVHTGTVLCGILGMKRFKFDVWSN     521
                 *** ************** ******************************:*********
```

```
                                     C1a catalytic                    /C1b
human           DVNLANLMEQLGVAGKVHISEATAKYLDDRYEMEDGKVIERLGQSVVADQLKGLKTYLIS     572
spotted_gar     DVNLANLMEQLGVAGKVHISEVTAGFLDDRYEREDGRVLERVGQNVVADQLKGLKTYLIS     588
bowfin          DVNLANLMEQLGVAGKVHISEVTANFLDDRYEREDGRVMERVGQSVVADQLKGLKTYLIS     581
                *********************.** :****** ***:*:**:**.***************
                                         C1b
human           GQRAKESRCSCAEALLSGFEVIDGSQVSSGPRGQGTA-SSGNVSDLAQTVKTFDNLKTCP     631
spotted_gar     GQRVEHSQCSCSQLGLTGPDPGDGPAAAPRAQSPDATPAAGPDSGLA-HCQADRAKVPCP     647
bowfin          GRRAEPSQCSCSQLGPAGQDPGDGLPTASRAQTPDPAPTPG--GTLP-CGPADRAKPPCP     638
                *:*.: *:***::   :* :  **  .:   :  . : : *  . *     :      **


                                         C1b
human           SCGITFAPKSEAGAEGGAPQNGCQDEHKNSTKASGGPNPKTQNGLLSPPQEEKLTNSQTS     691
spotted_gar     SCSVVLIPSSELAVEDGTVQNGCQEEHKNNAKVPSGRGPKAQNGLLSPPADDKMTNSQTS     707
bowfin          SCSVDPMPSCEVPVDDGAVQNGCQEEHKNNAKVLPGRSAKAQNGLLSPPPEDKLTNSQTS     698
                **.:   *..*  .:.*: *****:****.:*.  * . *:******** ::*:******

                                         C1b
human           LCEILQEK-GRWAGVSLDQSALLPLRFKNIREKTDAHFVDVIKEDSLMKDYFFKPPINQF     750
spotted_gar     LCEMLQEKEKKWGGVSMDHSALIPLRSKNFRERSDAHFVDVIKEDSLMKDYFFKPPINKL     767
bowfin          LCEMLQEKEKKWGVVSMDHSALIPLRSKNFRERSDAHFVDVIKEDSLMKDYFFKPPINKL     758
                ***:****  :*. **:*:***:*** **:**::************************::

                                                  TM7
human           SLNFLDQELERSYRTSYQEEVIKNSPVKTFASPTFSSLLDVFLSTTVFLTLSTTCFLKYE     810
spotted_gar     SLNFLEKPLESAYRASYQEEVQTKAPVQTFASPTFSSFLDVLLSSAVFAALAVACFLRPW     827
bowfin          SLNFLEKTLESAYRASYKEEVKTRAPVQTFASPTFSSFLDILLSSAVFLALTVACFLRPC     818
                *****:: ** :**:**:*** ..:**:*********:**::**::** :*:.:***:

                                TM8
human           AATVPPPPAALAVFSAALLLEVLSLAVSIRMVFFLEDVMACTKRLLEWIAGWLPRHCIGA     870
spotted_gar     VTGSPPPAAAIAVCVLAVLLEGISLVLSVRMAFYLDNVMSCTRQLLQVISGWIPRHLIGA     887
bowfin          ISGPPPPPAAIAVCVLAVLLEGVSLGLSVRMAFYLDSVMSCTRRLLLAISGSVPRHLIGA     878
                 :  *** **:**   *:*** :** :*:**.*:*:.**:**::**  *:* :*** ***

                           TM9                     TM10                  TM11
human           ILVSLPALAVYSHVTSEYETNIHFPVFTGSAALIAVVHYCNFCQLSSWMRSSLATVVGAG     930
spotted_gar     VLISLPALSVFTHITCQFHLSLQFTMFICCAVIITIIQYCNFCQLSFWMRSSFATAVGAV     947
bowfin          GLISLPALSVFTHITCPFHLSIQFTMFLCCAATIAIIHYCNFCQLSFWMRSALATAVGAL     938
                 *:*****:*::*:*. :. .::* :*  .*. *::::******** ****::**.***
```

```
                    TM11                                              TM12
human            PLLLLYVSLCPDSSVLTSPLDAVQNFSSERNPCNSSV-----PRDLRRPASLIGQEVVLV     985
spotted_gar      LLAVLYTPLCARGGSPSDSL---------------------------SAPAVALQDVSLA     980
bowfin           LLVLLYAPLCAHHSSLTPATETFSGFDSTNGSTAEGTETGPDAGAGTRPQDLIGPEAILA     998
                  * :**. **   .  :                                  :   :.  *.

                    TM12
human            FFLLLLLVWFLNREFEVSYRLHYHGDVEADLHRTKIQSMRDQADWLLRNIIPYHVAEQLK     1045
spotted_gar      FFLLLLLVWFLNREFEVSYRLHYHGNVEADQHRIKIQNMRDQADWLLRNIIPIHVAEQLK     1040
bowfin           FFLLLLLVWFLNREFEVSYRLHYHGNVEADQHRIKIQNMRDQADWLLRNIIPVHVSEQLK     1058
                 *************************:**** ** ***.************** **:****

                     /C2a catalytic
human            VSQTYSKNHDSGGVIFASIVNFSEFYEENYEGGKECYRVLNELIGDFDELLSKPDYSSIE     1105
spotted_gar      VTQSYSKNHDNVGVIFASIVNFSEFYEENYEGGKECYRVLNELIGDFDELLRKPAFGNIE     1100
bowfin           VTQSYSKNHDNVGVIFASIVNFSEFYEENYEGGKECYRVLNELIGDFDELLRKPAFNNIE     1118
                 *:*:******. *************************************** ** :..**

human            KIKTIGATYMAASGLNTAQAQDGSHPQEHLQILFEFAKEMMRVVDDFNNNMLWFNFKLRV     1165
spotted_gar      KIKTIGATYMAASGLNTSQCQDSAHPHGHLRTLFDFAMEMMRVVDDFNKDMLWFNFKLRI     1160
bowfin           KIKTIGATYMAASGLNASQCQDSAHPHGHLRTLFDFAMEMMRVVDDFNKDMLWFNFKLRI     1178
                 ****************::*.**.:**: **: **:** **********::*********:

human            GFNHGPLTAGVIGTTKLLYDIWGDTVNIASRMDTTGVECRIQVSEESYRVLSKMGYDFDY     1225
spotted_gar      GFNHGPLTAGVIGTTKLLYDIWGDTVNIASRMDTTGVECRVQVSEESYRVLKEMGYEFDY     1220
bowfin           GFNHGPLTAGVIGTTKLLYDIWGDTVNIASRMDTTGVECRVQVSEESHRVLKEMGYEFDY     1238
                 ****************************************:******:***.:***:***
                                 /C2b                     AIM
human            RGTVNVKGKGQMKTYLYPKCTDHRVIPQHQLSISPDIRVQVDGSIGRSPTDEIANLVPSV     1285
spotted_gar      RGTVNVKGKGQMKTFLFPKSVDSGMVPQHQLSVSPDIRVQVDGSIGRSPTDEIAGVVPSA     1280
bowfin           RGTVNVKGKGQMKTFLFPKSVDSGMVPQHQLSVSPDIRVQVDGSIGRSPTDELANMVPTC     1298
                 **************:*:**..*  ::******:*******************:*.:**:

human            QYVDKTS--LGSDSSTQAKDAHLSPKR---------PWKEP-------------------     1315
spotted_gar      SPQGQERGLQGAGEKPDSQNSSGGPPRSSAPAGPGSPGEDQRPPPARAASVPSAPAPRPG     1340
bowfin           ATQGPERGPQRATETPEAKDMLGQPKGPAAVA--TSPVQGPRPAPAHATASTPAPAAPTG     1356
                    .       : .. ::::    *           * :

human            -----VKAEERGRFGKAIEKDDCDETGIEEANELTKLNVSKSV-----------------     1353
spotted_gar      PPEEEEPEVNELTK----------------------LKVAE---SL--------------     1361
bowfin           PLKQEGQEEERGGAGGGAERTDETQTCR---QPMMALAVGRQCEKLQSYPVDASCPSGPL     1413
                          :.                         * *..

human            --------------------------------1353
spotted_gar      --------------------------------1361
bowfin           PLHSIFLTVLFWDFLCFFWLIFSQGGALCGSQ1445
```

```
chr22x3sterlet       MASPQHQQLLLHSTEVSCNATGDSVSVKINSKPQLHDPASSSSSSSAGGGG--GSSSKHC   58
chr22x2sterlet       MASPQHQQLLLHSTEVSCNATGDSVSVKINSKPQLHDPASSSSSSSAGGGG--GSSSKHC   58
chr22x1sterlet       MASPQHQQLLLHSTEVSCNATGDSVSVKINSKPQLHDPASSSSSSSAGGGG--GSSSKHC   58
chr26x2paddlefish    MASPQHQQLLLHSTEVSCNTTGDSVSVKINSKPQLHDPASSTSSSSAGGGGGGSSSSKHC   60
chr26x1paddlefish    MASPQHQQLLLHSTEVSCNTTGDSVSVKINSKPQLHDPASSTSSSSAGGGGGGSSSSKHC   60
chr13x2sterlet       MASPQHQQLLLHSTEVSCNAAGDSVSVKINSKPQLHGPASSSSSS--------NSSSKHC   52
chr13x1sterlet       MASPQHQQLLLHSTEVSCNAAGDSVSVKINSKPQLHGPASSSSSS--------NSSSKHC   52
chr18x1paddlefish    MASPQHQQLLLHSTEVSCSATGDSVSVKINSKPQLHGPASSSSSS--------NSSSKHC   52
chr18x2paddlefish    MASPQHQQLLLHSTEVSCSATGDSVSVKINSKPQLHGPASSSSSS--------NSSSKHC   52
                     ******************.::***************.****:***        .******

chr22x3sterlet       KYSISSSCSSGESGVRRVATAGARRPKKLPQLFERSSWQWWNPRFDSNNLEEACLERCFP   118
chr22x2sterlet       KYSISSSCSSGESGVRRVATAGARRPKKLPQLFERSSWQWWNPRFDSNNLEEACLERCFP   118
chr22x1sterlet       KYSISSSCSSGESGVRRVATAGARRPKKLPQLFERSSWQWWNPRFDSNNLEEACLERCFP   118
chr26x2paddlefish    KYSISSSSSSGESGVRRVATAGARRPKKLPQLFERSSWQWWNPRFDSTNLEEACLERCFP   120
chr26x1paddlefish    KYSISSSSSSGESGVRRVATAGARRPKKLPQLFERSSWQWWNPRFDSTNLEEACLERCFP   120
chr13x2sterlet       KYSISSSCSSGESGVRRVVTTGARRPKKLPQLFERSSWQWWNPRFDSTNLEEACLERCFP   112
chr13x1sterlet       KYSISSSCSSGESGVRRVVTTGARRPKKLPQLFERSSWQWWNPRFDSTNLEEACLERCFP   112
chr18x1paddlefish    KYSISSSCSSGESGVRRVVTTRARRPKKLPQLFERSSWQWWNPKFDSTNLEEACLERCFP   112
chr18x2paddlefish    KYSISSSCSSGESGVRRVVTTRARRPKKLPQLFERSSWQWWNPKFDSTNLEEACLERCFP   112
                     *******.**********.*: *********************:***.************

chr22x3sterlet       QTQRRFRYVLFYLAAACLLWSIYFGVNGARCDWTAFLVPTLGFLVFCMLLLLFTFTRFYA   178
chr22x2sterlet       QTQRRFRYVLFYLAAACLLWSIYFGVNGARCDWTAFLVPTLGFLVFCMLLLLFTFTRFYA   178
chr22x1sterlet       QTQRRFRYVLFYLAAACLLWSIYFGVNGARCDWTAFLVPTLGFLVFCMLLLLFTFTRFYA   178
chr26x2paddlefish    QTQRRFRYVLFYLAVACLLWSIYFGVNGARCDWTAFLVPTLGFLIFCMLLLLFTFTRFYA   180
chr26x1paddlefish    QTQRRFRYVLFYLAVACLLWSIYFGVNGARCDWTAFLVPTLGFLIFCMLLLLFTFTRFYA   180
chr13x2sterlet       QTQRRFRYVLFYLAAACLLWSIYFGVNGARCDQTAFLIPTLGFLVFCILLLLFTFTRFYA   172
chr13x1sterlet       QTQRRFRYVLFYLAAACLLWSIYFGVNGARCDQTAFLIPTLGFLVFCILLLLFTFTRFYA   172
chr18x1paddlefish    QTQRRFRYVLFYLVAACLLWSIYFRVNGARCDQTAFLVPTLGFLVFCILLLLFTFTRFYA   172
chr18x2paddlefish    QTQRRFRYVLFYLVAACLLWSIYFRVNGARCDQTAFLVPTLGFLVFCILLLLFTFTRFYA   172
                     *************..********* ******* ****:******:**:************

chr22x3sterlet       RLYVWVSLLLFIVTFALTLSPQLQRAFFLKEDDFDNSTGLTNAAYSWEPCLSPVGTFSLC   238
chr22x2sterlet       RLYVWVSLLLFIVTFALTLSPQLQRAFFLKEDDFDNSTGLTNAAYSWEPCLSPVGTFSLC   238
chr22x1sterlet       RLYVWVSLLLFIVTFALTLSPQLQRAFFLKEDDFDNSTGLTNAAYSWEPCLSPVGTFSLC   238
chr26x2paddlefish    RLYVWVSLLLFIVTFALTLSLQLQRAFFLKEGDFDNSTGLTNAAYSWEPCLSPVGTFSLC   240
chr26x1paddlefish    RLYVWVSLLLFIVTFALTLSLQLQRAFFLKEGDFDNSTGLTNAAYSWEPCLSPVGTFSLC   240
chr13x2sterlet       RLYVWVSLLLFLVTFALTLSPQLQRAFFLKEGDFDNSTGLTNAAYSWEPCLSNVGTFSVC   232
chr13x1sterlet       RLYVWVSLLLFLVTFALTLSPQLQRAFFLKEGDFDNSTGLTNAAYSWEPCLSNVGTFSVC   232
chr18x1paddlefish    RLYVWVSLLLFIVTFALTLSPQLQHAFFLKDGDFDNSTGLTNAAYTWEPCLSPVGTFSLC   232
chr18x2paddlefish    RLYVWVSLLLFIVTFALTLSPQLQHAFFLKDGDFDNSTGLTNAAYTWEPCLSPVGTFSLC   232
                     ***********:******** ***:*****:.*************:****** *****:*

chr22x3sterlet       MEVLLLLYTIMHLHLYLCVLLGLVYSALFEVLGYIYFANDSAESNPLYWLAPAKALLHIC   298
chr22x2sterlet       MEVLLLLYTIMHLHLYLCVLLGLVYSALFEVLGYIYFANDSAESNPLYWLAPAKALLHIC   298
chr22x1sterlet       MEVLLLLYTIMHLHLYLCVLLGLVYSALFEVLGYIYFANDSAESNPLYWLAPAKALLHIC   298
chr26x2paddlefish    MEVLLLLYTIMHLHLYLCVLLGLVYSMLFEILGCIYFANDSTESNPLYWLAPAKALLHIC   300
chr26x1paddlefish    MEVLLLLYTIMHLHLYLCVLLGLVYSMLFEILGCIYFANDSTESNPLYWLAPAKALLHIC   300
chr13x2sterlet       MEVLLLLYTIMHLHLYLCVLLGLVYSTLFEILGCIYFASDSAESNPLYWLAPAKALLHIC   292
chr13x1sterlet       MEVLLLLYTIMHLHLYLCVLLGLVYSTLFEILGCIYFASDSAESNPLYWLAPAKALLHIC   292
chr18x1paddlefish    MEVLLLLYTIMHLHLYLCVLLGLVYSTLFEILGWFYFASDSTESNPLYWLAPAKALLHIC   292
chr18x2paddlefish    MEVLLLLYTIMHLHLYLCVLLGLVYSTLFEILGWFYFASDSTESNPLYWLAPAKALLHIC   292
                     ************************** ***:** :***.**:******************

chr22x3sterlet       AHAIGIHLFIMSEVRSRSTFLKVGQSIMHGKDLEVEKALKERMIHSVMPRMVADELMKQG   358
chr22x2sterlet       AHAIGIHLFIMSEVRSRSTFLKVGQSIMHGKDLEVEKALKERMIHSVMPRMVADELMKQG   358
chr22x1sterlet       AHAIGIHLFIMSEVRSRSTFLKVGQSIMHGKDLEVEKALKERMIHSVMPRMVADELMKQG   358
chr26x2paddlefish    AHAIGIHLFIMSEVRSRSTFLKVGQSIMHGKDLEVEKALKERMIHSVMPRMVADELMKQG   360
chr26x1paddlefish    AHAIGIHLFIMSEVRSRSTFLKVGQSIMHGKDLEVEKALKERMIHSVMPRMVADELMKQG   360
chr13x2sterlet       AHATGIHLFIMSEVRSRSTFLKVGQSIMHGKDLEVEKALKERMIHSVMPRMVADELMKQG   352
chr13x1sterlet       AHATGIHLFIMSEVRSRSTFLKVGQSIMHGKDLEVEKALKERMIHSVMPRMVADELMKQG   352
chr18x1paddlefish    AHAIGIHLFIMSEVRSRSTFLKVGQSIMHGKDLEVEKALKERMIHSVMPRMVADELMKQG   352
chr18x2paddlefish    AHAIGIHLFIMSEVRSRSTFLKVGQSIMHGKDLEVEKALKERMIHSVMPRMVADELMKQG   352
                     *** ********************************************************
```

```
chr22x3sterlet        DEESENSVKRYSTSSPKNKKKKPSIPKAQIIFRPFNMKRMEPVSILFADIVGFTKMSANK   418
chr22x2sterlet        DEESENSVKRYSTSSPKNKKKKPSIPKAQIIFRPFNMKRMEPVSILFADIVGFTKMSANK   418
chr22x1sterlet        DEESENSVKRYSTSSPKNKKKKPSIPKAQIIFRPFNMKRMEPVSILFADIVGFTKMSANK   418
chr26x2paddlefish     DEESENSVKRYSTSSPKNKKKKPSIPKAQIIFRPFNMKRMEPVSILFADIVGFTKMSANK   420
chr26x1paddlefish     DEESENSVKRYSTSSPKNKKKKPSIPKAQIIFRPFNMKRMEPVSILFADIVGFTKMSANK   420
chr13x2sterlet        DDESENSVKRYSTSSPKNKKKKPSIPKAQIIFRPFNMKRMEPVSILFADIVGFTKMSANK   412
chr13x1sterlet        DDESENSVKRYSTSSPKNKKKKPSIPKAQIIFRPFNMKRMEPVSILFADIVGFTKMSANK   412
chr18x1paddlefish     DDESENSVKRYSTSSPKNKKKKPSIPKAQIIFRPFNMKRMEPVSILFADIVGFTKMSANK   412
chr18x2paddlefish     DDESENSVKRYSTSSPKNKKKKPSIPKAQIIFRPFNMKRMEPVSILFADIVGFTKMSANK   412
                      *:**********************************************************

chr22x3sterlet        SAHALVSLLNDLFGRFDRLCEITNCEKISTLGDCYYCVAGCPEPRADHAYCCIEMGLGMI   478
chr22x2sterlet        SAHALVSLLNDLFGRFDRLCEITNCEKISTLGDCYYCVAGCPEPRADHAYCCIEMGLGMI   478
chr22x1sterlet        SAHALVSLLNDLFGRFDRLCEITNCEKISTLGDCYYCVAGCPEPRADHAYCCIEMGLGMI   478
chr26x2paddlefish     SAHALVSLLNDLFGRFDRLCEITNCEKISTLGDCYYCVAGCPEPRADHAYCCIEMGLGMI   480
chr26x1paddlefish     SAHALVSLLNDLFGRFDRLCEITNCEKISTLGDCYYCVAGCPEPRADHAYCCIEMGLGMI   480
chr13x2sterlet        SAHALVGLLNDLFGRFDRLCEITNCEKISTLGDCYYCVAGCPEPRADHAYCCIEMGLGMI   472
chr13x1sterlet        SAHALVGLLNDLFGRFDRLCEITNCEKISTLGDCYYCVAGCPEPRADHAYCCIEMGLGMI   472
chr18x1paddlefish     SAHALVGLLNDLFGRFDRLCEITNCEKISTLGDCYYCVAGCPEPRADHAYCCIEMGLGMI   472
chr18x2paddlefish     SAHALVGLLNDLFGRFDRLCEITNCEKISTLGDCYYCVAGCPEPRADHAYCCIEMGLGMI   472
                      ******.*****************************************************

                                                             4alpha helix
chr22x3sterlet        EAIEQFCQEKKEMVNMRVGVHTGTVLCGILGMRRFKFDVWSNDVNLANLMEQLGVAGKVH   538
chr22x2sterlet        EAIEQFCQEKKEMVNMRVGVHTGTVLCGILGMRRFKFDVWSNDVNLANLMEQLGVAGKVH   538
chr22x1sterlet        EAIEQFCQEKKEMVNMRVGVHTGTVLCGILGMRRFKFDVWSNDVNLANLMEQLGVAGKVH   538
chr26x2paddlefish     EAIEQFCQEKKEMVNMRVGVHTGTVLCGILGMRRFKFDVWSNDVNLANLMEQLGVAGKVH   540
chr26x1paddlefish     EAIEQFCQEKKEMVNMRVGVHTGTVLCGILGMRRFKFDVWSNDVNLANLMEQLGVAGKVH   540
chr13x2sterlet        KAIEQFCQEKKEMVNMRVGVHTGTVLCGILGMRRFKFDVWSNDVNLANLMEQLGVAGKVH   532
chr13x1sterlet        KAIEQFCQEKKEMVNMRVGVHTGTVLCGILGMRRFKFDVWSNDVNLANLMEQLGVAGKVH   532
chr18x1paddlefish     RAIEQFCQEKKEMVNMRVGVHTGTVLCGILGMRRFKFDVWSNDVNLANLMEQLGVAGKVH   532
chr18x2paddlefish     RAIEQFCQEKKEMVNMRVGVHTGTVLCGILGMRRFKFDVWSNDVNLANLMEQLGVAGKVH   532
                      .***********************************************************

chr22x3sterlet        ISEVTASFLDDRYLREDGRVMERVGQSVVADQLKGL------------------------   574
chr22x2sterlet        ISEVTASFLDDRYLREDGRVMERVGQSVVADQLKGLLQSLSLVAASAGRNGTGAAADYPQ   598
chr22x1sterlet        ISEVTASFLDDRYLREDGRVMERVGQSVVADQLKGLLQSLSLVAASAGRNGTGAAADYPQ   598
chr26x2paddlefish     ISEVTASFLDDRYLREDGRVMERVGQSVVADQLKGL------------------------   576
chr26x1paddlefish     ISEVTASFLDDRYLREDGRVMERVGQSVVADQLKGLLQSLSLVAASAGRNGTGAAADYPQ   600
chr13x2sterlet        ISEVTASFLDDRYLREDGRVMERVGQSVVADQLKGL------------------------   568
chr13x1sterlet        ISEVTASFLDDRYLREDGRVMERVGQSVVADQLKGLLQSLSLVAASAGRNGTGAAADSSQ   592
chr18x1paddlefish     ISEVTASFLDDRYLREDGRVMERVGQSVVADQLKGLLQSLSLVAASAGRNGTGAAADSSQ   592
chr18x2paddlefish     ISEVTASFLDDRYLREDGRVMERVGQSVVADQLKGL------------------------   568
                      ************************************

chr22x3sterlet        ------------------KTYLISGRKVERSPCSCSQLGLAGSESGDGASRAQTPETITT   616
chr22x2sterlet        QSFVDADSTYWGACRWRLKTYLISGRKVERSPCSCSQLGLAGSESGDGASRAQTPETITT   658
chr22x1sterlet        QSFVDADSTYWGACRWRLKTYLISGRKVERSPCSCSQLGLAGSESGDGASRAQTPETITT   658
chr26x2paddlefish     ------------------KTYLISGRKVERSPCSCSQLGLARSESGDGAFRVQTPETITT   618
chr26x1paddlefish     QSFVDADSTYWGACRWRLKTYLISGRKVERSPCSCSQLGLARSESGDGAFRVQTPETITT   660
chr13x2sterlet        ------------------KTYLISGRKVERSQCSCSQLGLAGSESGDGASRPQTPETITT   610
chr13x1sterlet        QSCVDADSTYWGACRWRLKTYLISGRKVERSQCSCSQLGLAGSESGDGASRPQTPETITT   652
chr18x1paddlefish     QSCVDADSTYWGACRWRLKTYLISGRKVERSQCRCSQLGLAGSEYVDGASRTQTSETITT   652
chr18x2paddlefish     ------------------KTYLISGRKVERSQCRCSQLGLAGSEYVDGASRTQTSETITT   610
                                        ************* * ******* **  *** * ** *****

chr22x3sterlet        TEATHTNSQTDRTKEPCPSCSVMLVPSCDTSIEDSTVQNGCQEDHKNNTKPIPAGLSLKN   676
chr22x2sterlet        TEATHTNSQTDRTKEPCPSCSVMLVPSCDTSIEDSTVQNGCQEDHKNNTKPIPAGLSLKN   718
chr22x1sterlet        TEATHTNSQTDRTKEPCPSCSVMLVPSCDTSIEDSTVQNGCQEDHKNNTKPIPAGLSLKN   718
chr26x2paddlefish     TEATHSNSQNDKTKEPCPSCSVMLVPSCDTPVEDSTVQNGCQEDHKNNTKSIPGGLSLNT   678
chr26x1paddlefish     TEATHSNSQNDKTKEPCPSCSVMLVPSCDTPVEDSTVQNGCQEDHKNNTKSIPGGLSLNT   720
chr13x2sterlet        TEATHTSSQTDRTKEPCPLCSVMLVPSCDTPVEDSTVQNGCQEDHKNNTKPIPAGHSLKM   670
chr13x1sterlet        TEATHTSSQTDRTKEPCPLCSVMLVPSCDTPVEDSTVQNGCQEDHKNNTKPIPAGHSLKM   712
chr18x1paddlefish     TEATHTSSQTDRTKELCPSCSVMLVPTCDTPTEDSTVQNGCQEDHKNNIKPIPDAR-CKT   711
chr18x2paddlefish     TEATHTSSQTDRTKELCPSCSVMLVPTCDTPTEDSTVQNGCQEDHKNNIKPIPDAR-CKT   669
                      *****:.**.*:*** ** *******:***  **************** * ** .   :
```

```
chr22x3sterlet        QNGLLSPPPEDKLTNSQTSLCEMLQEKEKGKWVGVSMDQSALLPLRFKNIRERTDAHFVD   736
chr22x2sterlet        QNGLLSPPPEDKLTNSQTSLCEMLQEKEKGKWVGVSMDQSALLPLRFKNIRERTDAHFVD   778
chr22x1sterlet        QNGLLSPPPEDKLTNSQTSLCEMLQEKEKGKWVGVSMDQSALLPLRFKNIRERTDAHFVD   778
chr26x2paddlefish     QNGMLSPPPEDKLTNSQTSLCEMLQEKEKGKWVGVSMDQSALLPLRFKNIRERTDGHFVD   738
chr26x1paddlefish     QNGMLSPPPEDKLTNSQTSLCEMLQEKEKGKWVGVSMDQSALLPLRFKNIRERTDGHFVD   780
chr13x2sterlet        QNGLLSLPPEEKLTNSQTSLCEMLQEKEKGKWVGVSMDQSALLPLRFKNIRERTDAHFVD   730
chr13x1sterlet        QNGLLSLPPEEKLTNSQTSLCEMLQEKEKGKWVGVSMDQSALLPLRFKNIRERTDAHFVD   772
chr18x1paddlefish     QNGLLSPPPEEKLTNSQTSLCEMLQEKEKGKWVGVSMDQSALLPLRFKNIRERTDAHFVD   771
chr18x2paddlefish     QNGLLSPPPEEKLTNSQTSLCEMLQEKEKGKWVGVSMDQSALLPLRFKNIRERTDAHFVD   729
                      ***:** ***:********************************************.****

chr22x3sterlet        VIKEDSLMKDYFFKPPINKLSLNFLEKDLEMTYRISYQKEVKDRVPVKTFASSTFSSFID   796
chr22x2sterlet        VIKEDSLMKDYFFKPPINKLSLNFLEKDLEMTYRISYQKEVKDRVPVKTFASSTFSSFID   838
chr22x1sterlet        VIKEDSLMKDYFFKPPINKLSLNFLEKDLEMTYRISYQKEVKDRVPVKTFASSTFSSFID   838
chr26x2paddlefish     VIKEDSLMKDYFFKPPINKLSLNFLEKDLEMTYRISYQKEVKERVPVKTFASSTFSSFID   798
chr26x1paddlefish     VIKEDSLMKDYFFKPPINKLSLNFLEKDLEMTYRISYQKEVKERVPVKTFASSTFSSFID   840
chr13x2sterlet        VIKEDSLMKDYFFKPPINKLSLNFLEKDLEMTYRISYQKEVKDRVPVKTFASSTFSSFID   790
chr13x1sterlet        VIKEDSLMKDYFFKPPINKLSLNFLEKDLEMTYRISYQKEVKDRVPVKTFASSTFSSFID   832
chr18x1paddlefish     VIKEDSLMKDYFFKPPINKLSLNFLEKGLEMTYRISYQKEVKDRVPVKTFASSTFSSFID   831
chr18x2paddlefish     VIKEDSLMKDYFFKPPINKLSLNFLEKGLEMTYRISYQKEVKDRVPVKTFASSTFSSFID   789
                      ***************************.**************:*****************

chr22x3sterlet        VLLSSAVFLFLTIGCFLKPWVSGTTAPPAAIAVCVLAILLELLSLVLSVRMAFYLDEVMG   856
chr22x2sterlet        VLLSSAVFLFLTIGCFLKPWVSGTTAPPAAIAVCVLAILLELLSLVLSVRMAFYLDEVMG   898
chr22x1sterlet        VLLSSAVFLFLTIGCFLKPWVSGTTAPPAAIAVCVLAILLELLSLVLSVRMAFYLDEVMG   898
chr26x2paddlefish     VLLSSAVFLFLTIGCFLKPWVSGTAAPPAAIAVCVLAILLELLSLVLSVRMAFYLDEVMG   858
chr26x1paddlefish     VLLSSAVFLFLTIGCFLKPWVSGTAAPPAAIAVCVLAILLELLSLVLSVRMAFYLDEVMG   900
chr13x2sterlet        VLLSSAVFLFLTIGCFLKPWVSETAAPPAAVAVCVLAILLELVSLVLSVRMAFYLEEVTG   850
chr13x1sterlet        VLLSSAVFLFLTIGCFLKPWVSETAAPPAAVAVCVLAILLELVSLVLSVRMAFYLEEVTG   892
chr18x1paddlefish     VLLSSAVFLFLTIGCFLKPWVSGTAAPPAAVAVCVLAILLELLSLVLSVRMAFYLEEVAG   891
chr18x2paddlefish     VLLSSAVFLFLTIGCFLKPWVSGTAAPPAAVAVCVLAILLELLSLVLSVRMAFYLEEVAG   849
                      ********************** *:*****:***********:************:** *

chr22x3sterlet        CTKHLLQVISGWIPRHFIGAILISIPALSVFSHITCEFDSSIHFTMFMCCAIIITIIQYC   916
chr22x2sterlet        CTKHLLQVISGWIPRHFIGAILISIPALSVFSHITCEFDSSIHFTMFMCCAIIITIIQYC   958
chr22x1sterlet        CTKHLLQVISGWIPRHFIGAILISIPALSVFSHITCEFDSSIHFTMFMCCAIIITIIQYC   958
chr26x2paddlefish     CTKHLLQVISGWIPRHFIGAILISVPALSVFSHITCEFDSSIHFTMFKCCAIIITIIQYC   918
chr26x1paddlefish     CTKHLLQVISGWIPRHFIGAILISVPALSVFSHITCEFDSSIHFTMFKCCAIIITIIQYC   960
chr13x2sterlet        CTKHLLQVISGWIPRHFIGAILISLPALSVFSHITCEFDTSSHFTMFMCCAIIITIIQYC   910
chr13x1sterlet        CTKHLLQVISGWIPRHFIGAILISLPALSVFSHITCEFDTSSHFTMFMCCAIIITIIQYC   952
chr18x1paddlefish     CTKHLLQVISGWIPRHFIGAILISLPALAVFSHITCEFDTSSHFTMFICCAIIITIIQYC   951
chr18x2paddlefish     CTKHLLQVISGWIPRHFIGAILISLPALAVFSHITCEFDTSSHFTMFICCAIIITIIQYC   909
                      ************************:**:***********:* ***** ************

chr22x3sterlet        NFCQLSSWMRSSIATVVGAVLLILLYTSLCPEETATLLSSTLSSRLGSSNSSVPEMLQPL   976
chr22x2sterlet        NFCQLSSWMRSSIATVVGAVLLILLYTSLCPEE------------LGSSNSSVPEMLQPL   1006
chr22x1sterlet        NFCQLSSWMRSSIATVVGAVLLILLYTSLCPEETATLLSSTLSSRLGSSNSSVPEMLQPL   1018
chr26x2paddlefish     NFCQLSSWMRSSIATVVGAVLLILLYTSLCPEEP------------GSSNSSVPEMLQPL   966
chr26x1paddlefish     NFCQLSSWMRSSIATVVGAVLLILLYTSLCPEEP------------GSSNSSVPEMLQPL   1008
chr13x2sterlet        NFCQLSSWMRSSIATVVGAVLLILLYTSLCPEEP------------GFSNSSVPETLQPL   958
chr13x1sterlet        NFCQLSSWMRSSIATVVGAVLLILLYTSLCPEEP------------GFSNSSVPETLQPL   1000
chr18x1paddlefish     NFCQLSSWMRSFISTVVGVVLLILLYTYLYPEEL------------GSSNSSVPETLQLL   999
chr18x2paddlefish     NFCQLSSWMRSFISTVVGVVLLILLYTYLYPEEL------------GSSNSSVPETLQLL   957
                      *********** *:****.******** * ***             * ******* ** *
```

```
chr22x3sterlet            DLNRQEVILAFFLLLLLVWFLNREFEVSYRLHYHGNVEADLHRAKIQTMRDQADWLLRNI   1036
chr22x2sterlet            DLNRQEVILAFFLLLLLVWFLNREFEVSYRLHYHGNVEADLHRAKIQTMRDQADWLLRNI   1066
chr22x1sterlet            DLNRQEVILAFFLLLLLVWFLNREFEVSYRLHYHGNVEADLHRAKIQTMRDQADWLLRNI   1078
chr26x2paddlefish         DLNRQEVILAFFLLLLLVWFLNRELEVSYRLHYHGNVEADLHRAKIQTMRDQADWLLRNI   1026
chr26x1paddlefish         DLNRQEVILAFFLLLLLVWFLNRELEVSYRLHYHGNVEADLHRAKIQTMRDQADWLLRNI   1068
chr13x2sterlet            DLNRQEVILAFFLLLLLVWFLNREFEVSYRLHYHGNVEADLHRAKIQTMRDQADWLLRNI   1018
chr13x1sterlet            DLNRQEVILAFFLLLLLVWFLNREFEVSYRLHYHGNVEADLHRAKIQTMRDQADWLLRNI   1060
chr18x1paddlefish         DLNREEVILAFFLLLLLVWFLNREFEVSYRLHYHGNVEADLHRAKIQTMRDQADWLLRNI   1059
chr18x2paddlefish         DLNREEVILAFFLLLLLVWFLNREFEVSYRLHYHGNVEADLHRAKIQTMRDQADWLLRNI   1017
                          ****:*******************:***********************************

                                                                       Y1082
chr22x3sterlet            IPYHVAEQLKVTQSYSKNHDNVGVIFASIVNFSEFYEENYEGGKECYRVLNELIGDFDEL   1096
chr22x2sterlet            IPYHVAEQLKVTQSYSKNHDNVGVIFASIVNFSEFYEENYEGGKECYRVLNELIGDFDEL   1126
chr22x1sterlet            IPYHVAEQLKVTQSYSKNHDNVGVIFASIVNFSEFYEENYEGGKECYRVLNELIGDFDEL   1138
chr26x2paddlefish         IPYHVAEQLKVTQSYSKNHDNVGVIFASIVNFSEFYEENYEGGKECYRVLNELIGDFDEL   1086
chr26x1paddlefish         IPYHVAEQLKVTQSYSKNHDNVGVIFASIVNFSEFYEENYEGGKECYRVLNELIGDFDEL   1128
chr13x2sterlet            IPYHVAEQLKVTQSYSKNHDNVGVIFASIVNFSEFYEENYEGGKECYRVLNELIGDFDEL   1078
chr13x1sterlet            IPYHVAEQLKVTQSYSKNHDNVGVIFASIVNFSEFYEENYEGGKECYRVLNELIGDFDEL   1120
chr18x1paddlefish         IPYHVAEQLKVTQSYSKNHDNVGVIFASIVNFSEFYEENYEGGKECYRVLNELIGDFDEL   1119
chr18x2paddlefish         IPYHVAEQLKVTQSYSKNHDNVGVIFASIVNFSEFYEENYEGGKECYRVLNELIGDFDEL   1077
                          ************************************************************

                                         A1112
chr22x3sterlet            LNKQQYNNIEKIKTIGATYMAASGLNPSQCQDTSHPHGHLRTLFDFAKEMMCVVDDFNKD   1156
chr22x2sterlet            LNKQQYNNIEKIKTIGATYMAASGLNPSQCQDTSHPHGHLRTLFDFAKEMMCVVDDFNKD   1186
chr22x1sterlet            LNKQQYNNIEKIKTIGATYMAASGLNPSQCQDTSHPHGHLRTLFDFAKEMMCVVDDFNKD   1198
chr26x2paddlefish         LNKQQYSNIEKIKTIGATYMAASGLNTSQCQDTSHPHGHLRTLFDFAKEMMCVVDDFNKD   1146
chr26x1paddlefish         LNKQQYSNIEKIKTIGATYMAASGLNTSQCQDTSHPHGHLRTLFDFAKEMMCVVDDFNKD   1188
chr13x2sterlet            LNKQQYSNIEKIKTIGATYMAASGLNPSQCQDTSHPHRHLRTLFDFAKEMMCVVDDFNKD   1138
chr13x1sterlet            LNKQQYSNIEKIKTIGATYMAASGLNPSQCQDTSHPHRHLRTLFDFAKEMMCVVDDFNKD   1180
chr18x1paddlefish         LNKQQYSNIEKIKTIGATYMAASGLNPSQCQDNSHPHRHLRTLFDFAKEMMCVVDNFNKD   1179
chr18x2paddlefish         LNKQQYSNIEKIKTIGATYMAASGLNPSQCQDNSHPHRHLRTLFDFAKEMMCVVDNFNKD   1137
                          ******.******************* *****.**** *****************:****

                            x
chr22x3sterlet            MLWFNFKLRIGFNHGPLTAGVIGTTKLLYDIWGDTVNIASRMDTTGVECRVQVSEESYRI   1216
chr22x2sterlet            MLWFNFKLRIGFNHGPLTAGVIGTTKLLYDIWGDTVNIASRMDTTGVECRVQVSEESYRI   1246
chr22x1sterlet            MLWFNFKLRIGFNHGPLTAGVIGTTKLLYDIWGDTVNIASRMDTTGVECRVQVSEESYRI   1258
chr26x2paddlefish         MLWFNFKLRIGFNHGPLTAGVIGTTKLLYDIWGDTVNIASRMDTTGVECRVQVSEESYRV   1206
chr26x1paddlefish         MLWFNFKLRIGFNHGPLTAGVIGTTKLLYDIWGDTVNIASRMDTTGVECRVQVSEESYRV   1248
chr13x2sterlet            MLWFNFKLRIGFNHGPLTAGVIGTTKLLYDIWGDTVNIASRMDTTGVECRVQVSEESYRV   1198
chr13x1sterlet            MLWFNFKLRIGFNHGPLTAGVIGTTKLLYDIWGDTVNIASRMDTTGVECRVQVSEESYRV   1240
chr18x1paddlefish         MLWFNFKLRIGFNHGPLTAGVIGTTKLLYDIWGDTVNIASRMDTTGVECRVQVSEESYRV   1239
chr18x2paddlefish         MLWFNFKLRIGFNHGPLTAGVIGTTKLLYDIWGDTVNIASRMDTTGVECRVQVSEESYRV   1197
                          ***********************************************************:

                                                                        ARM
chr22x3sterlet            LKEMGYDFDYRGTVNVKGKGQMKTFLFPKSVDSGMVPQHQLSVSPDIRVQVDGSIGRSPT   1276
chr22x2sterlet            LKEMGYDFDYRGTVNVKGKGQMKTFLFPKSVDSGMVPQHQLSVSPDIRVQVDGSIGRSPT   1306
chr22x1sterlet            LKEMGYDFDYRGTVNVKGKGQMKTFLFPKSVDSGMVPQHQLSVSPDIRVQVDGSIGRSPT   1318
chr26x2paddlefish         LNEMGYDFDYRGTVNVKGKGQMKTFLFPKSVDSGLVPQHQLSVSPDIRVQVDGSIGRSPT   1266
chr26x1paddlefish         LNEMGYDFDYRGTVNVKGKGQMKTFLFPKSVDSGLVPQHQLSVSPDIRVQVDGSIGRSPT   1308
chr13x2sterlet            LNEMGYDFDYRGTVNVKGKGQMKTFLFPKSVDSGMVPQHQLSVSPDIRVQVDGSIGRSPT   1258
chr13x1sterlet            LNEMGYDFDYRGTVNVKGKGQMKTFLFPKSVDSGMVPQHQLSVSPDIRVQVDGSIGRSPT   1300
chr18x1paddlefish         LNEMGYDFDYRGTVNVKGKGQMKTFLFPKSVDSGMVPQHQLSVSPDIRVQVDGSIGRSPT   1299
chr18x2paddlefish         LNEMGYDFDYRGTVNVKGKGQMKTFLFPKSVDSGMVPQHQLSVSPDIRVQVDGSIGRSPT   1257
                          *:********************************:*************************

chr22x3sterlet            EELANMVPSAGGHGLDRTPPQRAAAALEKLELMDSSGHLRAAPVAASSPGNTQRPHPARA   1336
chr22x2sterlet            EELANMVPSAGGHGLDRTPPQRAAAALEKLELMDSSGHLRAAPVAASSPGNTQRPHPARA   1366
chr22x1sterlet            EELANMVPSAGGHGLDRTPPQRAAAALEKLELMDSSGHLRAAPVAASSPGNTQRPHPARA   1378
chr26x2paddlefish         EELANMVPTAGGHGLDRTPPQRAAGALEKLELMDSSGHLRAAPVAAFSPGNTQRPLPTQA   1326
chr26x1paddlefish         EELANMVPTAGGHGLDRTPPQRAAGALEKLELMDSSGHLRAAPVAAFSPGNTQRPLPTQA   1368
chr13x2sterlet            DELANMVPSAGGHGLDRTPPQRAAAAVEKLDVKDSSGHLRAAPFAVSSPGNTQRPLPARD   1318
chr13x1sterlet            DELANMVPSAGGHGLDRTPPQRAAAAVEKLDVKDSSGHLRAAPFAVSSPGNTQRPLPARD   1360
chr18x2paddlefish         DELANMVPSAGGHGLDRTPPQRAAAAMEKLDVKDSSGHLRAAPFAVSSPGNTQRPLPAQA   1317
                          :*******:****************.*:***:: **********.*. ******** *::

chr22x3sterlet            LATPA--NLPATGQGTAEEEEEEEEEEE-------EADELTKLKISESL 1376
chr22x2sterlet            LATPA--NLPATGQGTAEEEEEEEEEEE-------EADELTKLKISESL 1406
chr22x1sterlet            LATPA--NLPATGQGTAEEEEEEEEEEE-------EADELTKLKISESL 1418
chr26x2paddlefish         LATPA--NLPATKQGTAEEEEEEEEEEEEEEEEAAAADELTKLKISESL 1373
chr26x1paddlefish         LATPA--NLPATKQGTAEEEEEEEEEEEEEEEEAAAADELTKLKISESL 1415
chr13x2sterlet            LAAPATRNLPATGQGTAEEEEGKEEEE--------ETDELTKLKISESL 1359
chr13x1sterlet            LAAPATRNLPATGQGTAEEEEGKEEEE--------ETDELTKLKISESL 1401
chr18x1paddlefish         LATTATRNLHAEEE----EGEEEEEEE--------EADELTKLKISESL 1396
chr18x2paddlefish         LATTATRNLHAEEE----EGEEEEEEE--------EADELTKLKISESL 1354
                          **: *  ** *  :    * * :****        :*************
```
